# LncRNAs as new biomarkers to differentiate triple negative breast cancer from non-triple negative breast cancer

**DOI:** 10.18632/oncotarget.7509

**Published:** 2016-02-19

**Authors:** Mingming Lv, Pengfei Xu, Ying Wu, Lei Huang, Wenqu Li, Shanshan Lv, Xiaowei Wu, Xin Zeng, Rong Shen, Xuemei Jia, Yongmei Yin, Yun Gu, Hongyan Yuan, Hui Xie, Ziyi Fu

**Affiliations:** ^1^ Nanjing Maternity and Child Health Medical Institute, Affiliated Nanjing Maternal and Child Health Hospital, Nanjing Medical University, Nanjing, China; ^2^ Department of Pathology, Affiliated Nanjing Maternal and Child Health Hospital, Nanjing Medical University, Nanjing, China; ^3^ First Affiliated Hospital, Nanjing Medical University, Nanjing, China; ^4^ Department of Oncology and Lombardi Comprehensive Cancer Center, Georgetown University Medical Center, Washington, D.C., USA; ^5^ Department of Pharmacology, School of Basic Medical Sciences, Nanjing Medical University, Nanjing, China

**Keywords:** triple negative breast cancer, lncRNA, biomarker, therapy target

## Abstract

Triple negative breast cancer (TNBC) is an aggressive type of breast cancer with high heterogeneity. To date, there is no efficient therapy for TNBC patients and the prognosis is poor. It is urgent to find new biomarkers for the diagnosis of TNBC or efficient therapy targets. As an area of focus in the post-genome period, long non-coding RNAs (lncRNAs) have been found to play critical roles in many cancers, including TNBC. However, there is little information on differentially expressed lncRNAs between TNBC and non-TNBC. We detected the expression levels of lncRNAs in TNBC and non-TNBC tissues separately. Then we analyzed the lncRNA expression signature of TNBC relative to non-TNBC, and found dysregulated lncRNAs participated in important biological processes though Gene Ontology and Pathway analysis. Finally, we validated these lncRNA expression levels in breast cancer tissues and cells, and then confirmed that 4 lncRNAs (RP11-434D9.1, LINC00052, BC016831, and IGKV) were correlated with TNBC occurrence through receiver operating characteristic curve analysis. This study offers helpful information to understand the initiation and development mechanisms of TNBC comprehensively and suggests potential biomarkers for diagnosis or therapy targets for clinical treatment.

## INTRODUCTION

Breast cancer is the leading cause of cancer mortality among women worldwide. The incidence of breast cancer has been increasing by 3% per year in China, which has threatened the health of women and created a great burden on society [1]. During the past decades, insight into the mechanisms of breast cancer has been developing slowly, accompanied the development of biological technology. Based on gene expression profiling, breast cancer has been categorized into four major subtypes: luminal A, luminal B, human epidermal growth factor receptor 2 positive (Her 2 +), and basal-like [2]. According to these categories, developments in clinical treatment strategy, including the foundation of endocrine therapy and Her-2 targeted therapy, have improved the survival levels of breast cancer patients. However, triple negative-breast cancer (TNBC), which is characterized by the lack of an estrogen receptor (ER), progesterone receptor (PR), and Her-2 overexpression, could not benefit from both endocrine therapy and Her-2 targeted therapy [3]. Chemotherapy is the unique systemic treatment for TNBC, although patients with TNBC probably have a worse treatment response and poorer outcomes after chemotherapy compared with the patients with breast cancers of other subtypes [4, 5]. Considering the high heterogeneity of TNBC, it is difficult to confirm which subsets of TNBC patients are likely to respond to specific chemotherapeutics, and there are no reliable biomarkers that could be used as a screening marker. Therefore, it is urgent to identify novel biomarkers and potential therapeutic targets for this aggressive TNBC phenotype.

**Figure 1:**
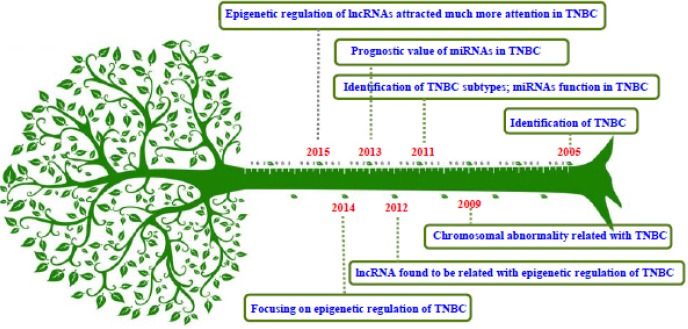
The evolution of exploring the mechanisms of TNBC

During the past decade, TNBC initiation and development have been explored at different molecular levels. 1) The definition of TNBC was changed from the traditional categories of breast cancer (i.e., luminal A, luminal B, basal-like, and Her-2 +) in 2005 [6]; 2) In 2009, TNBC was identified as resulting from chromosomal abnormalities, such as chromosomal band deletion of PTEN/RASA1 and EGFR/VEGFA/FAS overexpression [7, 8]; 3) TNBC was classified into six subtypes according to the intrinsic gene characteristics, including basal-1 and -2, mesenchymal, mesenchymal stem cell-like, immune-modulatory and enriched androgen pathway in the year 2011 [9]; 4) miRNAs were reported to participate in the regulation of TNBC processes in 2011 [10]; 5) lncRNAs were found to be related to epigenetic regulation of TNBC in 2012 [11]; 6) An integrated analysis of six miRNA expression levels (i.e., miR-424, miR-125a-5P, miR-627, miR-579, let-7g, and miR-101) were suggested to indicate a poor outcome in TNBC, and miRNAs were suggested as effective therapeutic targets for TNBC in 2013 [12]; 7) The relationship between TNBC development and epigenetics has attracted much more attention since the year 2014 [13-15]; 8) During 2005-2015, the initiation and development of TNBC was linked to functional genes, proteins, microRNAs, gene methylation, and other factors; however, the mechanisms related to a poor prognosis, heterogeneity, and an aggressive phenotype of TNBC are still unclear (Figure 1).

LncRNAs, which have been a focus of study recently, are segments of RNA that are more than 200 nucleotides in length with little translation capacity (i.e., non-coding RNAs). The total number of lncRNAs might be approximately 410,000 [16-19]. Recently, lncRNAs have attracted much attention in various areas of study to elucidate the complex mechanisms of multiple cellular processes, especially in cancer [20-22]. The functions of lncRNAs mainly include regulation of gene methylation, transcriptional activation, conjugation with mRNAs and miRNAs to affect translation progression and other processes [23-25]. Normally, the relationships between lncRNAs and their neighboring coding genes include sense overlapping, antisense, intronic, divergent, and intergenic interactions [26, 27]. Aberrant expression levels of lncRNAs are related to various malignant biological processes, including carcinogenesis, cell proliferation, apoptosis, migration, invasion, and autophagy [28-34]. Furthermore, Shen et al. and Chen et al. have recently reported differences in lncRNA expressions between TNBC and paired normal tissues [35, 36]. However, there is little information on the differentially expressed lncRNAs between TNBC and non-TNBC tissues. In this study, we aimed to uncover the dysregulated lncRNAs in TNBC (compared with non-TNBC), which might be helpful for understanding the initiation and developmental mechanisms of TNBC comprehensively, and may offer potential biomarkers for diagnosis or therapy targets for clinical treatment.

## RESULTS

### Differential lncRNA expression characters between TNBC and non-TNBC tissues

**Figure 2:**
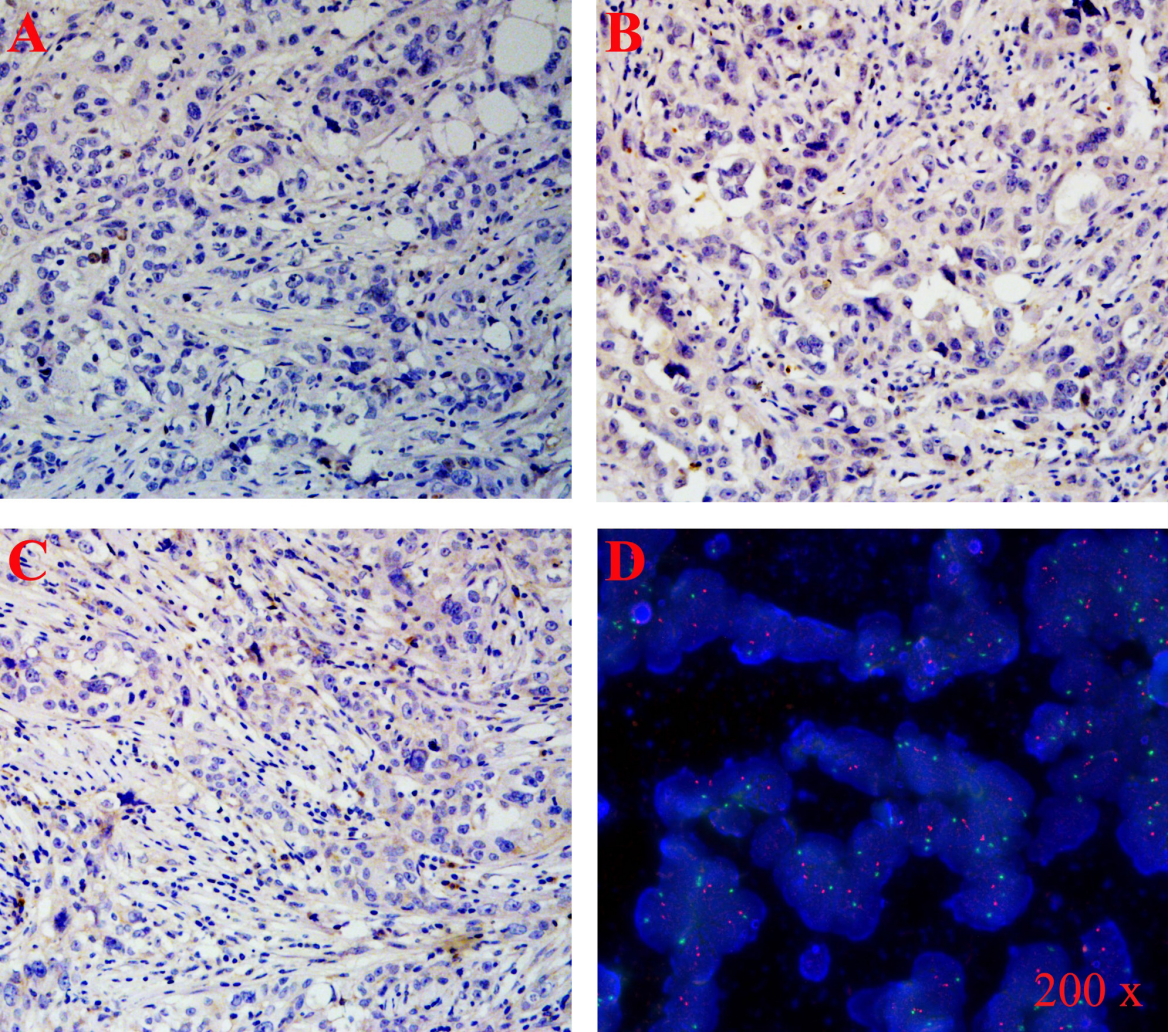
Hormone receptor status was evaluated by IHC or FISH TNBC tissues were identified by ER - **A.**, PR - **B.**, Her 2 - **C.**, and **D.** Generally, the status of Her-2 expression level was evaluated by the ratio of Her-2:centromere of chromosome 17 (i.e., red dots: green dots). The status of Her-2 was defined as negative when the ratio of red dots: green dots < 2.

**Figure 3:**
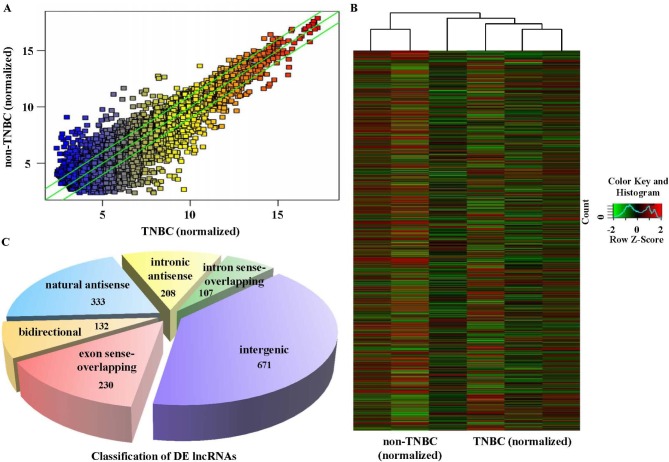
Differential lncRNA expression characteristics between TNBC and non-TNBC tissues The lncRNA microarray showed the differences in lncRNA expression between TNBC and non-TNBC through hot-spot **A.** and cluster mapping **B.** Based on the location relationship of the nearby coding genes, the differentially expressed lncRNAs were classified into several types, which mainly included 333 natural antisense, 208 intronic antisense, 107 intron sense-overlapping, 671 intergenic, 230 exon sense-overlapping, and 132 bidirectional lncRNAs **C.**

In this study, we detected the expression levels of lncRNAs in 3 TNBC and 3 age-matched non-TNBC samples using a high-throughput microarray technique. TNBC cases were identified by immunohistochemistry (IHC) staining of ER, PR and Her-2 (Figure 2A-2C). Fluorescence in situ hybridization (FISH) was performed to confirm the expression level of Her-2 (Figure 2D). Based on the results of microarray analysis, there were 880 lncRNAs up-regulated and 784 down-regulated in the TNBC samples relative to the non-TNBC (Figure 3A-3B), with fold-change filtering (absolute fold-change >2.0), a standard Student's t-test (*p* < 0.05) and multiple hypothesis testing (FDR < 0.05). According to the location relationship of the nearby coding genes, these differentially expressed lncRNAs mainly included 333 natural antisense, 208 intronic antisense, 107 intron sense-overlapping, 671 intergenic, 230 exon sense-overlapping, and 132 bidirectional lncRNAs (Figure 3C).

### Gene Ontology (GO) and pathway analysis of differentially expressed lncRNAs

**Figure 4:**
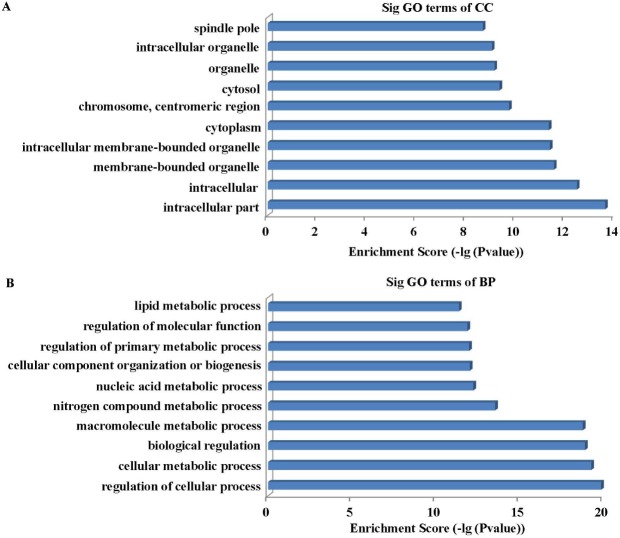

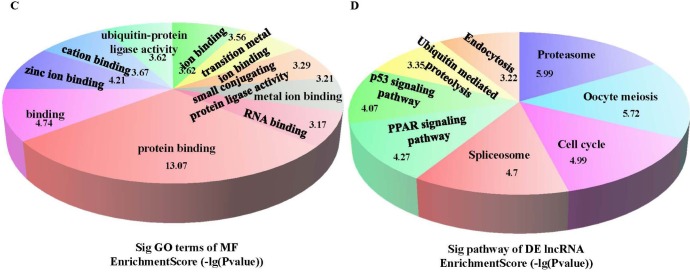
To explore the potential functions of the dysregulated lncRNAs in TNBC, we performed GO and Pathway analysis The GO analysis data showed that these gene products were mainly located in the intracellular region, organelles, membrane-bounded organelles, intracellular membrane-bounded organelles **A.**; the top 10 participating biological processes of targeted genes are listed in Figure 4 **B.** the molecular functions of these genes mainly included binding, protein binding, nuclear binding, and ion binding **C.** The Pathway analysis results indicated that these genes were involved in the PPAR signaling pathway, proteasomes, oocyte meiosis, cell cycle, spliceosome, p53 signaling pathway, ubiquitin mediated proteolysis, and endocytosis **D.**

To explore the potential functions of the dysregulated lncRNAs in TNBC preliminarily, we predicted the target genes of the lncRNAs based on the principles of chromosome location of nearby coding genes and base-pairing. Then we carried out GO analysis for those lncRNAs and target genes (Supplemental material S1). The GO project (http://www.geneontology.org) mainly covers three areas (including Biological Processes, Molecular Function, and Cellular Components), and provides controlled annotations to describe genes and gene products attributed to any organism. The GO analysis results indicated that these gene products were mainly found in the intracellular region, organelles, membrane-bound organelles, and intracellular membrane-bound organelles (Figure 4A). The genes were involved in the biological processes of regulation of cellular processes, cellular metabolic processes, biological regulation, macromolecule metabolic processes, and others (Figure 4B). The molecular functions of these genes included binding, protein binding, nuclear binding, and ion binding (Figure 4C). Meanwhile, the pathway analysis showed that these gene products participate in several signaling pathways, involving PPAR signaling (hsa03320), proteasome (hsa03050), oocyte meiosis (hsa04114), cell cycle (hsa04110), spliceosome (hsa03040), p53 signaling (hsa04115), ubiquitin-mediated proteolysis (hsa04120), and endocytosis (hsa04144) pathways (Figure 4D). The *p*-value (EASE-score, Fisher-*P* value or Hypergeometric-*P* value) denotes the significance of the GO terms enrichment and the pathway correlated to the conditions. The lower the p-value, the more significant the GO term and pathway (*p* < 0.05).

### Discovery of TNBC-associated lncRNAs

**Figure 5:**
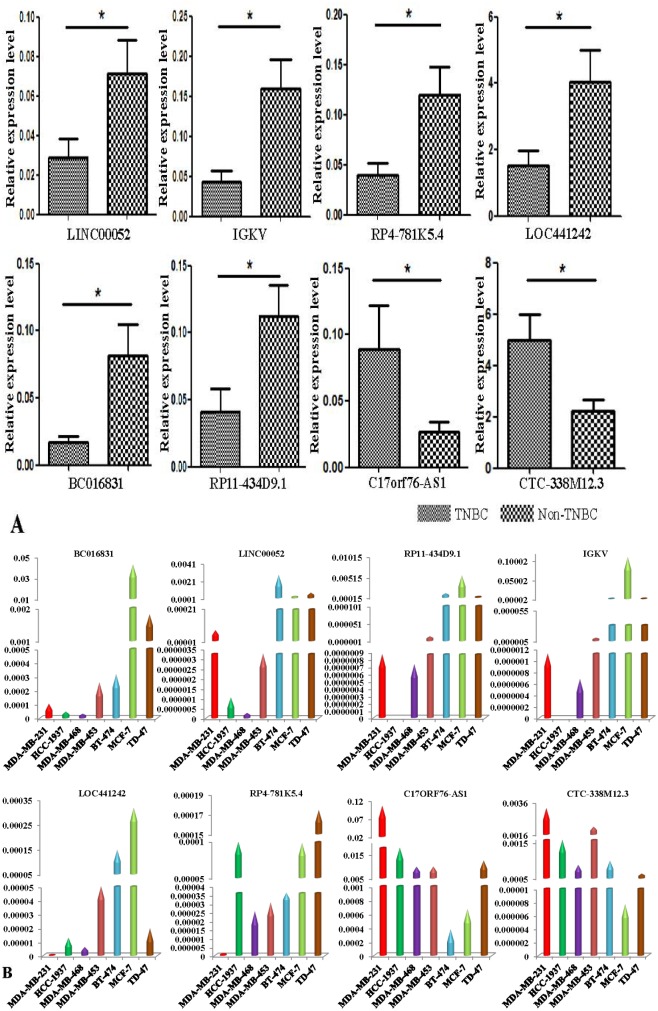
Validation of TNBC-associated lncRNAs First, we validated the expression levels of the dysregulated lncRNAs in TNBC and non-TNBC tissues, and 8 lncRNAs were dysregulated predominantly in TNBC samples corresponding to non-TNBC samples **A.** Then we assessed the expression levels of these 8 lncRNAs in TNBC cells and non-TNBC cells separately. The results showed that BC016831, IGKV, LINC00052, and RP11-434D9.1 were down-regulated congruously in all 4 TNBC cells **B.** (Each experiment was repeated in triplicate independently, *p < 0.05).

In the present study, we validated the expression levels of the dysregulated lncRNAs, not only in 46 samples, but also in MDA-MB-231/HCC-1937/MDA-MB-468/MDA-MB-453 TNBC cells and BT-474/MCF-7/TD-47 non-TNBC cells. The differentially expressed lncRNAs were selected by fold-change filtering (absolute fold-change >2.0), a standard Student's t-test (*P* < 0.05), multiple hypothesis testing (FDR < 0.05), and at least 1 out of 2 groups that had flags in Present or Marginal. Finally, we identified 70 lncRNAs that had significant differential expression levels in TNBC as compared with non-TNBC controls, and the primers of the lncRNAs are listed in Supplementary material S2. Of these 70 dysregulated lncRNAs, 38 lncRNAs were found up-regulated and 32 lncRNAs down-regulated. The qRT-PCR results showed that, compared with non-TNBC tissues, C17orf76-AS1 and CTC-338M12.3 were dominantly up-regulated in TNBC tissues; on the other hand, RP11-434D9.1, IGKV, LINC00052, BC016831, RP4-781K5.4, and LOC441242 were obviously down-regulated (Figure 5A). Generally, the expression patterns of these deregulated lncRNAs in TNBC cell lines appeared to be in accordance with the results in tissues, compared with the non-TNBC cell lines. However, there are some differences between the lncRNA expression patterns of tissues and cell lines. Briefly, compared with the non-TNBC cell group, only 4 lncRNAs (BC016831, IGKV, LINC00052, and RP11-434D9.1) were down-regulated congruously in all 4 TNBC cells (Figure 5B).

### Predictive power of lncRNAs for diagnosis of TNBC

**Figure 6:**
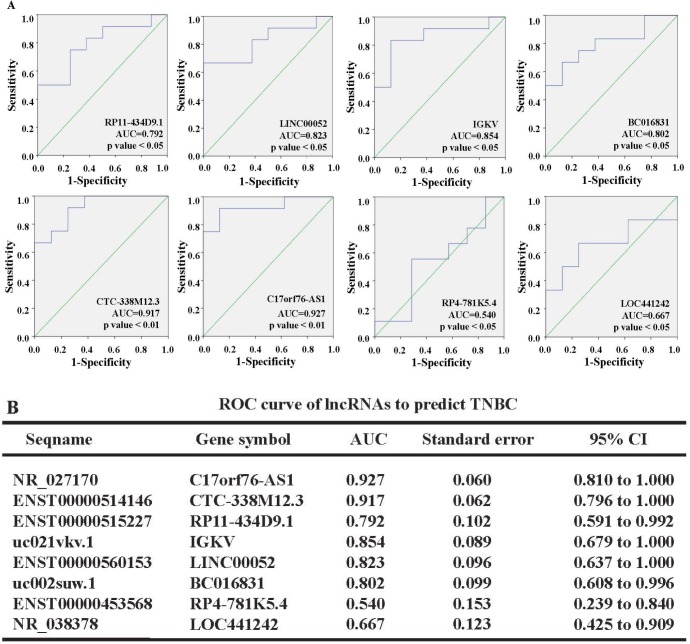
ROC curves were created to evaluate the power of 8 dysregulated lncRNAs for predicting TNBC The data showed that RP11-434D9.1, LINC00052, IGKV, and BC016831 could be the potential biomarkers to differentiate TNBC from non-TNBC, while CTC-338M12.3, C17orf76-AS1, RP4-781K5.4 and LOC441242 might not be powerful tools for predicting TNBC **A.** Complete information on the ROC curves **B.**

To evaluate the power of 8 dysregulated lncRNAs for predicting TNBC, we performed receiver operating characteristic (ROC) curve analysis. The samples were divided into two groups based on the molecular phenotype, including TNBC and non-TNBC. The cut-off value for each lncRNA was determined with Kaplan-Meier analyses in our cohort (41). The results showed that the ROC curve for RP11-434D9.1 had an area under the curve (AUC) of 0.792 (95% CI = 0.591 to 0.992); the LINC00052 AUC was 0.823 (95% CI = 0.637 to 1.000); the IGKV AUC was 0.854 (95% CI = 0.679 to 1.000); the BC016831 AUC was 0.802 (95% CI = 0.608 to 0.996); the CTC-338M12.3 AUC was 0.917 (95% CI = 0.796 to 1.000); the C17orf76-AS1 AUC was 0.927 (95% CI = 0.810 to 1.000); the RP4-781K5.4 AUC was 0.54 (95% CI = 0.239 to 0.840); and the LOC441242 AUC was 0.667 (95% CI = 0.425 to 0.909; Figure 6),. According to the data, RP11-434D9.1, LINC00052, IGKV, and BC016831 could be potential biomarkers to differentiate TNBC from non-TNBC, while RP4-781K5.4, CTC-338M12.3, C17orf76-AS1 and LOC441242 might not be powerful tools for predicting TNBC.

## DISCUSSION

TNBC has high heterogeneity and is a more aggressive breast cancer, which has attracted much attention in clinical and basic research areas during the past decade [42]. Compared with non-TNBC breast cancer, TNBC patients neither benefited from efficient endocrine therapy nor the Her-2 targeted therapy. Although TNBC seems to be more sensitive to chemotherapy, it appears that TNBC has a higher recurrence risk and poorer outcomes. As a spreading development in individual medicine, the small difference between TNBC and non-TNBC must be elucidated, in addition to hormone related receptors.

During the post-genome period, lncRNAs have become a focus of study in the regulation of histone acetylation, gene methylation, post-transcription translation, and other biological processes [23-25]. Recently, many more lncRNAs have been confirmed to play critical roles in regulating the physiological behavior of malignant cancers, including breast, pancreatic, gastric, lung, and others. Predominantly, lncRNAs have been shown to regulate cancer cell viability, apoptosis, invasion and metastasis [28-34]. As is well-known, HOTAIR could regulate breast cancer proliferation and chemo-resistance as an oncogenic lncRNA [43-47]. Since the dysregulated lncRNAs between TNBC and adjacent normal tissues have been identified [35, 36], there is still no information on the differentially expressed lncRNAs between TNBC and non-TNBC tissues. In this study, we aimed to improve the understanding of lncRNA expression characteristics in TNBC.

According to the results of microarray assays, there were 880 lncRNAs up-regulated and 784 down-regulated in TNBC relative to the non-TNBC samples (Figure 3A-3B), including 333 natural antisense and 671 intergenic (Figure 3C) lncRNAs, which are most possibly the regulating elements of biological processes until now [16, 19]. The results showed that the aggressive behaviors of TNBC are probably related to these differentially expressed lncRNAs. To predict the potential functions of these dysregulated lncRNAs, we carried out GO analysis. We mainly enriched the lncRNAs that regulate several biological processes (Figure 4B), and the top 3 included regulation of cellular processes, cellular metabolic processes, and biological regulation, which are closely related to the malignancy of cancer. We also classified the potential functions into 10 categories by analyzing the target gene pool (Figure 4C), including protein binding, zinc ion binding, cation binding, ubiquitin-protein ligase activity, ion binding, transition metal ion binding, small conjugation protein ligase activity, metal ion binding, and RNA binding. Interestingly, we found the dysregulated lncRNAs could be mainly divided into two groups, binding activity and ligase activity, which means these dysregulated lncRNAs might play important roles in biological processes by regulating the cell skeleton like a kind of scaffolding. Moreover, pathway analysis results showed that these dysregulated lncRNAs mainly participated in the signaling pathways (Figure 4D). The cell cycle, p53 signaling pathway, PPAR signaling pathway, and ubiquitin mediated proteolysis have been well studied in the initiation and development of breast cancer. What is amazing is that over 500 dysregulated lncRNAs were involved in the oocyte meiosis pathway and, by coincidence, Browaeys-Poly et al. found that oocytes could appear consistently with MDA-MB-231 TNBC cells in some way [48]. Meanwhile, Shen et al. also reported that many dysregulated lncRNAs in TNBC (compared with normal breast tissues) were involved in the oocyte meiosis pathway [35]. Whether there is a correlation between oocyte development and TNBC occurrence is an interesting question that should be studied in the future. In summary, these differentially expressed lncRNAs partially indicated the molecular characteristics of TNBC, relative to the non-TNBC tissues, and these lncRNAs might be individual biomarkers for diagnosis or therapeutic targets for clinical TNBC therapy.

The expression levels of these dysregulated lncRNAs were confirmed in 46 samples, despite the heterogeneity of TNBC and individual differences. The differentially expressed lncRNAs were selected as described previously, and the qRT-PCR results showed that (Figure 5A), compared with non-TNBC tissues, NR_027168 (C17orf76-AS1) and ENST00000514146 (CTC-338M12.3) were dominantly up-regulated in TNBC tissues; otherwise, ENST00000515227 (RP11-434D9.1), uc021vkv.1 (IGKV), ENST00000560153 (LINC00052), NR_038378 (LOC441242), ENST00000453568 (RP4-781K5.4), and uc002suw.1 (BC016831) were clearly down-regulated. The expression levels of all 8 dysregulated lncRNAs were confirmed in non-TNBC cell lines and TNBC cell lines separately (Figure 5B). Compared with the non-TNBC cell group, only 4 lncRNAs (BC016831, IGKV, LINC00052, and RP11-434D9.1) were down-regulated congruously in all 4 TNBC cells.

C17orf76-AS1 is the natural antisense of gene C17orf76, and CTC-338M12.3 is a bidirectional lncRNA of the targeted gene TRIM52, while all of the 6 down-regulated were all intergenic lncRNAs. During the past years, intergenic and antisense lncRNAs have been shown to regulate cell behaviors in many cancers [49-51]. Of particular interest, long noncoding RNA HOTAIR has been suggested to be related to the methylation level of downstream intergenic CpG islands in breast cancer [52], and Kim et al. demonstrated that HOTAIR could be a negative prognostic factor in pancreatic cancer [53]. Except for regulating gene methylation, lncRNAs could be endogenous inhibitors that reverse the effects of miRNA [54, 55]. The complex modes of function and great abundance make lncRNAs very interesting in research endeavors today. These dysregulated lncRNAs may be novel biomarkers for the diagnosis of TNBC malignancy and could be potential targets for individual therapy of TNBC patients in the future.

We further performed ROC analysis to evaluate the power of these 8 lncRNAs to differentiate TNBC from non-TNBC in our cohort. The data indicated that RP11-434D9.1, LINC00052, IGKV, BC016831, CTC-338M12.3 and C17orf76-AS1 could be potential biomarkers. Based on the expression levels of these 8 lncRNAs in TNBC tissues and cell lines, we concluded that RP11-434D9.1, LINC00052, BC016831, and IGKV might be potential biomarkers for diagnosis or therapy targets of clinical treatment for TNBC. Although the differentially expressed lncRNAs between TNBC and paired normal breast tissues were explored, these lncRNAs might not have an association with TNBC malignancy compared with non-TNBC upon further study. Our data showed differences in lncRNA expression signatures between TNBC and non-TNBC, and these 6 lncRNAs maybe the potential targets for individual therapy; however, it is necessary to validate these results in larger cohorts and elucidation of the underlying mechanisms is urgently needed.

## MATERIALS AND METHODS

### Cell lines and reagents

Human TNBC cells (MDA-MB-231, HCC-1937, MDA-MB-468, and MDA-MB-453) and non-TNBC cells (BT-474, MCF-7, and TD-47) were all obtained from the American Type Culture Collection (ATCC). Cells were cultured in RPMI-1640 medium (Gibco) or L-15 (Gibco) supplemented with 10% (v/v) fetal bovine serum (Gibco), 100 U/mL streptomycin (Gibco), and 100 U/mL penicillin (Gibco) at 37°C in a humidified atmosphere containing 5% carbon dioxide. All reagents were purchased from Sigma−Aldrich unless otherwise mentioned.

### Tissue collection

Female primary breast cancer tissue samples were obtained from the Breast Department of Nanjing Maternal and Child Health Hospital (Nanjing, China). In all, 14 primary TNBC cases (age 55.2 ± 8.8 years) were involved in this study, and 32 age-matched primary non-TNBC cases (age 53.3 ± 9.2 years) were also selected. The tissues were collected, washed, quick-frozen in liquid nitrogen after surgery, and the histopathological diagnoses were all confirmed as breast cancer. Informed consent about the use of these samples was obtained from each patient. Ethical approval was obtained from the hospital ethics committee.

### IHC

A traditional pathology diagnosis was carried out to detect the ER, PR and Her-2 status of breast cancer samples [37]. The molecular subtypes of these breast cancer patients were defined by IHC staining of ER, PR, and Her-2. The Allred scoring method was employed to classify the expression status of ER and PR. Generally, the proportion score showed the estimated percentage of tumor cells staining positive (0 = 0%; 1 = 1%; 2 ≥ 1 to 10%; 3 ≥ 10 to 33%; 4 ≥ 33 to 66%; 5 ≥ 67%), and the intensity of staining was scored as follows: 1 = weakly; 2 = moderately; and 3 = strongly. The total score was derived from the following equation, with a score of 0 being negative and a score of 2 to 8 being positive. Membranous staining was scored for Her-2/neu according to the HercepTest (Dako) as follows: 0 = negative; 1 = weak incomplete membranous staining of >10% cells (negative); 2 = weak to moderate complete membranous staining of >10% of cells (equivocal-fluorescence *in situ* hybridization was used to assess amplification in these cases); 3 = strong complete membranous staining of >30% of cells (positive). A standard FISH was performed to confirm the expression level of Her-2. Generally, her-2 was detected by a Texas-Red labelled probe (red dot); meanwhile, the centromere of chromosome 17 was detected by a FITC labelled probe (green dot). The status of the Her-2 expression level was evaluated by the ratio of Her-2:centromere of chromosome 17 (i.e., red dots:green dots). The status of Her-2 was defined as positive if the ratio was ≥2; otherwise it was defined as negative.

### Total RNA extraction

Tissue samples and cells were dissolved in TRIzol reagent and total RNAs were extracted according to the manufacturer's protocol (Invitrogen, CA, USA). Quantification and quality checks were performed with Nano-drop and an Agilent 2100 Bio-analyzer (Agilent Technologies), respectively.

### LncRNA expression profiling

For lncRNA expression profiling, we profiled 3 TNBC patient samples and 3 non-TNBC patient samples with Arraystar lncRNA microarrays as described previously [38]. Briefly, RNA was purified from 1 mg of total RNA after removal of rRNA (mRNA-ONLY Eukaryotic mRNA Isolation Kit, Epicentre). Then, each sample was amplified and transcribed into fluorescent RNA along the entire length of the transcripts without bias utilizing a random priming method. The labeled RNAs were hybridized onto the Human LncRNA Array v3.0 (Agilent SureHyb). After washing, the arrays were scanned by the Agilent LncRNA Microarray Scanner, and Agilent Feature Extraction software (11.0.1.1) was used to subsequently collect the raw values of the microarray probe signal. Finally, Agilent GeneSpring GX v12.1 software was employed to normalize the values, and then, lncRNAs and mRNAs, which had at least 1 out of 2 groups have flags in Present or Marginal, were chosen for further data analysis. Additionally, hierarchical clustering and combined analyses were performed using homemade scripts.

### LncRNA classification pipeline

To elucidate the lncRNA expression pattern in the probe name-centric TNBC gene expression data, we used a common lncRNA classification pipeline to clarify the lncRNAs represented on the Affymetrix microarray following the strategies below. First, the annotations of microarray data involved the probe name, seqname, gene symbol, gene title, source, chromosome location, sequence, and other informative items for the specific probe set. Second, the seqname was assigned with a GENCODE ID, RefSeq database ID, and/or Ensembl gene ID. For the seqname with GENCODE IDs, we labeled these as “ENST”. For the seqname with Refseq IDs, we labeled these as “NR_” (NR means non-coding RNA). For the seqname with Ensembl gene ID, we labeled these as “uc” (http://www.genome.ucsc.edu/). Third, we filtered the seqname obtained in step 2 by filtering out pseudogenes, rRNAs, microRNAs and other short RNAs including tRNAs, snRNAs and snoRNAs [39].

### GO and pathway analysis

Differentially expressed lncRNAs were identified by fold-change filtering (absolute fold-change >2.0), a standard Student's t-test (P < 0.05) and multiple hypothesis testing (FDR < 0.05) [40]. GO and pathway analysis for differentially expressed lncRNAs (antisense lncRNA, intronic lncRNA, enhancer lncRNA, and lincRNAs) were used to identify the significantly enriched biological terms and pathways. GO terms and pathway enrichment analysis were both based on the database for annotation, visualization, and integrated discover (DAVID) Bioinformatics Resources (http://david.abcc.ncifcrf.gov/), and the result of pathway enrichment analysis was confirmed by the online database of the Kyoto Encyclopedia of Genes and Genomes (KEGG) (http://www.kegg.jp/). The potential functions of these differentially expressed lncRNAs were identified by functional annotation clustering and were then ranked by enrichment scores.

### Validation of differentially expressed lncRNA by quantitative real-time PCR

The total RNA of sample tissues and cells was extracted and reverse transcribed into cDNA with random primers with a Reverse Transcription Kit (Takara) according to the manufacturer's instructions. Standard qRT-PCR was performed to confirm the expression levels of differentially expressed lncRNAs with the Applied Biosystems ViiA 7 Sequence Detection System (ABI ViiA 7 SDS, USA) following the manufacturer's guidelines. Briefly, the mixture of samples was incubated at 95°C for 10 min for an initial denaturation, followed by 40 PCR cycles of incubation at 95°C for 15 s, 60°C for 30 s, and then 72°C for 30 s. The specific primer sequences for qRT-PCR are listed in Supplementary material S2. Each sample analysis was performed in triplicate. The expression levels of lncRNAs were normalized to internal control GAPDH, and then calculated with the 2^−ΔCT^ method.

### Statistical analysis

The differences in lncRNA levels were determined with an ANOVA test and multiple hypothesis testing. The sensitivity and specificity were analyzed according to the standard formulas. ROC curves were established for discriminating patients with or without TNBC. The optimal sensitivity and specificity from ROC curves were installed by the standard method. All the p-values are two-sided and *p* < 0.05 was considered statistically significant. Computer-based calculations were conducted using SPSS version 20.0 (SPSS Inc., Chicago).

## SUPPLEMENTARY MATERIAL








