# Supplementary material for: LncRNAs as new biomarkers to differentiate triple negative breast cancer from non-triple negative breast cancer
Source: Oncotarget. 2016 Feb 19;7(11):13047–59. doi: 10.18632/oncotarget.7509 (PMC4914340; doi:10.18632/oncotarget.7509)
Supplement: Supplementary file 1 [file oncotarget-07-13047-s001.pdf]

#Column "GO.ID" stands for the ID of gene ontology term
#Column "Term" stands for the name of gene ontology term
#Column "Ontology" stands for the ontology of GOID belongs to
#Column "Count" stands for the number of DE genes associated with the listed GOID
#Column "Pop.Hits" stands for the number of background population genes associated with the listed GO
#Column "List.Total" stands for the total number of DE genes
#Column "Pop.Total" stands for the total number of background population genes
#Column "Fold.Enrichment" stands for the Fold Enrichment value of the GOID, it equals (Count/Pop.Hits
#Column "Pvalue" stands for the significance testing value of the GOID
#Column "FDR" stands for the false discovery rate of the GOID, using Benjamini & Hochberg (1995) me
#Column "Enrichment.Score" stands for the Enrichment Score value of the GOID, it equals (-log10(Pvalu
#Column "GENES" stands for the DE genes associated with the GOID

GO.ID	Term	Ontology	Count	Pop.Hits	List.Total	Pop.Total	Fold.Enric	Pvalue	FDR
GO.IDTermOntologyCountPop.HitsList.TotalPop.TotalFold.EnrichmentPvalueFDREnrichment.ScoreGENES									
GO:00027	cell	cycleBiolog	process81659570145863.145291911.34528E-204.92641E-1719.87118784PRC						
GO:00224	cycle	processBio	process95882570145862.7562358285.21054E-209.54049E-1719.28311756PR						
GO:00070	cycleBiolog	process1121167570145862.4558877911.24672E-191.52182E-1618.90423271PRC1//KIF23							
GO:00224	cycle	phaseBiolo	process84730570145862.9445421771.67752E-191.53577E-1618.77533291PR						
GO:00027	phaseBiolo	process56460570145863.1152402752.82708E-142.07055E-1113.54866224PRC1//KIF23//N							
GO:00517	of	cell	cycleBiolog	process62584570145862.7166906995.76313E-133.51743E-1012.23					
GO:00105	of	cell	cycle	processBio	process44331570145863.4016218799.2015E-134.68159				
GO:00000	phase	of	mitotic	cell	cycleBiolog	process44332570145863.391376031.02274E			
GO:00513	divisionBiol	process47375570145863.2072140351.30843E-125.32384E-1011.88325098NUSAP1//KIF2							
GO:00073	of	mitotic	cell	cycleBiolog	process37254570145863.7276004974.05864E-121.4862				
GO:00002	divisionBiol	process42321570145863.3481554355.24653E-121.60107E-0911.28012769NDC80//ZWINT							
GO:00070	process42321570145863.3481554355.24653E-121.60107E-0911.28012769NDC80//ZWINT//MAD2L1/								
GO:00000	cycle	checkpoint	process33214570145863.9460403341.25739E-113.54197E-0910.90053016H2A						
GO:00482	fissionBiolo	process42333570145863.2275011851.75722E-114.59639E-0910.7551735NDC80//ZWINT/							
GO:00711	of	cell	cycle	arrestBiolo	process33223570145863.7867831013.93265E-119.3965				
GO:00070	cycle	arrestBiolo	process41328570145863.1986842114.10554E-119.39655E-0910.38662995UBE						
GO:00070	cell	cycle	checkpoint	process24126570145864.8741854649.48713E-112.04364E-0810.02					
GO:00457	regulation	of	cell	cycleBiolog	process44384570145862.9321271931.40755E-102.8635				
GO:00311	complex-de	proteasoma	ubiquitin-de	protein	catabolic	processBio	process1882570145865.617201		
GO:00070	segregation	process21114570145864.7138504162.69319E-094.93122E-078.569733551NDC80//ZWINT							
GO:00900	regulation	of	cell	cycle	processBio	process19105570145864.630476192.09047E			
GO:00513	of	ligase	activityBiol	process1895570145864.8485318562.32979E-083.87804E-067.6326					
GO:00513	of	mitotic	cell	cycleBiolog	process36331570145862.7831451742.7665E-084.40475				
GO:00002	prometapha	process1786570145865.0583843332.95546E-084.50954E-067.529374834NDC80//CENPA							
GO:00442	macromole	catabolic	processBio	process48527570145862.3307300514.18114E-086.12453E-067.378					
GO:00513	process36339570145862.7174662325.11542E-087.20487E-067.291118969CDKN3//ORC6//INHBA//PC								
GO:00000	transition	of	mitotic	cell	cycleBiolog	process24172570145863.5706242355.95508			
GO:00069	organizatio	process1071648570145861.6614524786.15612E-088.05132E-067.210693148PRC1//KIF23							
GO:00514	of	ubiquitin-pr	ligase	activityBiol	process1791570145864.7804511287.11493E-088.98443				
GO:00513	regulation	of	ligase	activityBiol	process1682570145864.9930680369.13925E-081.1156E				
GO:00718	component	organizatio	at	cellular	levelBiolog	process1522632570145861.477811551.2841			
GO:00718	component	organizatio	or	biogenesis	at	cellular	levelBiolog	process156272257014	
GO:00070	organizatio	process1355570145866.0484210531.38715E-071.53166E-056.857878083PRC1//KIF23//N							
GO:00165	ubiquitinati	process36353570145862.6096913671.42207E-071.53166E-056.847078259FBXW11//UBR							
GO:00002	cytoskeleto	organizatio	process25195570145863.2807017541.70412E-071.783E-056.76849887PRC1//						
GO:00324	modificatio	by	small	protein	conjugation	process37375570145862.524828072.20163E			
GO:00706	modificatio	by	small	protein	conjugation	or	removalBio	process404245701458	

GO:00514	of	ubiquitin-pr	ligase	activity	involved	in	mitotic	cell	cycleBiolog
GO:00431	ubiquitin-de	protein	catabolic	processBio	process24185570145863.3197155052.40684E-072.2599				
GO:00514	regulation	of	ubiquitin-pr	ligase	activityBiol	process1578570145864.9210526322.8175E-			
GO:00104	protein	catabolic	processBio	process24189570145863.2494569763.59608E-073.21192E-056.444					
GO:00199	protein	catabolic	processBio	process34340570145862.5589473685.03494E-074.23682E-056.298					
GO:00436	macromole	catabolic	processBio	process34340570145862.5589473685.03494E-074.23682E-056.298					
GO:00514	regulation	of	ubiquitin-pr	ligase	activity	involved	in	mitotic	cell
GO:00065	protein	catabolic	processBio	process33334570145862.5283012929.73479E-077.82596E-056.011					
GO:00516	involved	in	cellular	protein	catabolic	processBio	process34350570145862.485834		
GO:00070	spindle	organizatio	process822570145869.3052631581.01929E-067.94179E-055.99170225PRC1//						
GO:00512	organizatio	process49610570145862.0555478861.33294E-060.0001016925.87519036NDC80//ZWINT							
GO:00442	protein	catabolic	processBio	process34356570145862.4439384981.44705E-060.0001081455.83					
GO:00090	catabolic	processBio	process51649570145862.010883141.53688E-060.0001125615.813359983DCP						
GO:00313	of	protein	ubiquitinati	process19142570145863.4239436622.70206E-060.0001940185.56					
GO:00160	component	organizatio	process1773336570145861.3577148812.77911E-060.0001957135.556094531F						
GO:00069	damage	response,	signal	transductio	by	p53	class	mediator	resulting
GO:00723	transductio	involved	in	cell	cycle	checkpoint	process1261570145865.033994		
GO:00724	transductio	involved	in	DNA	integrity	checkpoint	process1261570145865.033994		
GO:00724	transductio	involved	in	G1/S	transition	checkpoint	process1261570145865.033994		
GO:00724	transductio	involved	in	mitotic	cell	cycle	checkpoint	process12615701458	
GO:00724	transductio	involved	in	DNA	damage	checkpoint	process1261570145865.033994		
GO:00724	transductio	involved	in	mitotic	cell	cycle	G1/S	transition	DNA
GO:00724	transductio	involved	in	mitotic	cell	cycle	G1/S	checkpoint	process12
GO:00718	component	organizatio	or	biogenesis	process1813442570145861.3456405393.61529E-060.00				
GO:00313	regulation	of	protein	ubiquitinati	process16108570145863.7910331384.34971E-060.0002				
GO:00711	regulation	of	cell	cycle	arrestBiolo	process1265570145864.7242105266.84325E			
GO:00315	checkpoint	process936570145866.3973684217.29679E-060.0004175135.136868017CENPF//UBE2C/							
GO:00301	catabolic	processBio	process36418570145862.2038781167.7279E-060.0004353785.111938364ISG1						
GO:00718	complex	subunit	organizatio	process17126570145863.4525480378.03955E-060.0004460735.09					
GO:00070	processBio	process29303570145862.4491575478.24898E-060.0004508625.083599923PRC1//KIF23//							
GO:00315	cell	cycle	G1/S	transition	checkpoint	process1378570145864.2649122819.24194E			
GO:00717	transition	checkpoint	process1378570145864.2649122819.24194E-060.0004904935.034236862PSM						
GO:00514	regulation	of	ubiquitin-pr	ligase	activity	involved	in	mitotic	cell
GO:00002	transition	of	mitotic	cell	cycleBiolog	process1379570145864.2109260491.06737E			
GO:00442	catabolic	processBio	process831329570145861.5981386881.09034E-050.0005545614.962436863W						
GO:00315	cell	cycle	G1/S	transition	DNA	damage	checkpoint	process12685701458	
GO:00711	conformati	changeBiol	process19157570145863.0968152871.19716E-050.000592434.921849564CEN						
GO:00069	transportBi	process26262570145862.5394134191.27573E-050.0006228954.894242463RPS15//IFNG//							
GO:20000	of	G1/S	transition	of	mitotic	cell	cycleBiolog	process13815701458	
GO:00511	transportBi	process26264570145862.5201754391.45957E-050.0006941514.835773704RPS15//IFNG//							
GO:00070	of	mitosisBiol	process1382570145864.0568677791.62067E-050.000751254.79030664CENP						
GO:00517	of	nuclear	divisionBiol	process1382570145864.0568677791.62067E-050.000751254.7903					
GO:00650	complex	assemblyB	process16120570145863.4119298251.72015E-050.0007845624.764434539RA						
GO:00513	regulation	of	ligase	activityBiol	process1271570145864.3249814681.7568E-050.000784				
GO:00514	regulation	of	ubiquitin-pr	ligase	activityBiol	process1271570145864.3249814681.7568E-			
GO:00346	macromole	complex	subunit	organizatio	process44578570145861.9479876161.81776E-050.0008				
GO:00347	organizatio	process15109570145863.5214872042.14235E-050.0009339624.669109756CENPA//OIP5/							
GO:00063	packagingE	process17136570145863.1986842112.23711E-050.0009637984.650313499CENPA//OIP5//							
GO:00063	assemblyB	process1499570145863.618713452.93906E-050.0012420884.531791844CENPA//OIP5//C							
GO:00082	deathBiolog	process861429570145861.5400243082.98107E-050.0012420884.525628126IFI6//SOD2//S							
GO:00485	regulation	of	cellular	processBio	process1222209570145861.4132710682.98481E-050.00				
GO:00162	process861431570145861.5378719343.13773E-050.0012910544.503384031IFI6//SOD2//SYCP2//TRI								
GO:00065	of	cellular	amino	acid	metabolic	processBio	process1053570145864.828202		
GO:00332	of	cellular	amine	metabolic	processBio	process1164570145864.3981907893.33446E			

GO:00125	cell	deathBiolo	process801311570145861.5615239473.64757E-050.0014518924.437996143IFI						
GO:00442	protein	metabolic	processBio	process1482810570145861.3477729913.97731E-050.0015661194.4					
GO:00008	chromatid	segregatio	process944570145865.2342105264.15658E-050.0016192974.381263857NDC8						
GO:00160	metabolic	processBio	process39510570145861.9568421054.94979E-050.0018865164.305413239DC						
GO:00069	process791302570145861.5526639184.98493E-050.0018865164.302341255IFI6//SOD2//HMGB2//CY								
GO:00315	integrity	checkpoint	process15117570145863.2807017544.99705E-050.0018865164.301286108H2A						
GO:00314	assemblyB	process14104570145863.4447368425.14141E-050.0018979724.288918037CENPA//OIP5//							
GO:00480	signalingBi	process1167570145864.2012568745.18288E-050.0018979724.28542857NDC80//UBE2C//							
GO:00480	lipid-media	signalingBi	process1167570145864.2012568745.18288E-050.0018979724.28542857NDC8						
GO:00901	regulation	of	kidney	developme	process5125701458610.66228075.64373E-050.0020462				
GO:00099	processBio	process51512380570145861.0645055695.8057E-050.0020843594.236145554BRIX1//KPN							
GO:00069	to	DNA	damage	stimulusBio	process38501570145861.9409181647.34664E-050.0026				
GO:00430	chromatin	remodeling	process728570145866.3973684217.75625E-050.0027310954.110348223CENP						
GO:00511	exportBiolo	process1395570145863.5017174527.97159E-050.0027801884.098454837RPS15//HNRNP							
GO:00303	damage	response,	signal	transductio	by	p53	class	mediatorBi	process139
GO:00485	regulation	of	biological	processBio	process1282399570145861.3653408228.97426E-050.00				
GO:00062	metabolic	processBio	process45638570145861.8049001819.19544E-050.0031179354.036427471KPI						
GO:00000	damage	checkpoint	process14110570145863.2568421059.58348E-050.0032196984.018476645PSI						
GO:00090	processBio	process911595570145861.4599637020.0001229610.0040934713.910233988WIPI2//ATG1							
GO:00516	localization	process841445570145861.487554180.0001248280.0041182073.903687224RPS15//IFNG//							
GO:00000	phase	of	mitotic	cell	cycleBiolo	process15128570145862.9987664470.00014			
GO:00322	regulation	of	cellular	protein	metabolic	processBio	process29354570145862.0963128		
GO:00063	replication-	nucleosom	assemblyB	process622570145866.9789473680.0001514720.0047818243.8196					
GO:00340	nucleosom	assembly	at	centromere	process622570145866.9789473680.0001514720.004781				
GO:00347	replication-	nucleosom	organizatio	process622570145866.9789473680.0001514720.0047818243.8196					
GO:00313	regulation	of	protein	ubiquitinati	process1288570145863.4894736840.0001541080.00482				
GO:00000	sister	chromatid	segregatio	process841570145864.9930680360.0001580340.0049044133.8012					
GO:00427	transductio	in	response	to	DNA	damageBio	process14117570145863.061988		
GO:00516	of	localization	in	cellBiologic	process771313570145861.5006774360.0001877690.005				
GO:00711	assembly	checkpoint	process732570145865.5976973680.0001924850.0057649453.715603624CENP						
GO:00711	cell	cycle	spindle	checkpoint	process732570145865.5976973680.0001924850.005764				
GO:00305	regulation	of	BMP	signaling	pathwayBio	process515570145868.5298245610.0001940			
GO:00440	component	biogenesis	process741251570145861.5136858930.0001952080.0057649453.709501491B						
GO:00063	assembly	or	disassemb	process16147570145862.7852488360.0002042540.0059838393.68					
GO:00513	phaseBiolo	process15134570145862.8644933230.0002351350.0068338463.628682597PSMA8//ORC6							
GO:00330	of	organelle	organizatio	process25295570145862.1685994650.0002413820.0069601783.61					
GO:00310	remodeling	at	centromere	process624570145866.3973684210.0002556250.0072565773.5923					
GO:00901	of	kidney	developme	process624570145866.3973684210.0002556250.0072565773.5923					
GO:00346	macromole	complex	assemblyB	process33440570145861.9192105260.0002667330.0075136563.57					
GO:00069	to	stressBiolo	process1272440570145861.331911130.0002833570.0079210293.547665433W						
GO:00718	component	disassembl	at	cellular	levelBiolog	process21231570145862.3263157890.00029			
GO:00439	complex	subunit	organizatio	process57913570145861.5975903610.0002958240.008145183.528					
GO:00517	processBio	process55873570145861.6121661540.000302810.0082753113.518829198B2M//PPBP//IFI							
GO:00718	component	assembly	at	cellular	levelBiolog	process53833570145861.6281417830.00030			
GO:00512	regulation	of	protein	metabolic	processBio	process29371570145862.0002553550.00031			
GO:00434	exchangeB	process625570145866.1414736840.0003253360.0086331933.487667798CENPA//OIP5//C							
GO:00709	K11-linked	ubiquitinati	process625570145866.1414736840.0003253360.0086331933.487667798UBE2						
GO:00224	component	disassemb	process21233570145862.3063474140.0003283810.0086513023.483622059HM						
GO:00314	regulation	of	protein	modificatio	processBio	process24285570145862.1549030470.00035			
GO:00226	component	assemblyB	process671130570145861.517251980.0003750620.009740983.425896561RPS						
GO:00513	of	spindle	microtubule	to	chromosom	process4105701458610.235789470.0004014			
GO:00314	regulation	of	protein	modificatio	processBio	process16157570145862.6078444520.00043			
GO:00322	of	cellular	protein	metabolic	processBio	process57927570145861.5734627830.00043			
GO:00516	of	organelle	localization	process1185570145863.3115789470.0004563970.0115263793.3406					

GO:00064(	modificatio	processBio	process1112107570145861.3480928230.0004836740.0121316033.315447281(					
GO:00335!	response	to	stressBiolo	process53850570145861.5955789470.0004983890.0124156553.30;				
GO:00709;	assemblyB	process962570145863.7146010190.0006368330.0157573083.1959746RPS14//RPS15//WI						
GO:00070!	metaphase	transitionBi	process850570145864.0943157890.0006521370.0160044663.185661065CENI					
GO:00500(	localization	process519570145866.7340720220.0006599330.0160044663.180500204DLGAP5//KIF18/						
GO:00513(	of	chromoson	localization	process519570145866.7340720220.0006599330.0160044663.1805(				
GO:00507!	of	catalytic	activityBiol	process641095570145861.4956404710.0007409440.0178508943.1:				
GO:00709:	K48-linked	ubiquitinati	process629570145865.2943738660.0007640250.0182866653.116892389MAR(					
GO:00313!	of	protein	modificatio	processBio	process46724570145861.6258505380.0007917340.018{			
GO:00002(	polyubiquit	process1077570145863.3233082710.0007973190.0188045413.098367971UBE2S//UBE2T						
GO:00650(	complex	assemblyB	process48765570145861.6056140350.0008010670.0188045413.096331004RP					
GO:00109	of	cell	deathBiolo	process601016570145861.5111893910.0008462710.0197391343.07				
GO:00485:	developme	process520570145866.3973684210.0008518710.0197439963.069626202CRKL//CTNNB1/						
GO:00606(	subdivision	of	an	epithelial	terminal	unitBiologi	process412570145868.5298245	
GO:00062(	DNA	replicationE	process1192570145863.0596109840.0008994150.020585353.046040112RNAS					
GO:00302(	nuclear	changeBiol	process630570145865.1178947370.0009238820.0210140093.034383557HMGI					
GO:00300	of	mitotic	metaphase	transitionBi	process741570145864.3689345310.0009482970.02143(			
GO:00705(	of	microtubul	cytoskeleto	organizatio	process853570145863.8625620660.0009716380.02167!			
GO:00305(	signaling	pathwayBi	process1079570145863.2391738840.0009766820.0216790653.010246788FOX					
GO:00003	splicing,	via	transesterif	reactionsBi	process18202570145862.280250130.0009768010.02167			
GO:00313:	regulation	of	cellular	metabolic	processBio	process731301570145861.4358428740.0009		
GO:00650(	of	molecular	functionBio	process751347570145861.4248036570.0010370310.0227401642.9				
GO:00511	importBiolo	process15155570145862.4764006790.0011046250.024014862.956785007IFNG//KPNA2//I						
GO:00070!	cell	cycle	spindle	assembly	checkpoint	process631570145864.9528013580.001108		
GO:00440!	regulation	of	molecular	functionBio	process47757570145861.5887784190.0011296110.0243			
GO:00442:	metabolic	processBio	process3487992570145861.1142563620.0012049050.0258032842.919047246I					
GO:00512!	organizatio	process743570145864.1657282740.0012706280.0270525562.89598159NPM1//KIF11//SG(						
GO:00458	regulation	of	mitotic	metaphase	transitionBi	process632570145864.7980263160.0013196		
GO:00429{	of	apoptosisB	process58993570145861.4946520380.0013373490.0281458172.873755239B2I					
GO:00603:	I	interferon-r	signaling	pathwayBi	process969570145863.3377574370.0013971380.028992			
GO:00713!	response	to	type	I	interferonB	process969570145863.3377574370.0013971		
GO:00345(	localization	to	nucleusBio	process16175570145862.3396090230.001401340.0289926892.853				
GO:00343	to	type	I	interferonB	process970570145863.2900751880.0015489680.03186(			
GO:00444	interaction	between	organismsI	process26354570145861.8794528690.0015658170.0320336462.80!				
GO:00430(	of	programme	cell	deathBiolo	process581003570145861.4797502230.0016847420.034			
GO:00069:	component	disassembl	involved	in	apoptosisB	process971570145863.2437361010.0017137		
GO:00062(	replicationE	process20248570145862.0636672330.0017893960.0360042132.747293629TOP2A//RNAS						
GO:00434	modificatio	process1112189570145861.2975932290.0018880020.037410532.723997569CDKN3//CKS						
GO:00003	splicing,	via	transesterif	reactions	with	bulged	adenosine	as nucleophile
GO:00003!	mRNA	splicing,	via	spliceosom	process17197570145862.2082286940.0018899370.0374			
GO:00512	of	protein	metabolic	processBio	process57988570145861.4763157890.0019440280.0382			
GO:00066(	targetingBi	process22286570145861.9684210530.0019755940.0386878432.704302212IFNG//KPNA2/						
GO:00170:	importBiolo	process17198570145862.1970760230.0019947210.0388114282.700117786IFNG//KPNA2/						
GO:00485:	regulation	of	cellular	processBio	process1212424570145861.2773623410.0020031020.03			
GO:00007:	catabolic	process,	endonucle	process524570145865.3311403510.0020523220.0389409432.6877!				
GO:00063(	fragmentat	involved	in	apoptotic	nuclear	changeBiol	process524570145865.3311403	
GO:00458	regulation	of	mitosisBiol	process524570145865.3311403510.0020523220.0389409432.6877!				
GO:00517{	regulation	of	nuclear	divisionBiol	process524570145865.3311403510.0020523220.038940			
GO:00442(	macromole	metabolic	processBio	process2716072570145861.1420861940.0020794190.0392517062.(				
GO:00305	of	BMP	signaling	pathwayBi	process747570145863.8111982080.0021676940.040500			
GO:00310:	organizing	center	organizatio	process747570145863.8111982080.0021676940.0405004892.6640(				
GO:00328{	of	microtubul	processBio	process860570145863.4119298250.0022092670.0406736382.6557!				
GO:00484{	nervous	system	developme	process415570145866.8238596490.0022324960.0406736382.6512(				
GO:00484{	nervous	system	developme	process415570145866.8238596490.0022324960.0406736382.6512(				

GO:00714	response	to	oxygen	levelsBiolo	process415570145866.8238596490.0022324960.04067		
GO:00714	response	to	hypoxiaBio	process415570145866.8238596490.0022324960.0406736382.6512			
GO:00081	localization	process641146570145861.4290805550.0022819520.0413688552.641693475IFNG//KPNA					
GO:00330	localization	process751388570145861.3827165180.0023234310.0419133182.633870261IFNG//VPS13					
GO:00422	nucleoside	metabolic	processBio	process636570145864.2649122810.0024852720.0439665022.6046			
GO:00458	regulation	of	mitosisBiol	process636570145864.2649122810.0024852720.0439665022.6046			
GO:00461	ribonucleos	metabolic	processBio	process636570145864.2649122810.0024852720.0439665022.6046			
GO:00517	regulation	of	nuclear	divisionBiol	process636570145864.2649122810.0024852720.043966		
GO:00063	remodeling	process975570145863.0707368420.0025186080.0443420312.598839419BAZ1B//CENPA//					
GO:00066	import	into	nucleusBio	process14152570145862.3569252080.002542840.0445544522.594			
GO:00229	electron	transport	chainBiolo	process11105570145862.6808020050.0026458510.0461385972.57			
GO:00217	cell	developme	process637570145864.1496443810.0028700850.0496102842.542105168CD9//				
GO:00104	from	mitosisBiol	process416570145866.3973684210.0028855790.0496102842.53976702KNTC1				
GO:00422	small	subunit	biogenesis	process416570145866.3973684210.0028855790.0496102842.5397			
GO:00098	regulation	of	metabolic	processBio	process731357570145861.3765892250.0029763670.050		
GO:00070	cycleBiolog	process526570145864.9210526320.0029781080.0504899642.52605953KIF11//NPM1//CTN					
GO:00319	signaling	cascadeBi	process526570145864.9210526320.0029781080.0504899642.52605953AKT1S				
GO:00430	regulation	of	catalytic	activityBiol	process40651570145861.5723178920.0031169760.0526		
GO:00106	of	cell	communica	process56989570145861.4489489650.0031878960.0535508122.49			
GO:00025	degranulati	process980570145862.8788157890.0039169540.0654971872.407051577EGF//IGF1//ITGA					
GO:00333	localization	to	organelleB	process21285570145861.8855401660.0040997150.0682416212.38			
GO:00083	splicingBio	process22305570145861.8457981020.0043163150.0715219272.364886856SRSF10//SMN					
GO:00510	transportBi	process11112570145862.513251880.00436670.0720308782.359846655NXF1//SRSF10//N					
GO:00098	regulation	of	metabolic	processBio	process591070570145861.4110083620.0044404540.072		
GO:00106	regulation	of	macromole	metabolic	processBio	process561005570145861.4258811210.0044	
GO:00091	diphosphat	metabolic	processBio	process418570145865.6865497080.0045598230.0742136462.3410			
GO:00064	process28423570145861.693865870.0046779980.0758001262.329939975EIF1AX//EIF4H//RPL27A//R						
GO:00150	transportBi	process53943570145861.4382206840.0047391940.0764534252.324295535IFNG//KPNA2/					
GO:00195	metabolic	processBio	process1563317570145861.2034844420.0047732070.0766644072.32118971				
GO:00064	export	from	nucleusBio	process868570145863.0105263160.0048643480.0776862362.3129			
GO:00451	of	protein	localization	process54966570145861.4304674730.0048792560.0776862362.31			
GO:00226	complex	biogenesis	process16199570145862.057445120.0050808640.0805459922.294062424BRI				
GO:00109	regulation	of	cell	cycle	processBio	process869570145862.96689550.005319064	
GO:00000	transition	of	mitotic	cell	cycleBiolog	process11115570145862.4476887870.00533	
GO:00324	regulation	of	type	I	interferon	productionI	process530570145864.26491228
GO:00469	transportBi	process46802570145861.4677254230.0057803210.089606352.238048064RPS15//IFNG//H					
GO:00062	ligationBiol	process310570145867.6768421050.0057992090.089606352.236631206HMGB2//RAD51//					
GO:00400	of	mitotic	spindle	localization	process310570145867.6768421050.0057992090.089606		
GO:00423	of	protein	import	into	nucleusBio	process985570145862.7094736840.0058601	
GO:00468	of	nucleocyto	transportBi	process10101570145862.5336112560.0060774550.0931198322.216			
GO:00016	involved	in	ureteric	bud	morphoger	process643570145863.5706242350.0061791	
GO:00226	complex	assemblyB	process986570145862.6779681760.0063251220.0961103552.198931116RPS1				
GO:00003	snRNP	assemblyB	process531570145864.1273344650.0065525360.0991544892.183590593SRSF				
GO:00105	of	cellular	ketone	metabolic	processBio	process12135570145862.2746198830.00663	
GO:00018	developme	process11119570145862.3654135340.0068783090.102809672.162518289CTNNB1//IFNG/					
GO:00516	localization	process11119570145862.3654135340.0068783090.102809672.162518289RPS15//NDC80/					
GO:00431	of	I-kappaB	kinase/NF-	cascadeBi	process12137570145862.2414137530.0074415260.1107		
GO:00070	organizatio	process34561570145861.5508771930.0075664660.1121797512.121106916PRC1//KIF23//					
GO:00606	bud	morphoger	process645570145863.4119298250.0077320470.1129913182.111705519CTNN				
GO:00424	nucleoside	biosyntheti	processBio	process311570145866.9789473680.0077446260.1129913182.1109			
GO:00424	biosyntheti	processBio	process311570145866.9789473680.0077446260.1129913182.110999544PRTF				
GO:00461	ribonucleos	biosyntheti	processBio	process311570145866.9789473680.0077446260.1129913182.1109			
GO:00009	process759570145863.0360392510.0078707840.1134756392.103981987NUSAP1//KIF20A//BIRC5//A						
GO:00064	export	from	nucleusBio	process759570145863.0360392510.0078707840.1134756392.1039			

GO:000911	metabolic	processBio	process759570145863.0360392510.0078707840.1134756392.103981987PRTF						
GO:007184	component	biogenesis	at	cellular	levelBiolog	process16209570145861.959002770.008045			
GO:004578	regulation	of	cell	cycleBiolog	process874570145862.7664295870.0080965380.115818				
GO:003132	regulation	of	cellular	metabolic	processBio	process54994570145861.3901726150.00852			
GO:00720(	system	developme	process11123570145862.2884895160.0087521130.1242257242.057887098DC						
GO:00453	respirationI	process12140570145862.1933834590.0087873020.1242436332.056144433IDH3A//COX6/							
GO:000632	organizatio	process29465570145861.595902660.0090363890.1268373882.044005086CHD1//BAZ1B//							
GO:00082	of	blood	pressureBi	process10107570145862.3915395970.0090400210.1268373882.043					
GO:007182	complex	subunit	organizatio	process991570145862.5308270680.0090889310.1270368912.0414					
GO:004687	secretionBi	process14176570145862.0355263160.0092505530.127716862.033832321YWHAZ//SYT9/							
GO:005065	acid	transportBi	process11124570145862.2700339560.0092770850.127716862.032588469HNR						
GO:005065	transportBi	process11124570145862.2700339560.0092770850.127716862.032588469HNRNPA1//NXF							
GO:00512	of	RNA	localization	process11124570145862.2700339560.0092770850.127716862.0325					
GO:00322(	regulation	of	cellular	protein	metabolic	processBio	process17231570145861.883208(		
GO:000165	system	developme	process13159570145862.0922211190.0095751980.1297046862.018852259DC						
GO:00070	depolymeri	process422570145864.6526315790.0096339910.1297046862.016193771SBDS//CCNB1//S							
GO:003445	anchoringE	process422570145864.6526315790.0096339910.1297046862.016193771NDC80//SGOL1/							
GO:004884	extension	involved	in	axon	guidanceBi	process422570145864.6526315790.0096339			
GO:005068	of	mRNA	processing	process422570145864.6526315790.0096339910.1297046862.0161					
GO:00106(	regulation	of	macromole	metabolic	processBio	process661268570145861.3319442140.0097			
GO:00063	recombinat	process534570145863.7631578950.00975040.130300862.010977548PCNA//POLE2//RFC							
GO:00229(	transport	chainBiolog	process12142570145862.1624907340.0097850180.130300862.009438364COX						
GO:00070	of	exit	from	mitosisBiol	process312570145866.3973684210.0100298590.131646				
GO:00431	regulation	of	I-kappaB	kinase/NF-	cascadeBio	process312570145866.3973684210.0100298			
GO:00512	of	spindle	localization	process312570145866.3973684210.0100298590.1316463871.9987(					
GO:005165	localization	process312570145866.3973684210.0100298590.1316463871.998705192NDC80//CENPA/							
GO:00072	kinase/NF-	cascadeBio	process14178570145862.0126552340.0101618250.1329021531.993028293NL						
GO:00650(	of	biological	qualityBiol	process1062192570145861.2374471760.0102005520.1329338871.					
GO:00341(	homeostas	process762570145862.8891341260.0102685810.1333459011.988489562INHBA//RPS14//S							
GO:00064(	localization	process11127570145862.2164111070.0109986390.1420792841.958661051HNRNPA1//NX							
GO:00002	assemblyB	process535570145863.6556390980.011018710.1420792841.95786924SRSF10//SRSF9//S							
GO:00901(	regulation	of	transmemb	receptor	protein	serine/thre	kinase	signaling	pathwayBio
GO:00061	nucleoside	nucleotide	and	nucleic	acid	metabolic	processBio	process211474557014	
GO:00159	nucleoside	nucleotide	and	nucleic	acid	transportBi	process12146570145862.103244		
GO:00063	recombinat	process13164570145862.028433890.0122128510.1552897881.913182956KPNA2//TOP2A							
GO:00486	process	involved	in	reproductio	process23355570145861.6579095630.0124489860.1577				
GO:00440(	of	endocrine	processBio	process313570145865.9052631580.0126654490.1595938261.8973					
GO:00102	to	organic	nitrogenBio	process996570145862.3990131580.0126820870.1595938261.8968(					
GO:00070(	chromatid	cohesionBi	process424570145864.2649122810.0131602570.1650440411.880735639BUB1						
GO:00099	transportBi	process14184570145861.9470251720.013322980.166514521.875398609YWHAZ//SYT9//I							
GO:00512	regulation	of	protein	metabolic	processBio	process17241570145861.8050666080.01384			
GO:00063	processing	process24379570145861.6204416050.014017050.1727234591.853343386SRSF10//SMN2							
GO:00462(	oxide	metabolic	processBio	process651570145863.0105263160.0140555960.1727234591.8521					
GO:00468	regulation	of	nucleocyto	transportBi	process651570145863.0105263160.0140555960.172723				
GO:00725	oxygen	species	metabolic	processBio	process651570145863.0105263160.0140555960.172723				
GO:00331	of	intracellula	protein	transportBi	process998570145862.3500537060.0143815150.176137				
GO:00485	regulation	of	biological	processBio	process1252673570145861.1966645010.0147964160.18				
GO:00062	repairBiolo	process22341570145861.6509337860.014955690.1819526131.825193548GTF2H3//RAD5							
GO:00603	of	cellular	localization	process25402570145861.5913851790.0151551260.1836295571.81					
GO:00075(	implantatio	process425570145864.0943157890.0151938160.1836295571.818333124IL1B//SOD1//STC							
GO:00060	process652570145862.9526315790.015380320.1852721491.813034617ARPP19//SDS//ALDOC//GAP								
GO:00327(	of	monooxyge	activityBiol	process538570145863.3670360110.0154872250.1859482531.8100					
GO:00422	assemblyB	process314570145865.4834586470.015659050.1867864491.805234599RPS14//RPS15//N							
GO:00425	anion	generationI	process314570145865.4834586470.015659050.1867864491.805234599NCF2//						

GO:00436	protein	modificatio	process13170570145861.9568421050.0160821480.1912104681.793655959DP						
GO:00346	protein	localization	process33571570145861.4789012810.016158930.1915016221.791587403IFN						
GO:00323	of	intracellula	transportBi	process10117570145862.1871345030.0162836320.1917384611.78					
GO:00900	of	transmemb	receptor	protein	serine/thre	kinase	signaling	pathwayBi	process101
GO:00091	metabolic	processBio	process884570145862.4370927320.0167283730.1963439121.776546305PRTF						
GO:00707	macromole	localization	process33573570145861.4737393220.01691980.1974402371.771604767IFNG						
GO:00067	metabolic	processBio	process671327570145861.2920080910.0172440350.1974402371.763361108C						
GO:00067	metabolic	processBio	process671327570145861.2920080910.0172440350.1974402371.763361108C						
GO:00017	crest	cell	migrationB	process426570145863.9368421050.0174148540.1974402371.7590					
GO:00066	export	from	nucleusBio	process426570145863.9368421050.0174148540.1974402371.7590					
GO:00106	regulation	of	cellular	carbohydra	metabolic	processBio	process426570145863.93684210		
GO:00303	differentiati	process426570145863.9368421050.0174148540.1974402371.759080154FOXD4L1//FOXD							
GO:00325	to	progestero	stimulusBi	process426570145863.9368421050.0174148540.1974402371.7590					
GO:00454	regulation	of	nitric	oxide	biosyntheti	processBio	process426570145863.93684210		
GO:00459	regulation	of	carbohydra	metabolic	processBio	process426570145863.9368421050.0174148			
GO:00509	cell	differentiati	process426570145863.9368421050.0174148540.1974402371.759080154FOXD						
GO:00424	to	drugBiolog	process19288570145861.6881944440.018419110.2081814251.734731353CEN						
GO:00099	regulation	of	signal	transductio	process28472570145861.5180196250.0187613110.2113				
GO:00511	process1643640570145861.1529323310.0190050510.2116579011.721130955RPS15//IFNG//NDC80//								
GO:00327	regulation	of	monooxyg	activityBiol	process315570145865.1178947370.0190156880.211657				
GO:00513	plate	congressio	process315570145865.1178947370.0190156880.2116579011.720887957KIF18						
GO:00519	of	chromoson	segregatio	process315570145865.1178947370.0190156880.2116579011.7208					
GO:00106	regulation	of	cell	communica	process29495570145861.4991812870.0197224240.218				
GO:00427	synthesis	coupled	electron	transportBi	process655570145862.7915789470.0198680320.21914				
GO:00427	ATP	synthesis	coupled	electron	transportBi	process655570145862.7915789470.0198680			
GO:00346	macromole	complex	disassemb	process12157570145861.9558833390.0203217160.2234778531.69					
GO:00311	polymeriza	or	depolymeri	process541570145863.1206675220.0210261450.2305321681.677					
GO:00230	regulation	of	signalingBi	process29498570145861.4901500740.021190340.2316388831.673					
GO:00323	regulation	of	intracellula	transportBi	process656570145862.7417293230.0215425660.23339				
GO:00456	of	osteoblast	differentiati	process656570145862.7417293230.0215425660.2333990381.6667					
GO:00513	of	oxidoreduc	activityBiol	process656570145862.7417293230.0215425660.2333990381.6667					
GO:00603	signaling	pathwayBi	process772570145862.4878654970.0220894560.2384700391.655814976IFNG						
GO:00329	complex	disassemb	process12159570145861.9312810330.0221960950.2384700391.653723417BA						
GO:00512	of	localization	process1423121570145861.1642759570.0222706170.2384700391.652267746						
GO:00068	protein	transportBi	process27458570145861.5085497590.0222710960.2384700391.652258412IFN						
GO:00510	of	DNA	metabolic	processBio	process11141570145861.9963419190.0224361850.2388				
GO:00068	metabolic	processBio	process428570145863.6556390980.022437230.2388521381.649030764SOD1/						
GO:00609	hormone	secretionBi	process316570145864.7980263160.0227380210.2413525571.64324734INHBA						
GO:00343	to	interferon-	process889570145862.300177410.0229230640.2426134661.639727339HLA-B						
GO:00302	differentiati	process657570145862.6936288090.0233098980.2452897871.63245963HMGB2//INHBA//J							
GO:00512	regulation	of	protein	transportBi	process657570145862.6936288090.0233098980.24528				
GO:00071	process10124570145862.0636672330.0234537350.245393081.629787984TRIP13//TOP2A//SYCP2//								
GO:00513	phase	of	meiotic	cell	cycleBiolo	process10124570145862.0636672330.02345			
GO:00512	regulation	of	multicellula	organismal	processBio	process20316570145861.6195869420.02363			
GO:00488	of	number	of	cellsBiolog	process9107570145862.1523856370.0241576770.25132				
GO:00718	complex	subunit	organizatio	process37676570145861.4006072870.0242526850.2515958461.61					
GO:00328	of	homeostati	processBio	process11143570145861.9684210530.0245970590.2541608771.60					
GO:00106	regulation	of	organelle	organizatio	process10125570145862.0471578950.0246387520.2541				
GO:00423	of	NF-kappaE	import	into	nucleusBio	process429570145863.5295825770.0252459			
GO:00423	import	into	nucleusBio	process429570145863.5295825770.0252459590.257914331.59780					
GO:00484	nervous	system	developme	process429570145863.5295825770.0252459590.257914331.59780					
GO:00061	electron	transport,	NADH	to	ubiquinone	process543570145862.9755201960.0253547			
GO:00068	oxide	biosyntheti	processBio	process543570145862.9755201960.0253547680.257914331.59594					
GO:00513	cell	cycleBiolo	process10126570145862.030910610.0258662610.2623884961.587266348TRI						

GO:00071	receptor	protein	serine/thre	kinase	signaling	pathwayBio	process13182570145861.827819
GO:00343	localization	at	cell	surfaceBiol	process317570145864.5157894740.0268265650.26773		
GO:00354	import	into	nucleusBio	process317570145864.5157894740.0268265650.2677385351.5714			
GO:00354	of	catenin	import	into	nucleusBio	process317570145864.5157894740.0268265	
GO:00518	of	mitochondr	membrane	potentialBio	process317570145864.5157894740.0268265650.26773		
GO:00436	protein	complex	disassemb	process11145570145861.9412704170.0269054560.2677385351.57			
GO:00468	of	hormone	secretionBi	process11145570145861.9412704170.0269054560.2677385351.57			
GO:00420	healingBiol	process32574570145861.4265908670.0276843770.2743299841.557765243EGF//ZFPM2//					
GO:00423	regulation	of	protein	import	into	nucleusBio	process544570145862.9078947
GO:00070	organizatio	process11146570145861.9279740450.0281165260.2766764721.551038345IFI6//SOD2//RN					
GO:00001	mRNA	catabolic	process,	nonsense-r	decayBiolo	process430570145863.4119298250.028256	
GO:00215	nerve	developme	process430570145863.4119298250.0282569640.2766764721.548874508FOXD				
GO:00456	regulation	of	osteoblast	differentiati	process430570145863.4119298250.0282569640.27667		
GO:00432	complex	disassemb	process11147570145861.9148585750.0293663140.2867718481.532150557RP				
GO:00422	biogenesis	process10129570145861.9836801310.0298110310.2903404111.525623008RPS14//RPS1					
GO:00015	follicle	developme	process545570145862.8432748540.0302158690.292726221.519764915CCNE				
GO:00346	response	to	reactive	oxygen	speciesBio	process545570145862.8432748540.030215	
GO:00062	repair,	DNA	gap	fillingBiolog	process318570145864.2649122810.0312799130.29935		
GO:00159	nucleoside	and	nucleotide	interconver	process318570145864.2649122810.0312799130.29935		
GO:00305	genitalia	developme	process318570145864.2649122810.0312799130.2993559861.504734458SYCF				
GO:00466	regulation	of	insulin	receptor	signaling	pathwayBio	process318570145864.2649122
GO:00193	biosyntheti	processBio	process661570145862.5169974120.0313442450.2993559861.503842191SDS//				
GO:00423	modified	amino	acid	biosyntheti	processBio	process431570145863.3018675720.031472	
GO:00424	biogenic	amine	biosyntheti	processBio	process431570145863.3018675720.0314724340.29935		
GO:00422	to	chemical	stimulusBio	process1012172570145861.1899340890.0324989730.3077242111.			
GO:00713	response	to	interferon-g	process778570145862.2964912280.0325202810.3077242111.4878			
GO:00347	regulation	of	transmemb	transportBi	process546570145862.7814645310.0328512590.30852		
GO:00427	ubiquitinati	involved	in	ubiquitin-de	protein	catabolic	processBio process546570145862
GO:00512	developme	process9113570145862.0380996740.0328573280.3085218811.483367762IMPAD1//NPPC/					
GO:00022	to	molecule	of	bacterial	originBiolog	process11150570145861.8765614040.03335	
GO:00061	phosphoryl	process662570145862.4764006790.0336023510.3131089341.47363033NDUFA8//NDUFB					
GO:00313	regulation	of	cellular	catabolic	processBio	process662570145862.4764006790.0336023	
GO:00430	regulation	of	catalytic	activityBiolo	process23391570145861.5052631580.033782450.3139		
GO:00300	secretionBi	process10132570145861.9385964910.0341638150.3167288391.466433635SYT9//IFNG//I					
GO:00355	signal	transductio	process801680570145861.2185463660.0344712610.3187721151.46254283CA				
GO:00468	regulation	of	nucleocyto	transportBi	process432570145863.1986842110.0348939830.32186		
GO:00346	nitrogen	compound	metabolic	processBio	process2225149570145861.1032944570.0355296150.32		
GO:00074	nervous	system	developme	process547570145862.7222844340.0356254780.3261512551.4482			
GO:00324	of	type	I	interferon	productionI	process547570145862.7222844340.035625	
GO:00302	of	ossification	process897570145862.1104720560.0359287890.3262575711.444557422SMUF				
GO:00000	of	transcriptio	involved	in	G1/S	phase	of mitotic cell
GO:00435	process319570145864.0404432130.0360949360.3262575711.442553725CRH//ADNP//AKT1S1						
GO:00603	of	response	to	interferon-g	process319570145864.0404432130.0360949360.32625		
GO:00603	of	interferon-g	signaling	pathwayBio	process319570145864.0404432130.0360949360.32625		
GO:00903	acid	metabolic	processBio	process1764007570145861.1239698950.0362192640.3262575711.			
GO:00160	reproductio	process24415570145861.4798731770.036260740.3262575711.440563332EIF2AK2//RPL2					
GO:00075	coagulation	process27481570145861.4364153630.0379245810.3403917061.421079207TFPI//EGF//IG					
GO:00063	catabolic	processBio	process664570145862.3990131580.0384278980.3440659231.415353371GTF2				
GO:00339	to	lipidBiologi	process548570145862.6655701750.0385399270.3442273521.414089107TGFE				
GO:00510	regulation	of	secretionBi	process781570145862.2114359970.0387949450.3456620181.4112			
GO:00431	metabolic	processBio	process2826677570145861.080759560.0389903210.3465596041.409043185K				
GO:00071	communica	process811715570145861.2085990490.0394355030.3496678281.404112613WIPI2//ATG1					
GO:00082	regulation	of	cell	proliferatio	process23398570145861.478788680.0400327030.3541		
GO:00075	process27484570145861.4275119620.0404587190.3561534371.39298787EGF//ZFPM2//GGCX//GRB						

GO:00508	process27484570145861.4275119620.0404587190.3561534371.39298787EGF//ZFPM2//GGCX//GRB						
GO:00323	to	estradiol	stimulusBio	process782570145862.1844672660.0410507280.3604982411.3866			
GO:00320	of	TOR	signaling	cascadeBio	process320570145863.8384210530.0412669650.360667		
GO:00420	regulation	of	cytokine	biosyntheti	processBio	process320570145863.8384210530.0412669	
GO:00903	acid	phosphodie	bond	hydrolysisB	process549570145862.6111707840.0415957630.362675		
GO:00461	biosyntheti	processBio	process8100570145862.0471578950.0418774560.3642642411.378019704SDS				
GO:00100	to	organic	substanceB	process591206570145861.2518896740.0420802640.3650994371.3			
GO:00425	of	tyrosine	phosphoryl	of	STAT	proteinBiol	process434570145863.01052631
GO:00903	regulation	of	intracellula	protein	transportBi	process434570145863.0105263160.0423589	
GO:00324	to	lipopolysac	process10137570145861.8678447940.0423722720.3650994371.372918247MT				
GO:00650	regulationB	process3327972570145861.0656930840.0430351260.3691710881.366176921KPNA2//UBI					
GO:00511	regulation	of	nitrogen	compound	metabolic	processBio	process48955570145861.28617
GO:00461	catabolic	processBio	process8101570145862.0268890050.0439969840.3764414881.35657709ALDC				
GO:00067	compound	metabolic	processBio	process10138570145861.8543096870.044162270.3769749021.354			
GO:00508	of	body	fluid	levelsBiolo	process30554570145861.3857115710.0446026990.3798		
GO:00163	process12177570145861.7348795720.0451812620.3838834821.345041644PPM1K//FBXW11//PPM1A						
GO:00093	biosyntheti	processBio	process9120570145861.9192105260.0454948230.3847622191.342038023CDC				
GO:00182	modificatio	process9120570145861.9192105260.0454948230.3847622191.342038023TPST1//GH1//A					
GO:00100	cell	differentiati	process784570145862.132456140.0458139190.386198351.339002552CTNNB				
GO:00510	regulation	of	bindingBiol	process10139570145861.840969330.0460029310.386198351.3372			
GO:00604	developme	process8102570145862.0070175440.046185990.386198351.335489738FOXD4L1//FOXD4					
GO:00062	DNA	replication	initiationBio	process435570145862.9245112780.046402860.386198351.333455			
GO:00140	crest	cell	developme	process435570145862.9245112780.046402860.386198351.333455			
GO:00311	of	microtubule	polymeriza	or	depolymeri	process435570145862.9245112780.046402	
GO:00454	of	nitric	oxide	biosyntheti	processBio	process435570145862.9245112780.046402	
GO:00064	ADP-ribosy	process321570145863.6556390980.0467899660.3867829651.329847275ADNP//SIRT4//TR					
GO:00071	meiosisBio	process321570145863.6556390980.0467899660.3867829651.329847275UBR2//TRIP13//S					
GO:00109	regulation	of	glucose	metabolic	processBio	process321570145863.6556390980.0467899	
GO:00140	to	amine	stimulusBio	process668570145862.2578947370.0493464140.4069967751.3067			

ID

s)/(List.Total/Pop.Tot

thod
e))

Enrichmen GENES

1//KIF23//NDC80//ZWINT//MAD2L1//NEK2//NUSAP1//CDKN3//ORC6//INHBA//PCNA//POLE2//MCM10//PSMA3//PS
C1//KIF23//NDC80//ZWINT//MAD2L1//NEK2//NUSAP1//KPNA2//UBE2C//PSMA8//ORC6//MDM2//ANAPC11//MCM1
3//NDC80//ZWINT//MAD2L1//NEK2//NUSAP1//KPNA2//UBE2C//PSMA8//ORC6//MDM2//ANAPC11//MCM10//PSMA
C1//KIF23//NDC80//ZWINT//MAD2L1//NEK2//NUSAP1//KPNA2//CDKN3//ORC6//INHBA//PCNA//POLE2//MCM10//P
NDC80//ZWINT//MAD2L1//NEK2//NUSAP1//KPNA2//KIF20A//CENPA//CENPF//STAG2//SGOL1//CENPI//BIRC5//SK
3934184UBE2C//PSMA8//ORC6//MAD2L1//MDM2//ANAPC11//MCM10//PSMA3//PSMA6//PSMA7//PSMB5//PSMC5
E-1012.03614131UBE2C//PSMA8//ORC6//MAD2L1//MDM2//ANAPC11//MCM10//PSMA3//PSMA6//PSMA7//PSMB
E-124.68159E-1011.99023466NDC80//ZWINT//MAD2L1//NEK2//NUSAP1//SKA2//CENPA//CENPF//STAG2//SGOL1
0A//BIRC5//AURKC//PRC1//KIF23//KLHL21//TOP1//IL1B//PPBP//VEGFA//SYCP2//NDC80//USP16//CENPF//STAG2
27E-0911.39161985PSMA8//MDM2//TRIAP1//PSMA3//PSMA6//PSMA7//PSMB5//PSMC5//PSMD9//PSMD11//PSMD
T//MAD2L1//NEK2//NUSAP1//SKA2//CENPA//CENPF//STAG2//SGOL1//CENPI//BIRC5//CASC5//SPC25//CENPH//B
/NEK2//NUSAP1//SKA2//CENPA//CENPF//STAG2//SGOL1//CENPI//BIRC5//CASC5//SPC25//CENPH//BUB1//KIF18
AFX//PSMA8//MDM2//TRIAP1//PSMA3//PSMA6//PSMA7//PSMB5//PSMC5//PSMD9//PSMD11//PSMD12//PSME1//Z
/MAD2L1//NEK2//NUSAP1//SKA2//CENPA//CENPF//STAG2//SGOL1//CENPI//BIRC5//CASC5//SPC25//CENPH//BU
5E-0910.40531459UBE2C//PSMA8//ORC6//MAD2L1//MDM2//ANAPC11//MCM10//PSMA3//PSMA6//PSMA7//PSMB
E2C//PSMA8//ORC6//MAD2L1//MDM2//ANAPC11//MCM10//PSMA3//PSMA6//PSMA7//PSMB5//PSMC5//PSMD9//P
2286495PSMA8//MDM2//TRIAP1//PSMA3//PSMA6//PSMA7//PSMB5//PSMC5//PSMD9//PSMD11//PSMD12//PSME1
59E-089.851535436UBE2C//PSMA8//ORC6//MAD2L1//MDM2//ANAPC11//MCM10//PSMA3//PSMA6//PSMA7//PSM
41.96952E-093.79599E-078.705640188MAD2L1//UBE2C//PSMA8//UBE2S//ANAPC11//PSMA3//PSMA6//PSMA7//P
T//MAD2L1//NEK2//NUSAP1//TOP2A//STAG2//RAD51C//BUB1//DLGAP5//KIF18A//CCNB1//SGOL1//CASC5//CENP
E-083.64538E-067.679756553PSMA8//MDM2//TRIAP1//PSMA3//PSMA6//PSMA7//PSMB5//PSMC5//PSMD9//PSMD
582832GCLM//UBE2C//MAD2L1//ANAPC11//PSMA3//PSMA6//PSMA7//PSMB5//PSMC5//PSMD9//PSMD11//PSMD
5E-067.558068946CDKN3//ORC6//INHBA//PCNA//POLE2//MCM10//PSMA3//PSMA6//PSMA7//PSMB5//PSMC5//PS
//CENPF//STAG2//ZWINT//SGOL1//CENPI//BIRC5//SKA2//MAD2L1//CASC5//SPC25//CENPH//BUB1//KIF18A//CCN
5705264DCP2//NCBP2//UPF3B//EIF4A3//ISG15//MDM2//UBR5//HERC6//SMURF1//SMURF2//LSM3//GTF2H3//HMG
CNA//POLE2//MCM10//PSMA3//PSMA6//PSMA7//PSMB5//PSMC5//PSMD9//PSMD11//PSMD12//PSME1//RRM2//S
E-088.05132E-067.225112504PCNA//RRM2//CCNE1//WNT9A//CDKN3//ORC6//INHBA//POLE2//MCM10//PSMA3//
3//RPS14//RPS15//WIPI2//ATG12//PTTG2//PTTG1//NDC80//ZWINT//MAD2L1//NEK2//NUSAP1//SKA2//CENPA//CC
E-067.147829582UBE2C//MAD2L1//ANAPC11//PSMA3//PSMA6//PSMA7//PSMB5//PSMC5//PSMD9//PSMD11//PS
E-057.039089587GCLM//UBE2C//PSMA8//ANAPC11//PSMA3//PSMA6//PSMA7//PSMB5//PSMC5//PSMD9//PSMD1
9E-071.517E-056.891370676PRC1//KIF23//RPS14//RPS15//WIPI2//ATG12//PTTG2//PTTG1//NDC80//ZWINT//MAD
45861.4665532311.33957E-071.53166E-056.873034485BRIX1//PRC1//KIF23//RPS14//RPS15//WIPI2//ATG12//PTT
DC80//KIF11//STMN1//SPC25//SBDS//CCNB1//HAUS2//UBE2C//ZWINT//CKS2//AURKA
5//SMURF1//UBE2D3//MDM2//HERC6//SMURF2//UBE2W//SUZ12//UBQLN1//UBE2S//UBE2T//UBE2C//MAD2L1//A
KIF23//NDC80//CENPA//STMN1//KIF18A//UBE2C//ZWINT//CKS2//KIF11//AURKA//SPC25//NPM1//SGOL1//CASC5
E-072.23955E-056.657255562FBXW11//UBR5//SMURF1//UBE2D3//MDM2//HERC6//SMURF2//FEM1B//DCAF7//MA
362.4141012912.33745E-072.25996E-056.631257636FBXW11//UBR5//SMURF1//UBE2D3//MDM2//HERC6//SMUR

process1577570145864.9849624062.3594E-072.25996E-056.627198751UBE2C//MAD2L1//ANAPC11//PSMA3//P
6E-056.618553467UBE2C//NPLOC4//PSMA8//UBE2S//MAD2L1//ANAPC11//PSMA3//PSMA6//PSMA7//PSMB5//PS
072.57942E-056.550135515UBE2C//PSMA8//ANAPC11//PSMA3//PSMA6//PSMA7//PSMB5//PSMC5//PSMD9//PSM
170399UBE2C//NPLOC4//PSMA8//UBE2S//MAD2L1//ANAPC11//PSMA3//PSMA6//PSMA7//PSMB5//PSMC5//PSM
006075MDM2//UBR5//HERC6//SMURF1//SMURF2//USP16//UBE2C//PSMA8//CYLD//UBR2//PSMA3//PSMA6//PSI
006075ISG15//MDM2//UBR5//HERC6//SMURF1//SMURF2//USP16//UBE2C//PSMA8//CYLD//UBR2//PSMA3//PSM
cycleBiolog process1471570145865.0458117125.09066E-074.23682E-056.293225897UBE2C//PSMA8//ANAPC11/
673587MDM2//UBR5//HERC6//SMURF1//SMURF2//UBE2C//NPLOC4//PSMA8//UBE2S//MAD2L1//ANAPC11//PSM
5869.83053E-077.82596E-056.007422964ISG15//MDM2//UBR5//HERC6//SMURF1//SMURF2//USP16//UBE2C//PS
KIF23//SBDS//CCNB1//KIF11//NDC80//STMN1//SPC25
//MAD2L1//NEK2//NUSAP1//PCNA//POLE2//RFC5//TOP2A//CHD1//BAZ1B//CENPA//OIP5//CENPI//H2BFM//H2AFX
9516194ISG15//MDM2//UBR5//HERC6//SMURF1//SMURF2//PSMA8//PSMA3//PSMA6//PSMA7//PSMB5//USP16//U
2//NCBP2//UPF3B//EIF4A3//ISG15//MDM2//UBR5//HERC6//SMURF1//SMURF2//LSM3//GTF2H3//HMGB2//CYCS//
3305639SMURF1//UBE2C//MAD2L1//ANAPC11//PSMA3//PSMA6//PSMA7//PSMB5//PSMC5//PSMD9//PSMD11//PS
PRC1//KIF23//RPS14//RPS15//WIPI2//ATG12//PTTG2//PTTG1//NDC80//ZWINT//MAD2L1//NEK2//NUSAP1//SKA2//
in cell cycle arrestBiolo process1261570145865.0339948233.4133E-060.0002083255.46682
233.4133E-060.0002083255.466825313PSMA8//MDM2//TRIAP1//PSMA3//PSMA6//PSMA7//PSMB5//PSMC5//PSM
233.4133E-060.0002083255.466825313PSMA8//MDM2//TRIAP1//PSMA3//PSMA6//PSMA7//PSMB5//PSMC5//PSM
233.4133E-060.0002083255.466825313PSMA8//MDM2//TRIAP1//PSMA3//PSMA6//PSMA7//PSMB5//PSMC5//PSM
5.0339948233.4133E-060.0002083255.466825313PSMA8//MDM2//TRIAP1//PSMA3//PSMA6//PSMA7//PSMB5//PS
233.4133E-060.0002083255.466825313PSMA8//MDM2//TRIAP1//PSMA3//PSMA6//PSMA7//PSMB5//PSMC5//PSM
damage checkpoint process1261570145865.0339948233.4133E-060.0002083255.466825313PSMA8//MDM2//T
1570145865.0339948233.4133E-060.0002083255.466825313PSMA8//MDM2//TRIAP1//PSMA3//PSMA6//PSMA7/
02170365.441856342BRIX1//PRC1//KIF23//RPS14//RPS15//WIPI2//ATG12//PTTG2//PTTG1//NDC80//ZWINT//MAD
569135.361540157UBE2C//PSMA8//ANAPC11//PSMA3//PSMA6//PSMA7//PSMB5//PSMC5//PSMD9//PSMD11//PS
-060.0003977775.164737727PSMA8//MDM2//TRIAP1//PSMA3//PSMA6//PSMA7//PSMB5//PSMC5//PSMD9//PSMD
/MAD2L1//ANAPC11//BUB1//CASC5//CCNB1//DYNC1LI1//BIRC5
5//MDM2//UBR5//HERC6//SMURF1//SMURF2//PSMA8//PSMA3//PSMA6//PSMA7//PSMB5//USP16//UBE2C//CYLD
4768274RAD51//CENPA//OIP5//CENPI//H2BFM//H2AFX//H2AFZ//HMGB2//NAP1L4//NPM1//H2AFJ//CASC5//CENP
KPNA2//NDC80//CENPA//CCDC88A//KIF20A//KIF1B//KIF11//KIF18A//STMN1//UBE2C//ZWINT//CKS2//AURKA//SP
-060.0004904935.034236862PSMA8//MDM2//TRIAP1//PSMA3//PSMA6//PSMA7//PSMB5//PSMC5//PSMD9//PSMD
A8//MDM2//TRIAP1//PSMA3//PSMA6//PSMA7//PSMB5//PSMC5//PSMD9//PSMD11//PSMD12//PSME1//WNT9A
cycleBiolog process1267570145864.5831893179.48825E-060.0004963715.022814032MAD2L1//UBE2C//ANAPC1
-050.0005505224.971685579PSMA8//ORC6//POLE2//MCM10//PSMA3//PSMA6//PSMA7//PSMB5//PSMC5//PSMD
IPI2//ATG12//DCP2//NCBP2//UPF3B//EIF4A3//ISG15//MDM2//UBR5//HERC6//SMURF1//SMURF2//LSM3//GTF2H3
4.5157894741.11184E-050.0005577484.953957189PSMA8//MDM2//TRIAP1//PSMA3//PSMA6//PSMA7//PSMB5//P
PA//OIP5//CENPI//H2BFM//H2AFX//H2AFZ//HMGB2//NAP1L4//NPM1//H2AFJ//CASC5//CENPH//HIST2H2AC//H2A
/HNRNPA1//NXF1//SRSF10//NCBP2//GLE1//UPF3B//U2AF1//SRSF9//KPNA2//IPO11//RPL23//SMURF1//STRADA//
4.1069525671.41335E-050.0006810144.849748802PSMA8//MDM2//TRIAP1//PSMA3//PSMA6//PSMA7//PSMB5//P
/HNRNPA1//NXF1//SRSF10//NCBP2//GLE1//UPF3B//U2AF1//SRSF9//KPNA2//IPO11//RPL23//SMURF1//STRADA//
F//UBE2C//MAD2L1//ANAPC11//BUB1//EGF//IGF1//IL1B//NUSAP1//DLGAP5//DYNC1LI1//NEK2//CDK11A
0664NEK2//CDK11A//CENPF//UBE2C//MAD2L1//ANAPC11//BUB1//EGF//IGF1//IL1B//NUSAP1//DLGAP5//DYNC1L
D51//CENPA//OIP5//CENPI//H2BFM//H2AFX//H2AFZ//HMGB2//NAP1L4//NPM1//H2AFJ//CASC5//CENPH//HIST2H
5624.755277411UBE2C//MAD2L1//ANAPC11//PSMA3//PSMA6//PSMA7//PSMB5//PSMC5//PSMD9//PSMD11//PSM
050.0007845624.755277411UBE2C//MAD2L1//ANAPC11//PSMA3//PSMA6//PSMA7//PSMB5//PSMC5//PSMD9//PS
020054.7404631RPS14//RPS15//SRSF10//SMN2//SNRPD1//SNRPD2//NCBP2//RAD51//CENPA//OIP5//CENPI//H2
/CENPI//H2BFM//H2AFX//H2AFZ//HMGB2//NAP1L4//NPM1//H2AFJ//CASC5//CENPH//HIST2H2AC//H2AFV//BAZ1
/CENPI//H2BFM//H2AFX//H2AFZ//HMGB2//NAP1L4//NPM1//H2AFJ//CASC5//CENPH//HIST2H2AC//H2AFV//NUSA
ENPI//NPM1//CASC5//CENPH//H2BFM//H2AFX//H2AFZ//HMGB2//NAP1L4//H2AFJ//HIST2H2AC//H2AFV
SYCP2//TRIM69//PSMA8//CTNNB1//EYA2//ALDOC//KIF1B//JMJD6//MAGI3//CKAP2//NGFRAP1//MAGEH1//UBQLN
12420884.525083175UBE2C//PSMA8//ORC6//MAD2L1//MDM2//ANAPC11//MCM10//PSMA3//PSMA6//PSMA7//PS
M69//PSMA8//CTNNB1//EYA2//ALDOC//KIF1B//JMJD6//MAGI3//CKAP2//NGFRAP1//MAGEH1//UBQLN1//HMGB2//
323.28155E-050.0013352254.483921514PSMA8//PSMA3//PSMA6//PSMA7//PSMB5//PSMC5//PSMD9//PSMD11//P
-050.0013418464.476974331PSMA8//PSMA3//PSMA6//PSMA7//PSMB5//PSMC5//PSMD9//PSMD11//PSMD12//PS

I6//SOD2//SYCP2//TRIM69//PSMA8//CTNNB1//EYA2//ALDOC//KIF1B//JMJD6//MAGI3//CKAP2//NGFRAP1//MAGEI
400410766CDKN3//CKS2//SERTAD1//BCCIP//CCNE2//EGF//TGFBR1//IL1B//SOD1//SPRED2//FBXW11//UBR5//SM
30//ZWINT//MAD2L1//NEK2//NUSAP1//DLGAP5//KIF18A//CCNB1//TOP2A
P2//NCBP2//UPF3B//EIF4A3//SRSF10//SMN2//SNRPD1//SNRPD2//LSM3//HNRNPM//SYNCRIP//HNRNPA1//HNRI
CS//SOD1//FOXL2//TPD52L1//CFDP1//HSPA1B//BIRC5//IGF1//IL1B//NPM1//TRIAP1//BCL2A1//BNIP1//YWHAZ//FE
AFX//PSMA8//MDM2//TRIAP1//PSMA3//PSMA6//PSMA7//PSMB5//PSMC5//PSMD9//PSMD11//PSMD12//PSME1//N
/CENPI//H2BFM//H2AFX//H2AFZ//HMGB2//NAP1L4//NPM1//H2AFJ//CASC5//CENPH//HIST2H2AC//H2AFV
ZWINT//CKS2//HMGB2//IGF1//MDM2//PCNA//AURKA//TOP2A//AKT1S1
0//UBE2C//ZWINT//CKS2//HMGB2//IGF1//MDM2//PCNA//AURKA//TOP2A//AKT1S1
724.24843359MYC//FOXD4L1//FOXD4//FOXD2//FOXD4L3
A2//PRC1//KIF23//RPS14//RPS15//WIPI2//ATG12//IFNG//CENPF//EGF//ORC6//IGF1//PCNA//POLE2//MCM10//RF
11984.133911194H2AFX//GTF2H3//RAD51AP1//RAD51//STRA13//RDM1//EYA2//UBE2T//NPM1//PCNA//POLE2//E
A//OIP5//CENPI//NPM1//CASC5//CENPH//BAZ1B
A1//NXF1//SRSF10//NCBP2//GLE1//UPF3B//U2AF1//SRSF9//SMURF1//STRADA//TXN//IL1B
96570145863.4652412288.89737E-050.0030713784.050738584PSMA8//MDM2//TRIAP1//PSMA3//PSMA6//PSMA
030713784.047001348UBE2C//PSMA8//ORC6//MAD2L1//MDM2//ANAPC11//MCM10//PSMA3//PSMA6//PSMA7//PS
NA2//CENPF//EGF//ORC6//IGF1//PCNA//POLE2//MCM10//RFC5//RRM2//PAPD5//TOP1//TOP2A//NAE1//GTF2H3//
MA8//MDM2//TRIAP1//PSMA3//PSMA6//PSMA7//PSMB5//PSMC5//PSMD9//PSMD11//PSMD12//PSME1//FEM1B//H
2//DCP2//NCBP2//UPF3B//EIF4A3//ISG15//MDM2//UBR5//HERC6//SMURF1//SMURF2//LSM3//GTF2H3//ALDOC//
/NDC80//CENPA//YWHAZ//EGF//IGF1//ITGA2B//LAMP2//PPBP//SOD1//VEGFA//CALU//CD9//HNRNPA1//NXF1//SF
0940.0046082253.850966627PSMA8//ORC6//PCNA//POLE2//PSMA3//PSMA6//PSMA7//PSMB5//PSMC5//PSMD9
3160.0001425090.0046183013.846157645IL1B//PELI2//GH1//TGFBR1//SBDS//EIF4A3//SMURF1//MDM2//CCNB1/
66755CENPA//OIP5//CENPI//NPM1//CASC5//CENPH
18243.819666755CENPA//OIP5//CENPI//NPM1//CASC5//CENPH
66755CENPA//OIP5//CENPI//NPM1//CASC5//CENPH
234343.812176108UBE2C//MAD2L1//ANAPC11//PSMA3//PSMA6//PSMA7//PSMB5//PSMC5//PSMD9//PSMD11//PS
4928NUSAP1//DLGAP5//KIF18A//CCNB1//NDC80//ZWINT//MAD2L1//NEK2
3040.0001867880.0057300933.72865024PSMA8//MDM2//TRIAP1//PSMA3//PSMA6//PSMA7//PSMB5//PSMC5//PS
57300933.726375396RPS15//IFNG//NDC80//CENPA//YWHAZ//EGF//IGF1//ITGA2B//LAMP2//PPBP//SOD1//VEGFA
PF//UBE2C//MAD2L1//ANAPC11//BUB1//DYNC1LI1//CASC5
49453.715603624CENPF//UBE2C//MAD2L1//ANAPC11//BUB1//DYNC1LI1//CCNB1
660.0057649453.712049482FOXD4L1//FOXD4//FOXD2//FOXD4L3//TWSG1
RIX1//RPS14//RPS15//WIPI2//ATG12//SRSF10//SMN2//SNRPD1//SNRPD2//NCBP2//RAD51//CASC5//CENPA//OIF
9828445CENPA//OIP5//CENPI//H2BFM//H2AFX//H2AFZ//HMGB2//NAP1L4//NPM1//H2AFJ//CASC5//CENPH//HIST
//PCNA//POLE2//PSMA3//PSMA6//PSMA7//PSMB5//PSMC5//PSMD9//PSMD11//PSMD12//PSME1//RFC5//SKP1
7294281BUB1//NEK2//CDK11A//CENPF//UBE2C//MAD2L1//ANAPC11//NPM1//CCL26//CTNNB1//SKA2//STMN1//R
96818CENPA//OIP5//CENPI//NPM1//CASC5//CENPH
96818IFNG//MYC//FOXD4L1//FOXD4//FOXD2//FOXD4L3
392366RPS14//RPS15//SRSF10//SMN2//SNRPD1//SNRPD2//NCBP2//RAD51//CENPA//OIP5//CENPI//H2BFM//H2
IPI2//ATG12//H2AFX//SOD1//SOD2//GTF2H3//RAD51AP1//RAD51//CLDN3//ALDOC//UBQLN1//IL1B//NPPC//SDC2
21790.0081057533.534351031HMGB2//CYCS//SOD1//FOXL2//TPD52L1//BAZ1B//RPL27A//RPS7//RPS14//RPS15
966025RPS14//RPS15//SRSF10//SMN2//SNRPD1//SNRPD2//NCBP2//RAD51//CENPA//OIP5//CENPI//H2BFM//H2
44//TBK1//IFI35//IFNG//STMN1//PLSCR1//EIF2AK2//ISG15//TAC1//CLDN4//CRH//EPYC//IL1B//SOD1//STC1//STC2
81410.0083586063.511250724RPS14//RPS15//WIPI2//ATG12//SRSF10//SMN2//SNRPD1//SNRPD2//NCBP2//RAD
53090.0084901533.501263916IL1B//PELI2//GH1//TGFBR1//SBDS//EIF4A3//SMURF1//MDM2//CCNB1//IFNG//IGF
ENPI//NPM1//CASC5//CENPH
2C//UBE2S//UBE2T//ANAPC11//UBE2W//UBE2D3
IGB2//CYCS//SOD1//FOXL2//TPD52L1//BAZ1B//RPL27A//RPS7//RPS14//RPS15//RPS24//RPL23//CTNNB1//MST4
05980.009170653.4551902IL1B//PELI2//GH1//TGFBR1//SMURF1//CCNB1//IFNG//IGF1//ADNP//VEGFA//UBE2C//F
14//RPS15//WIPI2//ATG12//SRSF10//SMN2//SNRPD1//SNRPD2//NCBP2//RAD51//CASC5//CENPA//OIP5//CENPI/
620.0103531873.396355941NDC80//SGOL1//CASC5//CCNB1
23420.0110096443.364172802BDKRB1//CCNB1//NLRP12//SOCS1//UBE2C//MAD2L1//ANAPC11//PSMA3//PSMA
2930.0110096443.363582589CDKN3//CKS2//SERTAD1//BCCIP//CCNE2//EGF//TGFBR1//IL1B//SOD1//SPRED2//
57457RPS15//NDC80//CENPA//DLGAP5//KIF18A//CCNB1//NUSAP1//KIF1B//RHOT1//BIRC5//CENPF

CDKN3//CKS2//SERTAD1//BCCIP//CCNE2//EGF//TGFBR1//IL1B//SOD1//SPRED2//FBXW11//UBR5//SMURF1//UBE
2431384WIPI2//ATG12//H2AFX//GTF2H3//RAD51AP1//RAD51//STRA13//RDM1//EYA2//UBE2T//NPM1//PCNA//PO
PI2//ATG12//NPM1//HAUS2//CENPA//CENPF//KIF11
PF//UBE2C//MAD2L1//ANAPC11//BUB1//DLGAP5//STAG2
A//CCNB1//BIRC5//CENPF
00204DLGAP5//KIF18A//CCNB1//CENPF//BIRC5
3021479CDKN3//CKS2//SERTAD1//BCCIP//CCNE2//EGF//TGFBR1//IL1B//SOD1//SPRED2//AKT1S1//IFI27//NLRP
CH6//UBE2C//UBE2T//UBE2D2//UBE2D3//UBE2G2
3045413.10142054CDKN3//CKS2//SERTAD1//BCCIP//CCNE2//EGF//TGFBR1//IL1B//SOD1//SPRED2//CCDC88A//
7//MARCH6//UBE2C//UBE2D2//UBE2D3//UBE2G2//FBXW11//UBR5//SMURF1
S14//RPS15//SRSF10//SMN2//SNRPD1//SNRPD2//NCBP2//RAD51//CENPA//OIP5//CENPI//H2BFM//H2AFX//H2AF
72490581B2M//CFDP1//IFI6//HSPA1B//BIRC5//IGF1//IL1B//NPM1//TRIAP1//BCL2A1//BNIP1//SOD1//YWHAZ//FEM
/SOD1//TGFBR1//CCNB2
10.0008889290.0204733173.051133006FOXD4L1//FOXD4//FOXD2//FOXD4L3
SEH1//RAD51//TOP1//POLE2//CCNE1//CCNE2//PCNA//RFC5//NAE1//STAG2//TOP2A
B2//CYCS//SOD1//FOXL2//TPD52L1//TOP2A
52053.023055426CENPF//UBE2C//MAD2L1//ANAPC11//BUB1//DLGAP5//DYNC1LI1
90653.012495559NPM1//CTNNB1//SKA2//STMN1//SBDS//CCNB1//DYNC1LI1//CYLD
XD4L1//FOXD4//FOXD2//FOXD4L3//TWSG1//TOB1//SMURF1//SMAD5//SMURF2//UBE2D3
790653.010193844SRSF10//SMN2//SNRPD1//SNRPD2//HNRNPM//NCBP2//SYNCRIP//LSM3//HNRNPA1//HNRNP
845120.0217185713.006779001IL1B//PELI2//BAZ1B//MTDH//GH1//CTNNB1//DAB2IP//GABPB1//HMGB2//IGF1//T
84208281CDKN3//CKS2//SERTAD1//BCCIP//CCNE2//EGF//TGFBR1//IL1B//SOD1//SPRED2//AKT1S1//IFI27//NLR
PO11//RPL23//TOB1//NLRP12//TXN//UBR5//EGF//DAB2IP//IL1B//CYLD//FBXW11//TGFBR1//HNRNPA1
770.024014862.955351574MAD2L1//DYNC1LI1//CENPF//UBE2C//ANAPC11//BUB1
331522.947071051EGF//TGFBR1//IL1B//SOD1//IFI27//NLRP12//CAP2//PRKACB//BDKRB1//DAB2IP//CYCS//GH1/
KPNA2//WIPI2//ATG12//CENPF//EGF//ORC6//IGF1//PCNA//POLE2//MCM10//RFC5//RRM2//PAPD5//TOP1//TOP2A
OL1//CTNNB1//DYNC1LI1//NEK2//HAUS2
140.0279331012.879553086CENPF//UBE2C//MAD2L1//ANAPC11//BUB1//DYNC1LI1
M//CFDP1//IFI6//HSPA1B//BIRC5//IGF1//IL1B//NPM1//TRIAP1//BCL2A1//BNIP1//SOD1//YWHAZ//FEM1B//GCH1//T
26892.854760708PTPN1//SOCS1//IFI6//HLA-B//IFI27//IFI35//OAS1//OAS2//ISG15
380.0289926892.854760708IFI6//HLA-B//IFI27//IFI35//OAS1//OAS2//PTPN1//SOCS1//ISG15
456579IFNG//KPNA2//IPO11//RPL23//TOB1//NLRP12//TXN//UBR5//EGF//DAB2IP//IL1B//CYLD//FBXW11//TGFBR1
59792.809957446IFI6//HLA-B//IFI27//IFI35//OAS1//OAS2//PTPN1//SOCS1//ISG15
5258931EIF2AK2//NXF1//SYNCRIP//CENPA//MAGI3//GRB2//ANPEP//TBK1//HLA-B//HNRNPA1//HNRNPK//HSPA8
42751412.773466581B2M//CFDP1//IFI6//HSPA1B//BIRC5//IGF1//IL1B//NPM1//TRIAP1//BCL2A1//BNIP1//SOD1//YW
650.0346729672.766048754HMGB2//CYCS//SOD1//FOXL2//TPD52L1//TOP2A//CTNNB1//MST4//STK24
SEH1//RAD51//TOP1//POLE2//CCNE1//CCNE2//PCNA//RFC5//ESCO2//CCDC88A//NAE1//STAG2//IGF1//CENPF//E
2//SERTAD1//BCCIP//CCNE2//EGF//TGFBR1//IL1B//SOD1//SPRED2//FBXW11//UBR5//SMURF1//UBE2D3//MDM2
process17197570145862.2082286940.0018899370.037410532.723552751SRSF10//SMN2//SNRPD1//SNRPD2//H
410532.723552751SRSF10//SMN2//SNRPD1//SNRPD2//HNRNPM//NCBP2//SRSF9//WBP4//JMJD6//SYNCRIP//LS
2743512.711297563CDKN3//CKS2//SERTAD1//BCCIP//CCNE2//EGF//TGFBR1//IL1B//SOD1//SPRED2//CCDC88A/
/IPO11//RPL23//SMURF1//STRADA//SDCBP//RTP4//TOMM5//YWHAZ//TOB1//NLRP12//TXN//UBR5//EGF//DAB2IF
/IPO11//RPL23//TOMM5//YWHAZ//TOB1//NLRP12//TXN//UBR5//EGF//DAB2IP//IL1B//CYLD//FBXW11//HSP90AA1/
388114282.698296915B2M//IL1B//PELI2//CCL26//HMGB2//VEGFA//IFNG//MYC//BDKRB1//EGF//BAZ1B//FEM1B//G
54563HMGB2//CYCS//SOD1//FOXL2//TPD52L1
10.0020523220.0389409432.687754563HMGB2//CYCS//SOD1//FOXL2//TPD52L1
54563DLGAP5//EGF//IGF1//IL1B//NUSAP1
09432.687754563EGF//IGF1//IL1B//NUSAP1//DLGAP5
682058072KPNA2//CENPF//EGF//ORC6//IGF1//PCNA//POLE2//MCM10//RFC5//RRM2//PAPD5//TOP1//TOP2A//NA
04892.664002003FOXD4L1//FOXD4//FOXD2//FOXD4L3//TWSG1//TOB1//SMURF1
02003NPM1//KIF11//SGOL1//CTNNB1//DYNC1LI1//HAUS2//NEK2
5178NPM1//CTNNB1//SKA2//STMN1//SBDS//CCNB1//DYNC1LI1//CYLD
0926FOXD4L1//FOXD4//FOXD2//FOXD4L3
0926FOXD4L1//FOXD4//FOXD2//FOXD4L3

36382.65120926STC1//VEGFA//STC2//CCNB1
0926STC1//VEGFA//STC2//CCNB1
2//IPO11//RPL23//SMURF1//STRADA//SDCBP//RTP4//TOMM5//YWHAZ//NPM1//PCNA//SYTL2//STX17//RIMS2//CT
3B//NPM1//HNRNPA1//NXF1//SRSF10//NCBP2//GLE1//UPF3B//U2AF1//SRSF9//KPNA2//IPO11//RPL23//SMURF1//
26077PRTFDC1//MAT2B//MAT2A//MCCC2//NUDT7//NUDT3
26077CENPF//UBE2C//MAD2L1//ANAPC11//BUB1//DYNC1LI1
26077PRTFDC1//MAT2B//MAT2A//MCCC2//NUDT7//NUDT3
65022.604626077CENPF//UBE2C//MAD2L1//ANAPC11//BUB1//DYNC1LI1
/OIP5//CENPI//NPM1//CASC5//CENPH//CHD1//BPTF
68095IFNG//KPNA2//IPO11//RPL23//TOB1//NLRP12//TXN//UBR5//EGF//DAB2IP//IL1B//CYLD//FBXW11//TGFBR1
7434664NDUFA8//NDUFB2//NDUFB4//NDUFB9//NDUFS6//UQCRB//COX6A1//COX6C//ATP5G1//CYCS//COX7A2L
/SOD1//FOXD4L1//FOXD4//FOXD2//FOXD4L3
1//UBE2C//BIRC5//UBE2S
6702RPS14//RPS15//RPS7//RPS24
04899642.526313541IL1B//PELI2//BAZ1B//MTDH//GH1//CTNNB1//DAB2IP//GABPB1//HMGB2//IGF1//TGFBR1//AR
NNB1//DYNC1LI1//NEK2
31//RRAGB//RRAGC//RHEBL1//CCDC88A
5007622.506266572EGF//TGFBR1//IL1B//SOD1//IFI27//NLRP12//CAP2//PRKACB//BDKRB1//DAB2IP//CYCS//GH1/
6495789CTNNB1//EGF//SYT9//IFNG//IL1B//PRKACB//SLC2A1//DAB2IP//NLRP12//TGFBR1//GH1//IGF1//INHBA//N
A2B//LAMP2//PPBP//SOD1//VEGFA//CALU//CD9
7246327IFNG//KPNA2//IPO11//RPL23//TOMM5//YWHAZ//TOB1//NLRP12//TXN//UBR5//MDM2//EGF//DAB2IP//IL1B
I2//SNRPD1//SNRPD2//BCAS2//HNRNPM//NCBP2//SYNCRIP//LSM3//HNRNPA1//HNRNPH3//HNRNPK//UPF3B//U
CBP2//GLE1//UPF3B//U2AF1//SRSF9//HNRNPA1//EIF5A2//ENY2//EIF4A3
29190292.352572598CTNNB1//BPTF//ZFPM2//SUZ12//FOSB//HSBP1//MDM2//NFX1//RPS14//TXN//MTDH//TCEAL
62660.0729565272.350406167CTNNB1//BPTF//ZFPM2//SUZ12//FOSB//HSBP1//MDM2//NFX1//RPS14//TXN//MTD
52052NME1//CMPK2//RRM2//NUDT7
RPS7//RPS14//RPS15//RPS24//RPL23//PUM2//NGDN//TACO1//EEF1E1//NCBP2//EIF5A2//SBDS//EIF4A3//CYLD//F
/IPO11//RPL23//SMURF1//STRADA//SDCBP//RTP4//TOMM5//YWHAZ//NPM1//PCNA//SYTL2//STX17//RIMS2//TO
CDKN3//CKS2//SERTAD1//BCCIP//CCNE2//EGF//TGFBR1//IL1B//SOD1//SPRED2//FBXW11//UBR5//SMURF1//UB
75385NXF1//SRSF10//NCBP2//GLE1//UPF3B//U2AF1//SRSF9//HNRNPA1
1646425IFNG//KPNA2//IPO11//RPL23//SMURF1//STRADA//SDCBP//RTP4//TOMM5//YWHAZ//NPM1//PCNA//SYTL
X1//RPS14//RPS15//SRSF10//SMN2//SNRPD1//SNRPD2//NCBP2//SBDS//RPS7//RPS24//UTP23//EIF4A3//SRSF9/
0.0838322532.274164801CENPF//UBE2C//MAD2L1//ANAPC11//BUB1//STAG2//MDM2//DYNC1LI1
39470.0838322532.272951282CENPF//TOP2A//CEP152//BIRC5//HSP90AA1//NEK2//HAUS2//TPD52L1//PPM1D//
10.005674430.0888023992.246077784CYLD//TBK1//UBE2D3//ATG12//ISG15
HNRNPA1//NXF1//SRSF10//NCBP2//GLE1//UPF3B//U2AF1//SRSF9//KPNA2//IPO11//RPL23//SMURF1//STRADA//S
TOP2A
5352.236631206NDC80//CENPA//NUSAP1
520.0901675422.232091151NLRP12//TXN//UBR5//EGF//DAB2IP//IL1B//CYLD//FBXW11//TGFBR1
6278257NLRP12//TXN//UBR5//EGF//DAB2IP//IL1B//CYLD//FBXW11//NCBP2//TGFBR1
80.0942839842.209069173FOXD4L1//FOXD4//FOXD2//FOXD4L3//CTNNB1//MYC
4//RPS15//SRSF10//SMN2//SNRPD1//SNRPD2//NCBP2//SRSF9//NPM1
F10//NCBP2//SMN2//SNRPD1//SNRPD2
83120.100039092.177942333PSMA8//PSMA3//PSMA6//PSMA7//PSMB5//PSMC5//PSMD9//PSMD11//PSMD12//PS
/MYC//FOXD4L1//FOXD4//FOXD2//FOXD4L3//DCN//JMJD6//TGFBR1//CA2
//CENPA//DLGAP5//KIF18A//CCNB1//NUSAP1//KIF1B//RHOT1//BIRC5//CENPF
7758872.128337998CTNNB1//TBK1//IL1B//MAP3K3//ZDHHC13//PPM1A//PELI2//BST2//MTDH//DAB2IP//C9ORF89
NDC80//CENPA//CCDC88A//SDCBP//CTNNB1//STMN1//KIF18A//UBE2C//ZWINT//CKS2//KIF11//AURKA//SPC25//
B1//MYC//FOXD4L1//FOXD4//FOXD2//FOXD4L3
99544PRTFDC1//MAT2B//MAT2A
DC1//MAT2B//MAT2A
99544PRTFDC1//MAT2B//MAT2A
URKC//PRC1//KIF23//KLHL21
31987NXF1//GLE1//SRSF10//NCBP2//UPF3B//U2AF1//SRSF9

DC1//NME1//MAT2B//MAT2A//MCCC2//NUDT7//NUDT3
380.1155379692.094453431BRIX1//RPS14//RPS15//SRSF10//SMN2//SNRPD1//SNRPD2//NCBP2//SBDS//RPS7//
4432.091700652EGF//IGF1//IL1B//NUSAP1//DLGAP5//BIRC5//MDM2//CCNB1
05920.1214101472.069530233CTNNB1//BPTF//ZFPM2//SUZ12//FOSB//HSBP1//MDM2//NFX1//RPS14//TXN//MTD
N//JMJD6//TGFBR1//CA2//CTNNB1//IFNG//MYC//FOXD4L1//FOXD4//FOXD2//FOXD4L3
1//COX6C//NDUFA8//NDUFB2//NDUFB4//NDUFB9//NDUFS6//ATP5G1//CYCS//UQCRB//COX7A2L
CENPA//OIP5//CENPI//H2BFM//H2AFX//H2AFZ//HMGB2//NAP1L4//NPM1//H2AFJ//CASC5//CENPH//HIST2H2AC/
3830544TNNI3//TPM1//SOD2//GCH1//BDKRB1//NCF2//UTS2//HBB//NPPC//SOD1
87191RPS14//RPS15//SRSF10//SMN2//SNRPD1//SNRPD2//NCBP2//SRSF9//NPM1
/IFNG//IL1B//PRKACB//SLC2A1//INHBA//NMU//SLC30A8//GAL//FAM3B//PSMD9//SIRT4//CRH
NPA1//NXF1//SRSF10//NCBP2//GLE1//UPF3B//U2AF1//SRSF9//EIF5A2//ENY2//EIF4A3
1//SRSF10//NCBP2//GLE1//UPF3B//U2AF1//SRSF9//EIF5A2//ENY2//EIF4A3
588469HNRNPA1//NXF1//SRSF10//NCBP2//GLE1//UPF3B//U2AF1//SRSF9//EIF5A2//ENY2//EIF4A3
020.0093637480.1284271412.028550264BDKRB1//CCNB1//EIF4A3//NLRP12//SOCS1//UBE2C//MAD2L1//ANAPC
N//JMJD6//TGFBR1//CA2//CTNNB1//IFNG//FEM1B//HOXB13//MYC//FOXD4L1//FOXD4//FOXD2//FOXD4L3
STMN1//KIF18A
/CASC5//CCNB1
910.1297046862.016193771FOXD4L1//FOXD4//FOXD2//FOXD4L3
93771CCNB1//JMJD6//SRSF10//CDK11A
036330.1301637482.013065648IL1B//PELI2//BAZ1B//GH1//CTNNB1//DAB2IP//GABPB1//HMGB2//IGF1//TGFBR1/
5//RAD51//TOP2A
6A1//COX6C//NDUFA8//NDUFB2//NDUFB4//NDUFB9//NDUFS6//ATP5G1//CYCS//UQCRB//COX7A2L//TXN
3871.998705192UBE2C//BIRC5//KNTC1
590.1316463871.998705192DAB2IP//C9ORF89//NLRP12
05192NDC80//CENPA//NUSAP1
/NUSAP1
RP12//IL1B//CTNNB1//TBK1//MAP3K3//ZDHHC13//PPM1A//PELI2//BST2//MTDH//DAB2IP//C9ORF89//NKIRAS1//
991376318PCNA//POLE2//RFC5//RRAGC//CDK11A//MORF4L2//SOD1//TNNI3//IFNG//YWHAZ//TPM1//SOD2//EG
SFXN1//HMGB2//JMJD6//BPGM//RPS24
F1//SRSF10//NCBP2//GLE1//UPF3B//U2AF1//SRSF9//EIF5A2//ENY2//EIF4A3
MN2//SNRPD1//SNRPD2
process649570145863.1334049410.0116485730.1494037061.933727289TGFBR1//FOXD4L1//FOXD4//FOXD2//F
5861.1379091560.011668340.1494037061.932990936KPNA2//CENPF//EGF//ORC6//IGF1//PCNA//POLE2//MCM1
4120.0120423310.1536551071.919289443HNRNPA1//NXF1//SRSF10//NCBP2//GLE1//UPF3B//U2AF1//SRSF9//V
//PCNA//POLE2//RFC5//RAD51AP1//H2AFX//RAD51//TRIP13//HMGB2//RDM1//RAD51C//MND1
445881.904866029CCNB1//TRIP13//CASC5//TDRD6//GAPDHS//HMGB2//CD9//TGFBR1//RPL27A//RPS7//RPS14
79397INHBA//CRH//IL1B
09264PRTFDC1//SOD1//CDO1//SDS//RRAGB//RRAGC//ALDOC//IL1B//CCNE1
//CTNNB1//STAG2//RAD51C
FNG//IL1B//PRKACB//SLC2A1//INHBA//NMU//SLC30A8//GAL//FAM3B//PSMD9//SIRT4//CRH
19590.1724124231.858802454BDKRB1//CCNB1//EIF4A3//NLRP12//MAD2L1//SOCS1//UBE2C//ANAPC11//PSMA
//SNRPD1//SNRPD2//HNRNPM//NCBP2//SYNCRIP//LSM3//HNRNPA1//HNRNPH3//HNRNPK//UPF3B//U2AF1//SF
50749GCH1//HBB//HSP90AA1//IFNG//IL1B//WASL
4591.852150749NLRP12//EGF//UBR5//IL1B//NCBP2//TGFBR1
4591.852150749NCF2//SOD1//SOD2//NOX4//HBB//HP
491.842195348NLRP12//TXN//UBR5//EGF//DAB2IP//IL1B//CYLD//FBXW11//TGFBR1
06149141.829843476SOD1//B2M//IL1B//PELI2//CCL26//HMGB2//VEGFA//IFNG//MYC//TBK1//BDKRB1//TPM1//C
1AP1//H2AFX//RAD51//PCNA//POLE2//RFC5//HMGB2//SOD1//BAZ1B//TRIP13//STRA13//RDM1//EYA2//UBE2T//
944045SYT9//IFNG//IL1B//PRKACB//SLC2A1//NLRP12//INHBA//NMU//RAB3A//SLC30A8//PSMD9//TXN//UBR5//E
1//STC2
DHS//PRKACB//TPI1
26397HSP90AA1//WASL//GCH1//IFNG//IL1B
PM1
/NOX4//SOD1

M1//ESCO2//UBE2C//GGCX//UBE2S//UBE2T//MAN2A1//UBE2W//EIF5A2//UBE2D2//UBE2D3//UBE2G2//B4GALT4
G//KPNA2//IPO11//RPL23//SMURF1//STRADA//SDCBP//RTP4//TOMM5//YWHAZ//NPM1//PCNA//SYTL2//STX17//R
3248713NLRP12//TXN//UBR5//EGF//DAB2IP//IL1B//CYLD//FBXW11//NCBP2//TGFBR1
117570145862.1871345030.0162836320.1917384611.788248713TGFBR1//SMURF2//SMURF1//FOXD4L1//FOXD4
DC1//ERH//NME1//MAT2B//MAT2A//MCCC2//NUDT7//NUDT3
//KPNA2//IPO11//RPL23//SMURF1//STRADA//SDCBP//RTP4//TOMM5//YWHAZ//NPM1//PCNA//SYTL2//STX17//RI
DKN3//CKS2//SERTAD1//BCCIP//CCNE2//EGF//TGFBR1//IL1B//SOD1//SPRED2//CCDC88A//SOCS1//BDKRB1//C
DKN3//CKS2//SERTAD1//BCCIP//CCNE2//EGF//TGFBR1//IL1B//SOD1//SPRED2//CCDC88A//SOCS1//BDKRB1//C
30154FOXD4L1//FOXD4//FOXD2//FOXD4L3
30154TXN//IL1B//SMURF1//STRADA
50.0174148540.1974402371.759080154ARPP19//IGF1//GAPDHS//IFNG
04//FOXD2//FOXD4L3
30154CLDN4//NCF2//SOCS1//CCNE1
50.0174148540.1974402371.759080154HBB//HSP90AA1//IFNG//IL1B
540.1974402371.759080154ARPP19//IGF1//GAPDHS//IFNG
04L1//FOXD4//FOXD2//FOXD4L3
IPF//CTNNB1//GCLM//IFNG//IL1B//MAT2A//MYC//NCF2//NME1//NPPC//GAL//B2M//ABCD3//SOD1//TOP1//YWHAZ
966771.726736822EGF//TGFBR1//GH1//IGF1//PPM1A//FOXD4L1//FOXD4//FOXD2//FOXD4L3//TWSG1//VEGFA//
CENPA//NMU//FOXD4L1//FOXD4//FOXD2//FOXD4L3//YWHAZ//VEGFA//EGF//IGF1//ITGA2B//LAMP2//PPBP//SOD
9011.720887957GCH1//IFNG//IL1B
A//CCNB1//CENPF
37957BUB1//CCNB1//CTNNB1
3591471.705039698EGF//DAB2IP//TGFBR1//GH1//IGF1//PPM1A//FOXD4L1//FOXD4//FOXD2//FOXD4L3//TWSG1/
67821.701845158NDUFA8//NDUFB2//NDUFB4//NDUFB9//NDUFS6//UQCRB
320.2191467821.701845158NDUFA8//NDUFB2//NDUFB4//NDUFB9//NDUFS6//UQCRB
2039617BAZ1B//RPL27A//RPS7//RPS14//RPS15//RPS24//RPL23//STMN1//KIF18A//SBDS//CCNB1//EIF5A2
40338STMN1//KIF18A//SKA2//SBDS//CCNB1
362071EGF//DAB2IP//TGFBR1//GH1//IGF1//PPM1A//FOXD4L1//FOXD4//FOXD2//FOXD4L3//TWSG1//VEGFA//RR
90381.666702578NLRP12//UBR5//EGF//IL1B//NCBP2//TGFBR1
02578TOB1//TWSG1//CTNNB1//IGF1//JUND//NPPC
02578HSP90AA1//WASL//GCH1//HP//IFNG//IL1B
//PTPN1//SOCS1//HLA-B//OAS1//OAS2//B2M
Z1B//RPL27A//RPS7//RPS14//RPS15//RPS24//RPL23//STMN1//KIF18A//SBDS//CCNB1//EIF5A2
RPS15//IFNG//NDC80//CENPA//NMU//YWHAZ//EGF//IGF1//ITGA2B//LAMP2//PPBP//SOD1//VEGFA//CALU//CD9//S
NG//KPNA2//IPO11//RPL23//SMURF1//STRADA//SDCBP//RTP4//TOMM5//YWHAZ//TOB1//NLRP12//TXN//UBR5//E
3521381.649050991KPNA2//ESCO2//PCNA//CCDC88A//NAE1//MYC//STAG2//H2AFX//IGF1//NPPC//RAD51
/SOD2//NCF2//NOX4
//CRH//IL1B
//IFNG//OAS1//OAS2//B2M//PTPN1//SOCS1//GCH1
IMJD6//BPGM//RPS14//SFXN1
97871.63245963DAB2IP//CYLD//FBXW11//TXN//DPH3//NLRP12
RAD51//UBR2//SGOL1//STAG2//H2AFX//NEK2//MND1
37350.245393081.629787984TRIP13//TOP2A//STAG2//H2AFX//NEK2//RAD51//MND1//SYCP2//UBR2//SGOL1
31160.2465654441.626479013SOD1//TPM1//NPPC//NMU//IL1B//CRH//IFNG//AKIRIN2//GH1//CA2//PANX1//NLRP
21981.616944826SOD1//INHBA//RPS14//SFXN1//RPS24//CCNB2//HMGB2//JMJD6//BPGM
5240169RPL27A//RPS7//RPS14//RPS15//RPS24//RPL23//GCH1//MDM2//PARD6B//CCNB1//WASL//REPS2//KNTC
9116816UBE2C//UBE2S//IL1B//MYC//CA2//HMGB2//INHBA//BDKRB1//GCLM//IFI6//SLC30A8
1608771.608381286CENPF//UBE2C//MAD2L1//ANAPC11//BUB1//NPM1//STMN1//SBDS//CCNB1//DYNC1LI1
590.257914331.597808126NLRP12//IL1B//CYLD//FBXW11
8126NLRP12//IL1B//CYLD//FBXW11
8126FOXD4L1//FOXD4//FOXD2//FOXD4L3
680.257914331.595940367NDUFA8//NDUFB2//NDUFB4//NDUFB9//NDUFS6
0367HBB//HSP90AA1//IFNG//IL1B//GCH1
P13//TOP2A//STAG2//H2AFX//NEK2//RAD51//MND1//SYCP2//UBR2//SGOL1

5490.0265335170.2677385351.576205189SMAD5//SMURF1//SMURF2//TGFBR1//UBE2D3//GDF15//TOB1//FOXD
35351.571434934CTNNB1//EGF//SMURF1
34934EGF//UBR5//DAB2IP
650.2677385351.571434934EGF//UBR5//DAB2IP
35351.571434934GCLM//IFI6//SOD1
0159639RPL27A//RPS7//RPS14//RPS15//RPS24//RPL23//STMN1//KIF18A//SBDS//CCNB1//EIF5A2
0159639SYT9//IFNG//IL1B//PRKACB//SLC2A1//INHBA//NMU//SLC30A8//PSMD9//SIRT4//CRH
GGCX//GRB2//HBB//IGF1//ITGA2B//ITGAM//KIF11//RHOB//LAMP2//PPBP//PRKACB//PTPN1//RAB5A//RAD51C//S
70.0277176660.2743299841.557243338NLRP12//EGF//UBR5//IL1B//TGFBR1
NASEH1//MRPL12//TOMM5//YWHAZ//NDUFAF4//LETM1//HSP90AA1//MAN2A1//RAB3A
640.2766764721.548874508DCP2//NCBP2//UPF3B//EIF4A3
04L1//FOXD4//FOXD2//FOXD4L3
54721.548874508CTNNB1//IGF1//JUND//NPPC
L27A//RPS7//RPS14//RPS15//RPS24//RPL23//STMN1//KIF18A//SBDS//CCNB1//EIF5A2
5//SBDS//RPS7//RPS24//UTP23//EIF4A3//NPM1//SDAD1//BRIX1
1//CENPI//INHBA//SOD1//FOXL2
690.292726221.519764915SOD1//SOD2//HBB//HP//TPM1
59861.504734458PCNA//POLE2//RFC5
59861.504734458NME1//RRM2//TXN
P2//CTNNB1//HSD17B3
10.0312799130.2993559861.504734458IL1B//PTPN1//SOCS1
/ALDOC//GAPDHS//PRKACB//TPI1//ARPP19
340.2993559861.502069663EIF5A2//MAT2B//MAT2A//GCLM
59861.502069663SAT1//SMS//ASMT//GCH1
488130364SOD1//SOD2//TAS2R14//RTP4//CLDN3//ALDOC//UBQLN1//IL1B//NPPC//SDC2//VEGFA//PRTFDC1//B2
45709HLA-B//IFNG//OAS1//OAS2//B2M//PTPN1//SOCS1
18811.483447985NLRP12//EGF//UBR5//IL1B//TGFBR1
2.7814645310.0328512590.3085218811.483447985MDM2//UBR5//HERC6//SMURF1//SMURF2
//CTNNB1//WNT9A//FOXD4L1//FOXD4//FOXD2//FOXD4L3//LUM
38280.3123829091.476854317MTDH//DCN//GCH1//IL1B//NCF2//AKIRIN2//BDKRB1//SOCS1//CMPK2//HMGB2//B
2//NDUFB4//NDUFB9//NDUFS6//UQCRB
510.3131089341.47363033MTDH//MDM2//GAPDHS//IGF1//IFNG//IL1B
81521.471308857SPRED2//AKT1S1//SSTR2//TFPI//CAST//TNNI3//DAB2IP//IFI6//BIRC5//IL1B//HP//UBE2C//MAD
L1B//PRKACB//SLC2A1//SLC30A8//PSMD9//SIRT4//GAL//FAM3B
MKK2//MAP3K3//EGF//TGFBR1//IL1B//SOD1//SPRED2//PSMA8//MDM2//TRIAP1//PSMA3//PSMA6//PSMA7//PSM
34261.457249457DAB2IP//CYLD//FBXW11//TXN
261512551.449409493KPNA2//CENPF//EGF//ORC6//IGF1//PCNA//POLE2//MCM10//RFC5//RRM2//PAPD5//TOP1//
39294SOD1//FOXD4L1//FOXD4//FOXD2//FOXD4L3
780.3261512551.448239294CYLD//TBK1//UBE2D3//ATG12//ISG15
RF1//TOB1//TWSG1//CTNNB1//IGF1//JUND//NPPC//RSAD2
cycleBiolog process319570145864.0404432130.0360949360.3262575711.442553725PCNA//RRM2//CCNE1

75711.442553725IFNG//PTPN1//SOCS1
75711.442553725IFNG//PTPN1//SOCS1
441060383KPNA2//CENPF//EGF//ORC6//IGF1//PCNA//POLE2//MCM10//RFC5//RRM2//PAPD5//TOP1//TOP2A//NA
7A//RPS7//RPS14//RPS15//RPS24//RPL23//TOP2A//NCBP2//GTF2H3//PSMA8//ZNF483//B2M//PSMA3//PSMA6//P
F1//ITGA2B//RHOB//LAMP2//PLSCR1//PPBP//SOD1//VEGFA//YWHAZ//CALU//CD9//ZFPM2//GGCX//GRB2//HBB//
2H3//HMGB2//CYCS//SOD1//FOXL2//TPD52L1
3R1//INHBA//CCNB1//NCF2//PCNA
24857IL1B//INHBA//SIRT4//PSMD9//DPH3//NLRP12//EGF
PNA2//CENPF//EGF//ORC6//IGF1//PCNA//POLE2//MCM10//RFC5//RRM2//PAPD5//TOP1//TOP2A//NAE1//CDKN3/
2//YWHAZ//CTNNB1//EGF//CCL26//NUDT3//GRB2//IL1B//INHBA//CCL7//SSTR2//STC1//BST2//TAC1//TNFAIP6//T
056941.397585088EIF2AK2//DAB2IP//IFNG//GAL//ABI1//TOB1//CDKN3//BTG3//CTNNB1//HSPA1B//IL1B//INHBA//N
2//HBB//IGF1//ITGA2B//ITGAM//KIF11//RHOB//LAMP2//PPBP//PRKACB//PTPN1//RAB5A//RAD51C//SLC16A1//SO

2//HBB//IGF1//ITGA2B//ITGAM//KIF11//RHOB//LAMP2//PPBP//PRKACB//PTPN1//RAB5A//RAD51C//SLC16A1//SO
79135SSTR2//CTNNB1//GH1//IL1B//AURKA//SOCS1//CCNE1
73611.384397475AKT1S1//RRAGB//RRAGC
650.3606673611.384397475INHBA//LAG3//NLRP12
441.380950903HMGB2//CYCS//SOD1//FOXL2//TPD52L1
//ALDOC//GAPDHS//PRKACB//TPI1//ARPP19//GCH1//SPTLC1
75921546PRTFDC1//SOD1//B2M//HSPA1B//HSPA6//HSPA8//HSP90AA1//BAIAP2//GRB2//PPM1A//STRADA//MAT
60.0423589570.3650994371.373054738IFNG//GH1//SOCS1//IGF1
570.3650994371.373054738DAB2IP//CYLD//FBXW11//TXN
DH//CMPK2//HMGB2//DCN//GCH1//IL1B//NCF2//AKIRIN2//BDKRB1//SOCS1
E2C//PSMA8//ORC6//MAD2L1//MDM2//ANAPC11//MCM10//PSMA3//PSMA6//PSMA7//PSMB5//PSMC5//PSMD9//P
4990.0430464380.3691710881.366062784BAZ1B//CTNNB1//DAB2IP//GABPB1//HMGB2//IGF1//IL1B//ADNP//NPP
C//GAPDHS//BPGM//TPI1//KIF1B//DHDH//IGF1//NUDT3
948611CDO1//SOD1//TPST1//GCLM//SMS//MAT2B//MAT2A//CTNNB1//DCN//IMPAD1
490331.350638859EGF//ZFPM2//GGCX//GRB2//HBB//IGF1//ITGA2B//ITGAM//KIF11//RHOB//LAMP2//PPBP//PRK
A//PPP3CA//PTPN1//PPM1D//CDC14A//EYA2//DUSP14//PPP6R3//IMPA1//IFNG
1//EIF5A2//MAT2B//MAT2A//SAT1//SMS//GCLM//ASMT//GCH1
BI1//IFNG//SOCS1//IGF1//EGF//ADNP//VEGFA
1//CD9//SOD1//FOXD4L1//FOXD4//FOXD2//FOXD4L3
14494CTHRC1//HMGB2//NME1//TXN//IL1B//RHEBL1//NPM1//PSMA6//MTDH//DPH3
//FOXD2//FOXD4L3//CTNNB1//HNRNPAB//DAB2IP//MYC
249NAE1//POLE2//CCNE1//CCNE2
249FOXD4L1//FOXD4//FOXD2//FOXD4L3
60.386198351.333455249STMN1//SBDS//CCNB1//SKA2
60.386198351.333455249HBB//HSP90AA1//IFNG//IL1B
RIM23
YCP2
660.3867829651.329847275ARPP19//IGF1//GAPDHS
44402PRTFDC1//SOD1//CDO1//SDS//RRAGB//RRAGC

SMA6//PSMA7//PSMB5//PSMC5//PSMD9//PSMD11//PSMD12//PSME1//RRM2//SKP1//CCNB1//CCNE1//CCNE2//PS
0//PSMA3//PSMA6//PSMA7//PSMB5//PSMC5//PSMD9//PSMD11//PSMD12//PSME1//RFC5//CCNB1//CCNB2//CCN
3//PSMA6//PSMA7//PSMB5//PSMC5//PSMD9//PSMD11//PSMD12//PSME1//RFC5//CCNB1//CCNB2//CCNE2//H2A
PSMA3//PSMA6//PSMA7//PSMB5//PSMC5//PSMD9//PSMD11//PSMD12//PSME1//RRM2//SKP1//CCNB1//CCNE1//
A2//CASC5//SPC25//CENPH//BUB1//KIF18A//KNTC1//DLGAP5//CCNB1//TRIP13//TOP2A//UBE2C//CKS2//KIF11//
//PSMD9//PSMD11//PSMD12//PSME1//RFC5//CCNB1//CCNB2//CCNE2//H2AFX//CDKN3//CKS2//SERTAD1//BCCI
5//PSMC5//PSMD9//PSMD11//PSMD12//PSME1//RFC5//CCNB1//CCNB2//CCNE2//H2AFX//TRIAP1//BUB1//NEK2/
//CENPI//BIRC5//CASC5//SPC25//CENPH//BUB1//KIF18A//CCNB1//KNTC1//USP16//UBE2C//OIP5//CDCA2//KIF11
2//UBE2C//ZWINT//OIP5//CKS2//SGOL1//CDCA2//SENP5//UBE2S//SKA2//KIF11//MAD2L1//NEK2//DYNC1LI1//ANA
D12//PSME1//NEK2//CDK11A//ZWINT//MAD2L1//BUB1//KNTC1//CENPF//UBE2C//ANAPC11//BIRC5//NAE1//WNT9
UB1//KIF18A//CCNB1//KNTC1//USP16//UBE2C//OIP5//CDCA2//KIF11//DYNC1LI1//ANAPC11//TXNL4B//HAUS2//P
A//CCNB1//KNTC1//DLGAP5//CDK11A//UBE2C//ANAPC11//KIF11//EGF//IGF1//IL1B//USP16//OIP5//CDCA2//DYN
WINT//MAD2L1//BUB1//KNTC1//CENPF//UBE2C//ANAPC11//NAE1//WNT9A//BIRC5//TOP2A//CASC5//CCNB1//DY
B1//KIF18A//CCNB1//KNTC1//USP16//UBE2C//OIP5//CDCA2//KIF11//DYNC1LI1//ANAPC11//TXNL4B//HAUS2//PE
B5//PSMC5//PSMD9//PSMD11//PSMD12//PSME1//RFC5//CCNB1//CCNB2//CCNE2//H2AFX//TRIAP1//ZWINT//BUE
SMD11//PSMD12//PSME1//RFC5//CCNB1//CCNB2//CCNE2//H2AFX//TRIAP1//ZWINT//BUB1//KNTC1//CENPF//NA
//CENPF//UBE2C//MAD2L1//ANAPC11//BUB1//NAE1//WNT9A//TOP2A//CCNB1//DYNC1LI1//ZWINT//KNTC1
B5//PSMC5//PSMD9//PSMD11//PSMD12//PSME1//RFC5//CCNB1//CCNB2//CCNE2//H2AFX//TRIAP1//CDKN3//SES
PSMB5//PSMC5//PSMD9//PSMD11//PSMD12//PSME1//SKP1//AURKA//CCNB1//PTTG1
F//CTNNB1//BIRC5//SKA2//SPC25//CENPH//PTTG1
D11//PSMD12//PSME1//UBE2C//BIRC5//EGF//IGF1//IL1B//NUSAP1//DLGAP5
12//PSME1//PSMA8//SKP1//CCNB1//FEM1B//UBE2S
SMD9//PSMD11//PSMD12//PSME1//RRM2//SKP1//CCNB1//CCNE1//CCNE2//PSMA8//RFC5//CENPF//KPNA2//CEF
NB1//KNTC1
GB2//CYCS//SOD1//FOXL2//TPD52L1//RNASEH1//HSPA1B//PSMA8//PSMA3//PSMA6//PSMA7//PSMB5//USP16//U
KP1//CCNB1//CCNE1//CCNE2//PSMA8//RFC5//CENPF//KPNA2//CEP152//BIRC5//HSP90AA1//NEK2//HAUS2//TPI
PSMA6//PSMA7//PSMB5//PSMC5//PSMD9//PSMD11//PSMD12//PSME1//SKP1//CCNB1//CCNE2
DC88A//CENPF//STAG2//SGOL1//CENPI//BIRC5//CASC5//SPC25//CENPH//BUB1//KIF18A//CCNB1//KNTC1//PCN
MD12//PSME1//PSMA8//SKP1//CCNB1//UBE2S//FEM1B
1//PSMD12//PSME1//SKP1//CCNB1//UBE2S
D2L1//NEK2//NUSAP1//SKA2//CENPA//CCDC88A//CENPF//STAG2//SGOL1//CENPI//BIRC5//CASC5//SPC25//CEN
G2//PTTG1//NDC80//ZWINT//MAD2L1//NEK2//NUSAP1//SKA2//CENPA//CCDC88A//CENPF//STAG2//SGOL1//CE

ANAPC11//PSMA3//PSMA6//PSMA7//PSMB5//PSMC5//PSMD9//PSMD11//PSMD12//PSME1//PSMA8//SKP1//CCNE
//CTNNB1//SKA2//NUSAP1//SBDS//CCNB1//DYNC1LI1//HAUS2//NEK2//CYLD//CCDC88A
ARCH6//UBE2C//DCAF4//TRIM23//ANAPC11//PCNP//SKP1//UBE2D2//KLHL21//UBE2W//SUZ12//NAE1//UBQLN1//
F2//FEM1B//DCAF7//MARCH6//UBE2C//DCAF4//TRIM23//ANAPC11//PCNP//SKP1//UBE2D2//KLHL21//UBE2W//S

SMA6//PSMA7//PSMB5//PSMC5//PSMD9//PSMD11//PSMD12//PSME1//PSMA8//SKP1//CCNB1
SMC5//PSMD9//PSMD11//PSMD12//PSME1//SKP1//AURKA//CCNB1//PTTG1//FBXW11//MDM2//PCNP//SMURF1//
/D11//PSMD12//PSME1//SKP1//CCNB1//UBE2S
D9//PSMD11//PSMD12//PSME1//SKP1//AURKA//CCNB1//PTTG1//FBXW11//MDM2//PCNP//SMURF1//UBE2D3
/A7//PSMD9//SKP1//UBE2D2//UBE2D3//UBE2G2//USP13//NPLOC4//UBE2S//MAD2L1//ANAPC11//PSMB5//PSMC
A6//PSMA7//PSMD9//SKP1//UBE2D2//UBE2D3//UBE2G2//USP13//NPLOC4//UBE2S//MAD2L1//ANAPC11//PSMB
//PSMA3//PSMA6//PSMA7//PSMB5//PSMC5//PSMD9//PSMD11//PSMD12//PSME1//SKP1//CCNB1
/A3//PSMA6//PSMA7//PSMB5//PSMC5//PSMD9//PSMD11//PSMD12//PSME1//SKP1//AURKA//CCNB1//PTTG1//FB
MA8//CYLD//UBR2//PSMA3//PSMA6//PSMA7//PSMD9//SKP1//UBE2D2//UBE2D3//UBE2G2//USP13//NPLOC4//UE

//H2AFZ//HMGB2//NAP1L4//NPM1//H2AFJ//CASC5//CENPH//HIST2H2AC//H2AFV//EZH2//BPTF//STAG2//RAD51
JBE2C//CYLD//UBR2//PSMD9//SKP1//UBE2D2//UBE2D3//UBE2G2//USP13//NPLOC4//UBE2S//MAD2L1//ANAPC1
/SOD1//FOXL2//TPD52L1//RNASEH1//HSPA1B//PSMA8//PSMA3//PSMA6//PSMA7//PSMB5//USP16//UBE2C//CYL
SMD12//PSME1//PSMA8//SKP1//CCNB1//FEM1B//UBE2S//UBQLN1
CENPA//CCDC88A//CENPF//STAG2//SGOL1//CENPI//BIRC5//CASC5//SPC25//CENPH//BUB1//KIF18A//CCNB1//K
25313PSMA8//MDM2//TRIAP1//PSMA3//PSMA6//PSMA7//PSMB5//PSMC5//PSMD9//PSMD11//PSMD12//PSME1
/D9//PSMD11//PSMD12//PSME1
/D9//PSMD11//PSMD12//PSME1
/D9//PSMD11//PSMD12//PSME1
SMC5//PSMD9//PSMD11//PSMD12//PSME1
/D9//PSMD11//PSMD12//PSME1
TRIAP1//PSMA3//PSMA6//PSMA7//PSMB5//PSMC5//PSMD9//PSMD11//PSMD12//PSME1
/PSMB5//PSMC5//PSMD9//PSMD11//PSMD12//PSME1
D2L1//NEK2//NUSAP1//SKA2//CENPA//CCDC88A//CENPF//STAG2//SGOL1//CENPI//BIRC5//CASC5//SPC25//CEN
SMD12//PSME1//SKP1//CCNB1//UBE2S//SMURF1
D11//PSMD12//PSME1

//UBR2//PSMD9//SKP1//UBE2D2//UBE2D3//UBE2G2//USP13//NPLOC4//UBE2S//MAD2L1//ANAPC11//PSMC5//PS
PH//HIST2H2AC//H2AFV//BAZ1B//CENPF
C25//NPM1//SGOL1//CASC5//CTNNB1//SKA2//NUSAP1//SBDS//CCNB1//DYNC1LI1//RHOT1//HAUS2//NEK2//CYL
D11//PSMD12//PSME1//WNT9A

1//PSMA3//PSMA6//PSMA7//PSMB5//PSMC5//PSMD9//PSMD11//PSMD12//PSME1
9//PSMD11//PSMD12//PSME1
3//IDH3A//RAB31//GCH1//NKIRAS1//TRIM23//RHOB//RAB22A//RAB3A//RAB5A//RRAGC//RAB7L1//RAB33A//KIF1
PSMC5//PSMD9//PSMD11//PSMD12//PSME1
FV//TOP1//TOP2A//RAD51//NUSAP1//CCNB1
/TOB1//NLRP12//TXN//UBR5//EGF//DAB2IP//IL1B//CYLD//FBXW11//TGFBR1//NPM1
PSMC5//PSMD9//PSMD11//PSMD12//PSME1//WNT9A
/NPM1//TOB1//NLRP12//TXN//UBR5//EGF//DAB2IP//IL1B//CYLD//FBXW11//TGFBR1

I1
2AC//H2AFV//CENPF
/D12//PSME1
SMD11//PSMD12//PSME1
2BFM//H2AFX//H2AFZ//HMGB2//NAP1L4//NPM1//H2AFJ//CASC5//CENPH//HIST2H2AC//H2AFV//BAZ1B//SRSF9//
B
P1//TOP2A//CCNB1

1//HMGB2//BIRC5//IL1B//RHOB//TRIAP1//WWOX//MST4//FAM3B//CYCS//RHOT1//PSMA3//PSMA6//PSMA7//PSM
SMB5//PSMC5//PSMD9//PSMD11//PSMD12//PSME1//RFC5//CCNB1//CCNB2//CCNE2//H2AFX//CTNNB1//BPTF//Z
/BIRC5//IL1B//RHOB//TRIAP1//WWOX//MST4//FAM3B//CYCS//RHOT1//PSMA3//PSMA6//PSMA7//PSMB5//PSMC
PSMD12//PSME1
SME1//PRTFDC1

H1//UBQLN1//HMGB2//BIRC5//IL1B//RHOB//TRIAP1//WWOX//MST4//FAM3B//CYCS//RHOT1//PSMA3//PSMA6//PS
MURF1//UBE2D3//ISG15//MDM2//HERC6//SMURF2//CCDC88A//SOCS1//BDKRB1//CCNB1//PELI2//CCT7//DNAJC

NPH3//HNRNPK//U2AF1//SRSF9//GTF2H3//BCAS2//WBP4//SNRNP48//JMJD6//SREK1IP1//TXNL4B//HSPA1B//CC
EM1B//GCH1//TFPT//INHBA//WWOX//IFI27//NLRP12//CTNNB1//MST4//STK24//RHOB//NGFRAP1//CRH//PSMA8//
NAE1//FEM1B

C5//RRM2//PAPD5//TOP1//TOP2A//NAE1//NDC80//SGOL1//BIRC5//SKA2//NEK2//CASC5//SPC25//CENPH//PTTG
BCCIP//RAD51C//RFC5//TOP2A//UBE2D3//PTTG1//MORF4L2//HMGB2//SOD1//BAZ1B//TRIP13//PSMA8//MDM2//T

7//PSMB5//PSMC5//PSMD9//PSMD11//PSMD12//PSME1//EEF1E1
SMB5//PSMC5//PSMD9//PSMD11//PSMD12//PSME1//RFC5//CCNB1//CCNB2//CCNE2//H2AFX//CTNNB1//BPTF//Z
RAD51AP1//H2AFX//RAD51//RNASEH1//HMGB2//CCNE1//CCNE2//ESCO2//CCDC88A//STRA13//RDM1//EYA2//U
H2AFX
GAPDHS//BPGM//TPI1//IDH3A//RAB31//GCH1//NKIRAS1//TRIM23//RHOB//RAB22A//RAB3A//RAB5A//RRAGC//RA
RSF10//NCBP2//GLE1//UPF3B//U2AF1//SRSF9//KPNA2//IPO11//RPL23//SMURF1//STRADA//SDCBP//RTP4//TOM
//PSMD11//PSMD12//PSME1//RFC5//SKP1
/IFNG//IGF1//FBXW11//EIF5A2//ADNP//VEGFA//UBE2C//PSMA8//ANAPC11//PSMA3//PSMA6//PSMA7//PSMB5//P

SMD12//PSME1

MD9//PSMD11//PSMD12//PSME1//EEF1E1//GRB2
A//CALU//CD9//HNRNPA1//NXF1//SRSF10//NCBP2//GLE1//UPF3B//U2AF1//SRSF9//KPNA2//IPO11//RPL23//SMUR

P5//CENPI//H2BFM//H2AFX//H2AFZ//HMGB2//NAP1L4//NPM1//H2AFJ//CENPH//HIST2H2AC//H2AFV//SBDS//RPS
T2H2AC//H2AFV//BAZ1B//CHD1

RAB3A//MYC//CCDC88A//CCNB1//SBDS//EGF//IGF1//IL1B//NUSAP1//DLGAP5//DYNC1LI1//TPM1//CYLD

AFX//H2AFZ//HMGB2//NAP1L4//NPM1//H2AFJ//CASC5//CENPH//HIST2H2AC//H2AFV//SRSF9//UBE2C//UBE2S//
2//VEGFA//TBK1//PELI2//YWHAZ//HSPA1B//HSPA6//HSPA8//HSP90AA1//HLA-B//HP//INHBA//CAMLG//PPBP//EGF
5//RPS24//RPL23//CTNNB1//MST4//STK24//STMN1//KIF18A//TOP2A//SBDS//CCNB1//EIF5A2
AFX//H2AFZ//HMGB2//NAP1L4//NPM1//H2AFJ//CASC5//CENPH//HIST2H2AC//H2AFV//BAZ1B//SRSF9//RPL27A//
2//PLA2G4C//PPM1D//WASL//MTDH//DCN//GCH1//NCF2//AKIRIN2//BDKRB1//SOCS1//NXF1//SYNCRIP//CENPA//
051//CASC5//CENPA//OIP5//CENPI//H2BFM//H2AFX//H2AFZ//HMGB2//NAP1L4//NPM1//H2AFJ//CENPH//HIST2H2
1//FBXW11//EIF5A2//ADNP//VEGFA//UBE2C//PSMA8//ANAPC11//PSMA3//PSMA6//PSMA7//PSMB5//PSMC5//PSM

//STK24//STMN1//KIF18A//TOP2A//SBDS//CCNB1//EIF5A2
PSMA8//ANAPC11//PSMA3//PSMA6//PSMA7//PSMB5//PSMC5//PSMD9//PSMD11//PSMD12//PSME1//SKP1//UBE
/H2BFM//H2AFX//H2AFZ//HMGB2//NAP1L4//NPM1//H2AFJ//CENPH//HIST2H2AC//H2AFV//SRSF9//GCH1//MDM2

6//PSMA7//PSMB5//PSMC5//PSMD9//PSMD11//PSMD12//PSME1
CCDC88A//SOCS1//BDKRB1//CCNB1//PELI2//PUM2//NGDN//TACO1//RPS14//NCBP2//EIF4H//EIF5A2//AKT1S1//D

E2D3//MDM2//HERC6//SMURF2//CCDC88A//SOCS1//BDKRB1//CCNB1//PELI2//EIF5A2//FEM1B//DCAF7//MARCH
LE2//BCCIP//RAD51C//RFC5//TOP2A//UBE2D3//PTTG1//MORF4L2//HMGB2//SOD1//BAZ1B//TRIP13//UBR5//PSM

12//CAP2//SSTR2//PRKACB//BDKRB1//DAB2IP//CYCS//GH1//TFPI//CAST//NPM1//HMGB2//PCNA//ADNP//STRAD

SOCS1//BDKRB1//CCNB1//PELI2//AKT1S1//DAB2IP//GH1//ADNP//UBQLN1//SMURF1//NLRP12//STRADA//IFNG//

FZ//HMGB2//NAP1L4//NPM1//H2AFJ//CASC5//CENPH//HIST2H2AC//H2AFV//SRSF9//GCH1//MDM2//PARD6B//CC
1B//GCH1//TFPT//INHBA//WWOX//FOXL2//TPD52L1//IFI27//NLRP12//NGFRAP1//CRH//CYCS//HBB//HP//PSMA8//

H3//HNRNPK//UPF3B//U2AF1//SRSF9//EIF4A3//WBP4//JMJD6//BCAS2
GFBR1//ARPP19//SBDS//EIF4A3//ADNP//NPPC//SMURF1//CCNB1//MDM2//IFNG//EGF//SDCBP//BPTF//FBXW11/
P12//CAP2//SSTR2//PRKACB//BDKRB1//DAB2IP//CYCS//GH1//TFPI//CAST//NPM1//HMGB2//PCNA//CYLD//ADNP

/HMGB2//PCNA//CTHRC1//ADNP//STRADA//CCDC88A//TPM1//CCL26//GCLM//FOXL2//NME1//TXN//TPD52L1//G
//NAE1//CDKN3//CKS2//SERTAD1//BCCIP//CCNE2//CCNE1//CDO1//CTNNB1//BPTF//ZFPM2//SUZ12//FOSB//HSE

TFPT//INHBA//WWOX//FOXL2//TPD52L1//IFI27//NLRP12//NGFRAP1//CRH//CYCS//IFNG//CTNNB1//DAB2IP//GAL/

//SUN1//MDM2

//SP110//TRIM23//KPNA2//MDM2//NFX1//NPM1//PSMA3//PSMA7//PSMB5//BUB1//TOP1//EIF4H//PDCL3//ISG15
VHAZ//FEM1B//GCH1//TFPT//INHBA//WWOX//FOXL2//TPD52L1//IFI27//NLRP12//NGFRAP1//CRH//CYCS//PSMA8

EGF//ORC6//MCM10//RRM2//PAPD5
//HERC6//SMURF2//CCDC88A//SOCS1//BDKRB1//CCNB1//PELI2//EIF5A2//FEM1B//DCAF7//MARCH6//UBE2C//D
HNRNPM//NCBP2//SYNCRIP//LSM3//HNRNPA1//HNRNPH3//HNRNPK//UPF3B//U2AF1//SRSF9//EIF4A3//WBP4//J
M3//HNRNPA1//HNRNPH3//HNRNPK//UPF3B//U2AF1//EIF4A3
/SOCS1//BDKRB1//CCNB1//PELI2//PUM2//NGDN//TACO1//RPS14//NCBP2//EIF4H//EIF5A2//AKT1S1//DAB2IP//GI
P//IL1B//CYLD//FBXW11//HSP90AA1//SEC61G//TGFBR1
/TGFBR1
GCH1//TFPT//INHBA//WWOX//BNIP1//FOXL2//TPD52L1//PSMA8//MDM2//TRIAP1//PSMA3//PSMA6//PSMA7//PSM

AE1//CDKN3//CKS2//SERTAD1//BCCIP//CCNE2//CCNE1//CTNNB1//BPTF//ZFPM2//SUZ12//FOSB//HSBP1//MDM2

TNNB1//TOB1//NLRP12//NDC80//SGOL1//CASC5//KIF20A//CENPF//RAB31//SNF8//CHMP4B//CHCHD4//VPS13B//
/STRADA//SDCBP//RTP4//TOMM5//YWHAZ//PCNA//SYTL2//STX17//RIMS2//CTNNB1//TOB1//NLRP12//NDC80//S

-

PP19//SBDS//EIF4A3//ADNP//NPPC//SMURF1//CCNB1//MDM2//IFNG//EGF//SDCBP//BPTF//FBXW11//HNRNPAB

//HMGB2//PCNA//ADNP//STRADA//CCDC88A//TPM1//CCL26//GCLM//FOXL2//TPD52L1//GCH1//UBE2C//PSMA8//
IMU//PPM1A//HMGXB4//WWOX//SMURF1//FOXD4L1//FOXD4//FOXD2//FOXD4L3//TWSG1//TOB1//VEGFA//RAB3

3//CYLD//FBXW11//HSP90AA1//SEC61G//TGFBR1//CENPA//SUN1
J2AF1//SRSF9//EIF4A3//WBP4//JMJD6//SNRNP48//SREK1IP1//TXNL4B//RRAGC

1//BDKRB1//CCNB1//SIRT4//DAB2IP//FOXD4L1//FOXD4//FOXD2//FOXD4L3//IFNG//FOXL2//WNT9A//ADNP//TBK
H//TCEAL1//BDKRB1//CCNB1//SIRT4//DAB2IP//FOXD4L1//FOXD4//FOXD2//FOXD4L3//IFNG//FOXL2//WNT9A//A

RPL7L1//GTF2H3//ASMT//MRPS7//MRPS23//BRIX1//MRPL47//MRPL12//MRPS25//SECISBP2//MRPL33
B1//NLRP12//TXN//UBR5//EGF//DAB2IP//IL1B//CYLD//FBXW11//HSP90AA1//SEC61G//DPH3//PANX1//TGFBR1//K
E2D3//ISG15//MDM2//HERC6//SMURF2//CCDC88A//SOCS1//BDKRB1//CCNB1//PELI2//CCT7//DNAJC21//HSPA8/

2//STX17//RIMS2//TOB1//NLRP12//KIF20A//CENPF//RAB31//SNF8//CHMP4B//CHCHD4//VPS13B//SEC22A//GLE
/NPM1//SDAD1

CCNB1//CCNB2

SDCBP//RTP4//TOMM5//YWHAZ//HSP90AA1//NPM1//PCNA//SYTL2//STX17//RIMS2//SEC22A//TRAPPC2//GOSR

SME1//ARPP19//STRADA

//NLRP12
NPM1//ABI1//WASL//SGOL1//CASC5//CCL26//SKA2//NUSAP1//SBDS//CCNB1//TPM1//DYNC1LI1//HAUS2//NEK2/

RPS24//UTP23//EIF4A3//SRSF9//NPM1//SDAD1

)H//TCEAL1//BDKRB1//CCNB1//SIRT4//DAB2IP//FOXD4L1//FOXD4//FOXD2//FOXD4L3//IFNG//FOXL2//WNT9A//C

/H2AFV//BPTF//USP16//EYA2//EZH2//KDM2A//JMJD6//SUZ12//ING3//ENY2//HIRA//MORF4L2//CCNB1//POLE4

11//PSMA3//PSMA6//PSMA7//PSMB5//PSMC5//PSMD9//PSMD11//PSMD12//PSME1

/SBDS//EIF4A3//SMURF1//CCNB1//MDM2//IFNG//BPTF//FBXW11//HNRNPAB//PSMC5//TBK1//H2AFX//GAPDHS/

AZI2
F//ZFPM2//GGCX//GRB2//HBB//IGF1//ITGA2B//ITGAM//KIF11//RHOB//LAMP2//PPBP//PRKACB//PTPN1//RAB5A//F

OXD4L3//TWSG1
0//RFC5//RRM2//PAPD5//TOP1//TOP2A//NAE1//CCNE1//CTNNB1//BPTF//ZFPM2//SUZ12//FOSB//HSBP1//MDM2
)AC3//EIF5A2//ENY2//EIF4A3

-//RPS15//RPS24//RPL23//VMO1//CENPI//INHBA//SOX3//CTNNB1//NCBP2//GTF2H3//FEM1B//HOXB13

3//PSMA6//PSMA7//PSMB5//PSMC5//PSMD9//PSMD11//PSMD12//PSME1
RSF9//EIF4A3//GTF2H3//CCNB1//WBP4//JMJD6//CDK11A//BCAS2//SNRNP48//SREK1IP1//TXNL4B

TNNB1//EGF//BAZ1B//FEM1B//GCH1//TFPT//INHBA//WWOX//BNIP1//FOXL2//TPD52L1//CD55//PSMA8//MDM2//TI
NPM1//BCCIP//RAD51C//TOP2A//UBE2D3//PTTG1//MORF4L2
GF//DAB2IP//CYLD//FBXW11//SIRT4//NCBP2//DPH3//PANX1//CRH//CCNB1//TGFBR1//CTNNB1

4

RIMS2//TOB1//NLRP12//TXN//UBR5//CTNNB1//MDM2//EGF//DAB2IP//IL1B//CYLD//FBXW11//HSP90AA1//SEC61G

4//FOXD2//FOXD4L3//TWSG1//TOB1//INHBA

MS2//TOB1//NLRP12//TXN//UBR5//CTNNB1//MDM2//RRAGB//RRAGC//EGF//DAB2IP//IL1B//CYLD//FBXW11//HSP

CNB1//PELI2//UQCRB//NDUFA8//NDUFB2//NDUFB4//NDUFB9//NDUFS6//NME1//CAMKK2//TP53RK//TBK1//MST4

CNB1//PELI2//UQCRB//NDUFA8//NDUFB2//NDUFB4//NDUFB9//NDUFS6//NME1//CAMKK2//TP53RK//TBK1//MST4

//SOCS1//CCNB1//CCNE1

RRAGB//RRAGC//IL1B//IFNG//CTNNB1//TBK1//MAP3K3//ZDHHC13//PELI2//BST2//MTDH//EEF1E1//WNT9A//SDC

)1//CALU//CD9//BDKRB1//SLC6A6//KIF20A//VAMP5//SEC22A//SYTL2//STX17//TRAPPC2//BNIP1//GOSR1//TPD52

/VEGFA//RRAGB//RRAGC//IFNG//IL1B//CTNNB1//TBK1//MAP3K3//ZDHHC13//PELI2//BST2//MTDH//EEF1E1//WN

AGB//RRAGC//IFNG//IL1B//CTNNB1//TBK1//MAP3K3//ZDHHC13//PELI2//BST2//MTDH//EEF1E1//WNT9A//SDCBP

SLC6A6//KIF20A//VAMP5//SEC22A//SYTL2//STX17//TRAPPC2//BNIP1//GOSR1//TPD52//CA2//HNRNPA1//NXF1//S

GF//DAB2IP//IL1B//CYLD//FBXW11//HSP90AA1//SEC61G//TGFBR1//NPM1//PCNA//SYTL2//STX17//RIMS2

12//CCNB1//INHBA//CTNNB1//MYC//FOXD4L1//FOXD4//FOXD2//FOXD4L3

C1//STMN1//KIF18A//UBE2C//UBE2S//CCL26//NDUFAF4//PANX1//HSP90AA1//STOM//RAD51//SBDS//POMP//PSM

4L1//FOXD4//FOXD2//FOXD4L3//TWSG1//INHBA

LC16A1//SOD1//TFPI//VEGFA//YWHAZ//TFPI2//CALU//KIF18A//CD9//KIF23//PLSCR1//CCNB1//DCN//IL1B//SDC2

M//HSPA1B//HSPA6//HSPA8//HSP90AA1//GCLM//MAT2B//MAT2A//CCL26//CMTM8//KAL1//PPBP//CMTM6//CCL7

2M

2L1//ANAPC11//PSMA3//PSMA6//PSMA7//PSMB5//PSMC5//PSMD9//PSMD11//PSMD12//PSME1

35//PSMC5//PSMD9//PSMD11//PSMD12//PSME1//SSTR2//PRKACB//STK3//NKIRAS1//TBK1//AZI2//CRKL//DAB2I

TOP2A//NAE1//CCNE1//CDO1//CTNNB1//BPTF//ZFPM2//SUZ12//FOSB//HSBP1//MDM2//NFX1//RPS14//TXN//MT

E1//CCNE1//CTNNB1//BPTF//ZFPM2//SUZ12//FOSB//HSBP1//MDM2//NFX1//RPS14//TXN//MTDH//TCEAL1//DCP
SMA7//PSMB5//PSMC5//PSMD9//PSMD11//PSMD12//PSME1//ZNF287//SKP1
ITGAM//KIF11//PRKACB//PTPN1//RAB5A//RAD51C//SLC16A1//TFPI2//KIF18A//KIF23

/CKS2//SERTAD1//BCCIP//CCNE2//CCNE1//CTNNB1//BPTF//ZFPM2//SUZ12//FOSB//HSBP1//MDM2//NFX1//RPS
N//WNT9A//STC2//GDF15//ISG15//DLGAP5//UTS2//CRH//PANX1//HSPA8//MDM2//PRKACB//RAB3A//SDCBP//KI
NME1//NPM1//NPPC//NOX4//RARRES3//SOD2//SSTR2//WNT9A//PPM1D//CD9//EEF1E1
D1//TFPI//VEGFA//YWHAZ//TFPI2//CALU//KIF18A//CD9//KIF23//PLSCR1

D1//TFPI//VEGFA//YWHAZ//TFPI2//CALU//KIF18A//CD9//KIF23//PLSCR1

2A//ADNP//SLC30A8//NCF2//CDO1//ALDOC//IL1B//CCNE1//LUM//STC1//IFI6//HLA-B//IFI27//IFI35//IFNG//OAS1//O

'SMD11//PSMD12//PSME1//RFC5//CCNB1//CCNB2//CCNE2//H2AFX//CDKN3//CKS2//SERTAD1//BCCIP//PCNA//R
C//CCNB1//BPTF//FBXW11//HNRNPAB//PSMC5//HBB//HSP90AA1//IFNG//H2AFX//USP16//PPM1A//ENY2//MRPL1

ACB//PTPN1//RAB5A//RAD51C//SLC16A1//SOD1//TFPI//VEGFA//YWHAZ//TFPI2//CALU//KIF18A//CD9//KIF23//CD

SMA8//RFC5//CENPF//KPNA2//CEP152//BIRC5//HSP90AA1//HAUS2//TPD52L1//PPM1D//CCNB2//KIF20A//CENP
IE2//H2AFX//CDKN3//INHBA//PCNA//POLE2//RRM2//SKP1//CCNE1//CENPF//CEP152//BIRC5//HSP90AA1//HAUS
FX//CDKN3//CKS2//SERTAD1//BCCIP//INHBA//PCNA//POLE2//RRM2//SKP1//CCNE1//CENPF//CEP152//BIRC5//H
CNE2//PSMA8//RFC5//CENPF//CEP152//BIRC5//HSP90AA1//HAUS2//TPD52L1//PPM1D//CCNB2//KIF20A//CEN
AURKA//STMN1//USP16//OIP5//CDCA2//DYNC1LI1//ANAPC11//TXNL4B//HAUS2//PBK//PAPD5//DYNLT3//CDK11
P//TRIAP1//SESN3//IFNG//INHBA//MYC//PPM1A//RPRM//STRADA//BUB1//NEK2//CDK11A//ZWINT//KNTC1//CEN
/CDK11A//ZWINT//KNTC1//CENPF//NPM1//BIRC5//NAE1//WNT9A//STAG2//SBDS//EGF//IGF1//IL1B//NUSAP1//DL
//DYNC1LI1//ANAPC11//TXNL4B//HAUS2//PBK//PAPD5//AURKA//DYNLT3//CDK11A//FAM83D//CCNB2//PTTG1//K
PC11//HAUS2//CASC5//SPC25//PAPD5//CENPH//AURKA//BUB1//DYNLT3//FAM83D//PARD6B//CDC14A//CCNB1
A//SBDS//CCNB1//EGF//IGF1//IL1B//NUSAP1//DLGAP5//BTG3//TOP2A//DYNC1LI1//CYLD//RPRM//DYNLT3
BK//PAPD5//AURKA//DYNLT3//CDK11A//FAM83D//CCNB2//PTTG1//KIF23//KLHL21//DLGAP5//EGF//IGF1//IL1B
1LI1//TXNL4B//HAUS2//PBK//PAPD5//AURKA//DYNLT3//FAM83D//CCNB2//PTTG1//KIF23//KLHL21
NC1LI1//FEM1B//ORC6//MCM10//RFC5//CCNB2//CCNE2
K//PAPD5//AURKA//DYNLT3//CDK11A//FAM83D//CCNB2//PTTG1//KIF23//KLHL21//DLGAP5//EGF//IGF1//IL1B
1//KNTC1//CENPF//NAE1//WNT9A//BIRC5//TOP2A//CASC5//DYNC1LI1//FEM1B
E1//WNT9A//BIRC5//TOP2A//CASC5//CDKN3//SESN3//IFNG//INHBA//MYC//PPM1A//RPRM//STRADA

SN3//IFNG//INHBA//MYC//PPM1A//RPRM//STRADA//ZWINT//BUB1//KNTC1//CENPF//NAE1//WNT9A//BIRC5//BTG

152//BIRC5//HSP90AA1//NEK2//HAUS2//TPD52L1//PPM1D//CCNB2//MDM2//WNT9A//TOP2A

JBE2C//CYLD//UBR2//PSMD9//SKP1//UBE2D2//UBE2D3//UBE2G2//USP13//NPLOC4//UBE2S//MAD2L1//ANAPC1
D52L1//PPM1D//CCNB2//MDM2//WNT9A//TOP2A

IA//POLE2//RFC5//TOP2A//IFI6//SOD2//CHD1//BAZ1B//OIP5//H2BFM//H2AFX//H2AFZ//HMGB2//NAP1L4//NPM1//

PH//BUB1//KIF18A//CCNB1//KNTC1//SRSF10//SMN2//SNRPD1//SNRPD2//NCBP2//PCNA//POLE2//RFC5//RAD51
NPI//BIRC5//CASC5//SPC25//CENPH//BUB1//KIF18A//CCNB1//KNTC1//SRSF10//SMN2//SNRPD1//SNRPD2//NCE

1//FEM1B//MARCH6//UBE2D2//UBE2G2//DCAF7//DCAF4//TRIM23//PCNP//KLHL21

/UBE2S//UBE2T//MAD2L1//PSMA3//PSMA6//PSMA7//PSMB5//PSMC5//PSMD9//PSMD11//PSMD12//PSME1//PSM
UZ12//USP16//ENY2//NAE1//UBQLN1//UBE2S//UBE2T//MAD2L1//PSMA3//PSMA6//PSMA7//PSMB5//PSMC5//PS

UBE2D3

C5//PSMD11//PSMD12//PSME1//AURKA//CCNB1//PTTG1//FBXW11//PCNP//ISG15
5//PSMC5//PSMD11//PSMD12//PSME1//AURKA//CCNB1//PTTG1//FBXW11//PCNP

XW11//PCNP//UBE2D3//USP16//CYLD//UBR2//UBE2D2//UBE2G2//USP13
3E2S//MAD2L1//ANAPC11//PSMB5//PSMC5//PSMD11//PSMD12//PSME1//AURKA//CCNB1//PTTG1//FBXW11//PCN

C//BUB1//DLGAP5//KIF18A//CCNB1//SYCP2//USP16//EYA2//KDM2A//JMJD6//SUZ12//ING3//ENY2//HIRA//MORF4
1//PSMC5//PSMD11//PSMD12//PSME1//AURKA//CCNB1//PTTG1//FBXW11//PCNP
D//UBR2//PSMD9//SKP1//UBE2D2//UBE2D3//UBE2G2//USP13//NGLY1//PSMC5//NPLOC4//UBE2S//MAD2L1//AN

NTC1//SRSF10//SMN2//SNRPD1//SNRPD2//NCBP2//PCNA//POLE2//RFC5//RAD51//TOP2A//CAP2//NOX4//RRA

IPH//BUB1//KIF18A//CCNB1//KNTC1//SRSF10//SMN2//SNRPD1//SNRPD2//NCBP2//PCNA//POLE2//RFC5//RAD5

SMD11//PSMD12//PSME1//AURKA//CCNB1//PTTG1//FBXW11//PCNP//IFNG//IL1B

LD

B//PSMC5//HMGB2//CYCS//SOD1//FOXL2//TPD52L1//RNASEH1//HSPA1B//PSMA8//PSMA3//PSMA6//PSMA7//PS

/RPL27A//RPS7//RPS24//RPL23//STMN1//KIF18A//ABI1//WASL//UBE2C//UBE2S//CCL26//NDUFAF4//HSP90AA1//

IB5//PSMC5//PSMD9//PSMD11//PSMD12//PSME1//RRAGC//BNIP1//BUB1//CDK11A//UBE2D3//PDCL3//SLTM//STK
FPM2//SUZ12//FOSB//HSBP1//NFX1//RPS14//TXN//MTDH//TCEAL1//BDKRB1//NME1//IFNG//SIRT4//CFDP1//IFI6
5//PSMD9//PSMD11//PSMD12//PSME1//RRAGC//BNIP1//BUB1//CDK11A//UBE2D3//PDCL3//SLTM//STK24//SOD1/

;MA7//PSMB5//PSMC5//PSMD9//PSMD11//PSMD12//PSME1//RRAGC//BNIP1//BUB1//CDK11A//UBE2D3//PDCL3/
21//HSPA8//NKTR//FKBP14//DNAJA4//HSPA1B//HSP90AA1//EIF5A2//FEM1B//DCAF7//MARCH6//UBE2C//DCAF4/

:NB1//CDK11A//PAPD5//PSMA8//HSPA8//PSMA3//PSMA6//PSMA7//PSMB5//PSMC5//PSMD9//PSMD11//PSMD12/
NME1//PSMA3//PSMA6//PSMA7//PSMB5//PSMC5//PSMD9//PSMD11//PSMD12//PSME1//BEX2//NAE1//TOP2A//IF

1//PTTG2//ZWINT//MAD2L1//NUSAP1//UBE2C//PSMA8//MDM2//ANAPC11//PSMA3//PSMA6//PSMA7//PSMB5//PSI
TRIAP1//PSMA3//PSMA6//PSMA7//PSMB5//PSMC5//PSMD9//PSMD11//PSMD12//PSME1//GRB2//EEF1E1//FEM1E

FPM2//SUZ12//FOSB//HSBP1//NFX1//RPS14//TXN//MTDH//TCEAL1//BDKRB1//NME1//IFNG//SIRT4//CFDP1//IFI6/
JBE2T//NPM1//BCCIP//RAD51C//UBE2D3//PTTG1//MORF4L2//SOD1//BAZ1B//TRIP13//CYCS//FOXL2//TPD52L1//

AB7L1//RAB33A//KIF1B//PSMC5//HMGB2//CYCS//SOD1//FOXL2//TPD52L1//RNASEH1//HSPA1B//PSMA8//PSMA3
M5//HSP90AA1//NPM1//PCNA//SYTL2//STX17//RIMS2//SEC22A//TRAPPC2//GOSR1//HSPA8//CTNNB1//DLGAP5/

SMC5//PSMD9//PSMD11//PSMD12//PSME1//SKP1//UBE2S

RF1//STRADA//SDCBP//RTP4//TOMM5//HSP90AA1//NPM1//PCNA//SYTL2//STX17//RIMS2//SEC22A//TRAPPC2//G

7//RPS24//UTP23//EIF4A3//SRSF9//GCH1//MDM2//PARD6B//CCNB1//WASL//REPS2//KNTC1//SDCBP//SYCP2//U

CCL26//STMN1//NDUFAF4//HSP90AA1//POMP//PSMG1//CENPF//PSMD9
F//ZFPM2//GGCX//GRB2//HBB//IGF1//ITGA2B//ITGAM//KIF11//RHOB//LAMP2//PRKACB//PTPN1//RAB5A//RAD51C

RPS7//RPS24//RPL23//GCH1//MDM2//PARD6B//CCNB1//WASL//REPS2//KNTC1//STMN1//KIF18A//ABI1//UBE2C/
MAGI3//GRB2//ANPEP//HLA-B//HNRNPA1//HNRNPK//HSPA8//SP110//TRIM23//KPNA2//MDM2//NFX1//NPM1//PSI
AC//H2AFV//SRSF9//SDCBP//PARD6B//SYCP2//UBE2C//UBE2S//CCDC88A//VMO1//CCL26//CD9//STMN1//NDUF
MD9//PSMD11//PSMD12//PSME1//SKP1//UBE2S

2S
//PARD6B//CCNB1//WASL//REPS2//KNTC1//SDCBP//SYCP2//UBE2C//UBE2S//CCDC88A//VMO1//CCL26//CD9//S

)AB2IP//GH1//ADNP//SBDS//EIF4A3//UBQLN1//SMURF1//NLRP12//STRADA//MDM2//IFNG//PRKACB//IGF1//TPD5

I6//UBE2C//DCAF4//TRIM23//ANAPC11//PCNP//SKP1//UBE2D2//KLHL21//CAMKK2//TP53RK//TBK1//MST4//SNRI
IA8//MDM2//TRIAP1//PSMA3//PSMA6//PSMA7//PSMB5//PSMC5//PSMD9//PSMD11//PSMD12//PSME1//CRKL//DA

A//CCDC88A//ACAP2//TBC1D2B//TNNI3//TPM1//CCL26//GCLM//IFI6//BIRC5//FOXL2//NGFRAP1//TPD52L1//PPP

PRKACB//IGF1//TPD52L1//CAMKK2//VEGFA//UBE2C//MAD2L1//ANAPC11//PSMA3//PSMA6//PSMA7//PSMB5//PS

NB1//WASL//REPS2//KNTC1//UBE2C//UBE2S//CCL26//STMN1//NDUFAF4//PANX1//HSP90AA1//STOM//POMP//F
NME1//MST4//PSMA3//PSMA6//PSMA7//PSMB5//PSMC5//PSMD9//PSMD11//PSMD12//PSME1//BEX2//NAE1//IFN

HNRNPAB//PSMC5//TBK1//HBB//HSP90AA1//H2AFX//GAPDHS//USP16//PPM1A//ENY2//MRPL12//FOXL2//CCNE
//CTHRC1//STRADA//CCDC88A//ACAP2//TBC1D2B//TNNI3//TPM1//CCL26//GCLM//IFI6//BIRC5//FOXL2//NGFRAI

CH1//RHEBL1//NPM1//PSMA6//MTDH//DPH3//UBE2C//PSMA8//ANAPC11//PSMA3//PSMA7//PSMB5//PSMC5//PS
P1//MDM2//NFX1//RPS14//TXN//MTDH//TCEAL1//DCP2//NCBP2//UPF3B//EIF4A3//TGFBR1//IL1B//SOD1//SPRED

ING3//STK3//TOP2A//EEF1E1//MAD2L1//MDM2//SOD2//VEGFA//MTDH//ADNP//GCLM//NCF2//AKT1S1//TGFBR1

//NME1//MST4//PSMA3//PSMA6//PSMA7//PSMB5//PSMC5//PSMD9//PSMD11//PSMD12//PSME1//BEX2//NAE1//IF

CAF4//TRIM23//ANAPC11//PCNP//SKP1//UBE2D2//KLHL21//GGCX//UBE2S//CAMKK2//TP53RK//TBK1//MST4//S
JMJD6

H1//ADNP//SBDS//EIF4A3//UBQLN1//SMURF1//NLRP12//STRADA//MDM2//IFNG//PRKACB//MAD2L1//IGF1//TPD

B5//PSMC5//PSMD9//PSMD11//PSMD12//PSME1//SUZ12//SERTAD1//IGF1//MAB21L1//AKIRIN2//EIF5A2//TGFBR

//NFX1//RPS14//TXN//MTDH//TCEAL1//DCP2//NCBP2//UPF3B//EIF4A3//TGFBR1//IL1B//SOD1//SPRED2//FBXW1

/SEC22A//GLE1//RABGEF1//RAB40AL//RAB37//SERP2//RHOB//SDAD1//EIF5A2//RAB22A//RAB3A//RAB5A//KIF1[illegible]
GOL1//CASC5//KIF20A//CENPF//RAB31//SNF8//CHMP4B//CHCHD4//SEC22A//RABGEF1//RAB40AL//RAB37//SE[illegible]

//PSMC5//TBK1//HBB//HSP90AA1//H2AFX//GAPDHS//USP16//PPM1A//ENY2//MRPL12//FOXL2//CCNE1//EIF5A2/

/ANAPC11//PSMA3//PSMA6//PSMA7//PSMB5//PSMC5//PSMD9//PSMD11//PSMD12//PSME1//SKP1//CCNB1//UBE
A//AKT1S1//RRAGB//RRAGC//SLC30A8//PSMD9//GRB2//EPN1//SOCS1//TBK1//MAP3K3//ZDHHC13//PELI2//BST

1//CENPF//CCDC85B//EZH2//HMGB2//AKIRIN2//PSMC5//SMURF2//HIRA//EIF4A3//NCBP2//NAE1//NLRP12//FBX
DNP//TBK1//CENPF//CCDC85B//EZH2//HMGB2//AKIRIN2//PSMC5//SMURF2//HIRA//EIF4A3//NCBP2//NAE1//NL[illegible]

IF20A//CENPF//RAB31//SNF8//CHMP4B//CHCHD4//VPS13B//SEC22A//GLE1//RABGEF1//RAB40AL//RAB37//SE[illegible]
/NKTR//FKBP14//DNAJA4//HSPA1B//HSP90AA1//EIF5A2//FEM1B//DCAF7//MARCH6//UBE2C//DCAF4//TRIM23//[illegible]

1//RABGEF1//RAB40AL//RAB37//SERP2//RHOB//SDAD1//EIF5A2//RAB22A//RAB3A//RAB5A//KIF18A//RAB7L1//C

1//HSPA8//TOB1//KIF1B//HMGXB4//RHOB//NLRP12//TXN//UBR5//EGF//DAB2IP//IL1B//CYLD//FBXW11//ATP5G1//[illegible]

/SOD1//CYLD//SUN1//CAP2//SGCD

:ENPF//CCDC85B//EZH2//HMGB2//AKIRIN2//PSMC5//SMURF2//HIRA//EIF4A3//NAE1//NLRP12//FBXW11//STAG2

/USP16//PPM1A//ENY2//MRPL12//FOXL2//CCNE1//EIF5A2//PQBP1//CAMKK2//INHBA//SMAD5//MAX//MYBL1//MY

RAD51C//SLC16A1//TFPI//VEGFA//TFPI2//CALU//KIF18A//CD9//KIF23//TXN//HSD17B3//HSD17B6//CRH//CCL7//ST

//NFX1//RPS14//TXN//MTDH//TCEAL1//DCP2//NCBP2//UPF3B//EIF4A3//SRSF10//SMN2//SNRPD1//SNRPD2//LSM

RIAP1//PSMA3//PSMA6//PSMA7//PSMB5//PSMC5//PSMD9//PSMD11//PSMD12//PSME1//SUZ12//SERTAD1//IGF1

//TGFBR1//CENPA//SUN1//RRAGB//RRAGC

?90AA1//SEC61G//TGFBR1//CENPA//SUN1
4//SNRK//PRKACB//PBK//EIF2AK2//TEX14//STK3//AURKA//AURKC//BUB1//CDK11A//STK24//CCNE1//AKT1S1//PI
4//SNRK//PRKACB//PBK//EIF2AK2//TEX14//STK3//AURKA//AURKC//BUB1//CDK11A//STK24//CCNE1//AKT1S1//PI

CBP//TPD52L1//UBR5//DAB2IP
2//CA2//VPS13B//NPM1//HNRNPA1//NXF1//SRSF10//NCBP2//GLE1//UPF3B//U2AF1//SRSF9//KPNA2//IPO11//RPL

IT9A//SDCBP//TPD52L1//NMU//UBR5

?//TPD52L1//NMU//UBR5

SRSF10//NCBP2//GLE1//UPF3B//U2AF1//SRSF9//KPNA2//IPO11//RPL23//SMURF1//STRADA//SDCBP//RTP4//TON

IG1//EIF5A2//NPM1//RRM2//USP16//ALDOC//SOD2//PDSS2//HBB//CENPA//CENPF//PSMD9

//TPM1

'/NDUFB4//CAP2//GRB2//ITGA2B//RHOB//SDCBP//WASL//ENSA//SSTR2//STC1//STC2//CCNB1//BAIAP2//PPM1A

P//GH1//SOCS1//RAB31//CHN1//TAGAP//RHEBL1//RAB40AL//RAB37//TRIM23//RHOB//RHOT1//DEPDC1B//RAB2

TDH//TCEAL1//DCP2//NCBP2//UPF3B//EIF4A3//SRSF10//SMN2//SNRPD1//SNRPD2//LSM3//BCAS2//HNRNPM//S

2//NCBP2//UPF3B//EIF4A3//SRSF10//SMN2//SNRPD1//SNRPD2//LSM3//BCAS2//HNRNPM//SYNCRIP//HNRNPA

14//TXN//MTDH//TCEAL1//DCP2//NCBP2//UPF3B//EIF4A3//TGFBR1//IL1B//SOD1//SPRED2//FBXW11//UBR5//SM

F1B//SYT9//IFNG//SLC2A1//DAB2IP//NLRP12//TGFBR1//GH1//IGF1//NMU//SOD1//SLC30A8//GAL//FAM3B//PPM1

AS2//PTPN1//SOCS1//ISG15//SDC2//MTDH//CTNNB1//GH1//AURKA//DCN//GCH1//AKIRIN2//BDKRB1//CLDN4//G

RM2//CCNE1//CTNNB1//BPTF//ZFPM2//SUZ12//FOSB//HSBP1//NFX1//RPS14//TXN//MTDH//TCEAL1//CAMKK2//
12//FOXL2//CCNE1//PQBP1//CAMKK2//INHBA//SMAD5//MAX//MYBL1//MYC//NPM1//PSMD9//TGFBR1//ZFX//MED

O1//NME1//PLSCR1//PDSS2

\/STAG2//SGOL1//CENPI//SKA2//CASC5//SPC25//CENPH//BUB1//KIF18A//KNTC1//DLGAP5//KIF11//STMN1//USI
2//TPD52L1//PPM1D//KIF20A//CENPA//STAG2//SGOL1//CENPI//SKA2//CASC5//SPC25//CENPH//BUB1//KIF18A//I
ISP90AA1//HAUS2//TPD52L1//PPM1D//KIF20A//CENPA//STAG2//SGOL1//CENPI//SKA2//CASC5//SPC25//CENPH
PA//STAG2//SGOL1//CENPI//SKA2//CASC5//SPC25//CENPH//BUB1//KIF18A//KNTC1//DLGAP5//TRIP13//TOP2A//U
\/FAM83D//CCNB2//PTTG1//KLHL21//H2AFX//RAD51//MND1//SYCP2//UBR2//UBE2S//SBDS//EGF//IGF1//IL1B
PF//CYLD//DYNLT3//NPM1//BIRC5//NAE1//WNT9A//STAG2//SBDS//PLA2G16//RHOB//EGF//IGF1//IL1B//NUSAP1/.
.GAP5//DYNC1LI1//CTNNB1//TOP2A//CASC5//FEM1B
KIF23//KLHL21//DLGAP5//UBE2S//EGF//IGF1//IL1B//KIF20A
//CCNE1//CCNB2//CCNE2//PTTG1//KNTC1

3//TOP2A//CASC5//DYNC1LI1//PLA2G16//RHOB

1//PSMC5//PSMD11//PSMD12//PSME1//AURKA//CCNB1//PTTG1//FBXW11//PCNP//PAPD5

H2AFJ//HIST2H2AC//H2AFV//SDCBP//CAP2//SGCD//TPM1//MAN2A1//RNASEH1//EZH2//BPTF//MRPL12//TOMM5

//TOP2A//IFI6//SOD2//CHD1//BAZ1B//OIP5//H2BFM//H2AFX//H2AFZ//HMGB2//NAP1L4//NPM1//H2AFJ//HIST2H2/
3P2//PCNA//POLE2//RFC5//RAD51//TOP2A//IFI6//SOD2//CHD1//BAZ1B//OIP5//H2BFM//H2AFX//H2AFZ//HMGB2//I

IA8//CCNB1//UBE2G2
MD9//PSMD11//PSMD12//PSME1//PSMA8//CCNB1//CYLD//UBE2G2

NP

4L2//MYC//POLE4//CENPF//CTNNB1//PTTG2//PTTG1

APC11//PSMD11//PSMD12//PSME1//AURKA//CCNB1//PTTG1//FBXW11//PCNP//IFNG//IL1B//PAPD5

GC//CDK11A//MORF4L2//IFI6//SOD2//CTNNB1//HNRNPAB//IFNG//CHD1//BAZ1B//OIP5//H2BFM//H2AFX//H2AFZ//

1//TOP2A//CAP2//NOX4//RRAGC//CDK11A//MORF4L2//IFI6//SOD2//CTNNB1//HNRNPAB//IFNG//CHD1//BAZ1B//O

MB5//USP16//UBE2C//CYLD//UBR2//PSMD9//SKP1//UBE2D2//UBE2D3//UBE2G2//USP13//MCCC2//SDS//MTDH/

SBDS//CCNB1//POMP//PSMG1//EIF5A2//CENPF//PSMD9

K24//SOD1//FOXL2//TPD52L1//CFDP1//HSPA1B//IGF1//NPM1//BCL2A1//YWHAZ//FEM1B//GCH1//TFPT//INHBA//
//HSPA1B//BIRC5//IGF1//IL1B//NPM1//TRIAP1//BCL2A1//BNIP1//SOD1//YWHAZ//CDKN3//SESN3//INHBA//MYC//
/FOXL2//TPD52L1//CFDP1//HSPA1B//IGF1//NPM1//BCL2A1//YWHAZ//FEM1B//GCH1//TFPT//INHBA//IFI27//NLRP

/SLTM//STK24//SOD1//FOXL2//TPD52L1//CFDP1//HSPA1B//IGF1//NPM1//BCL2A1//YWHAZ//FEM1B//GCH1//TFP
//TRIM23//ANAPC11//PCNP//SKP1//UBE2D2//KLHL21//CYLD//EIF1AX//RPL7L1//GTF2H3//ASMT//MRPS7//MRPS2

//PSME1//YWHAZ
NG//DAB2IP//GAL//ING3//STK3//EEF1E1//MAD2L1//MDM2//VEGFA//MTDH//ADNP//GCLM//NCF2//AKT1S1//TGFB

MC5//PSMD9//PSMD11//PSMD12//PSME1//CCNB1//CCNB2//CCNE2//H2AFX//CDKN3//CKS2//SERTAD1//BCCIP//
3//UBR5

//HSPA1B//BIRC5//IGF1//IL1B//NPM1//TRIAP1//BCL2A1//BNIP1//SOD1//YWHAZ//CDKN3//SESN3//INHBA//MYC//F
MND1//MYC//STAG2//NPPC//PTTG2

3//PSMA6//PSMA7//PSMB5//USP16//UBE2C//CYLD//UBR2//PSMD9//SKP1//UBE2D2//UBE2D3//UBE2G2//USP13//
//KIF18A//CCNB1//TOB1//RAB3A//SYT9//IL1B//PRKACB//SLC2A1//KIF1B//HMGXB4//RHOB//NLRP12//SGOL1//CA

GOSR1//HSPA8//DLGAP5//KIF18A//CCNB1//TOB1//RAB3A//SYT9//IL1B//PRKACB//SLC2A1//KIF1B//HMGXB4//RH

BE2C//UBE2S//CCDC88A//VMO1//CCL26//CD9//STMN1//NDUFAF4//PANX1//PVRL3//GRB2//LIMS1//CTNNB1//HS

://SLC16A1//TFPI//TFPI2//CALU//KIF18A//CD9//KIF23//STRA13//RDM1//EYA2//UBE2T//NPM1//PCNA//POLE2//BC

/UBE2S//CCL26//NDUFAF4//PANX1//HSP90AA1//STOM//SBDS//POMP//PSMG1//EIF5A2//RRM2//USP16//ALDOC
MA3//PSMA7//PSMB5//BUB1//TOP1//EIF4H//PDCL3//BST2//RSAD2//CMPK2//HMGB2
FAF4//PVRL3//GRB2//LIMS1//CTNNB1//HSP90AA1//BBIP1//POMP//PSMG1//TPM1//BAIAP2//HAUS2//CENPF//ADN

TMN1//NDUFAF4//PANX1//PVRL3//GRB2//LIMS1//CTNNB1//HSP90AA1//BBIP1//STOM//POMP//PSMG1//TPM1//E

52L1//CAMKK2//FBXW11//VEGFA//UBE2C//MAD2L1//ANAPC11//PSMA3//PSMA6//PSMA7//PSMB5//PSMC5//PSM

K//PRKACB//PBK//EIF2AK2//TEX14//STK3//AURKA//AURKC//BUB1//CDK11A//STK24//CCNE1//AKT1S1//PPM1K//P
B2IP//SOD2//IL1B//TPM1//HBB//GRB2//EEF1E1//SDCBP//TPD52L1//STC1//VEGFA//STC2//CCNB1//FEM1B//HP

6R3//CAMKK2//HSP90AA1//WASL//GCH1//HP//UBE2C//MAD2L1//ANAPC11//PSMA3//PSMA6//PSMA7//PSMB5//P

SMC5//PSMD9//PSMD11//PSMD12//PSME1//PSMA8//SKP1//FEM1B//UBE2S//STK3

PSMG1//RRM2//USP16//ALDOC//SOD2//PDSS2//HBB//CENPF//PSMD9
NG//CTNNB1//DAB2IP//GAL//ING3//STK3//TOP2A//EEF1E1//MAD2L1//MDM2//SOD2//VEGFA//MTDH//ADNP//GCL

E1//EIF5A2//PQBP1//CAMKK2//INHBA//SMAD5//MAX//MYBL1//MYC//NPM1//PSMD9//ZFX//MED30//FOXD4L1//FO
P1//NME1//TXN//TPD52L1//PPP6R3//CAMKK2//HSP90AA1//WASL//GCH1//FOXD4L1//FOXD4//FOXD2//FOXD4L3/

MD9//PSMD11//PSMD12//PSME1//SKP1//CCNB1//UBE2S//IFNG//STK3
D2//FBXW11//UBR5//SMURF1//UBE2D3//ISG15//HERC6//SMURF2//SRSF10//SMN2//SNRPD1//SNRPD2//LSM3//B

//LAG3//PSMA8//NME1//MST4//PSMA3//PSMA6//PSMA7//PSMB5//PSMC5//PSMD9//PSMD11//PSMD12//PSME1//

FNG//CTNNB1//DAB2IP//GAL//ING3//STK3//TOP2A//EEF1E1//MAD2L1//MDM2//SOD2//VEGFA//MTDH//ADNP//GC

NRK//PRKACB//PBK//EIF2AK2//TEX14//STK3//AURKA//AURKC//BUB1//CDK11A//STK24//CCNE1//AKT1S1//PPM1

52L1//CAMKK2//FBXW11//VEGFA//UBE2C//ANAPC11//PSMA3//PSMA6//PSMA7//PSMB5//PSMC5//PSMD9//PSMD

1//NLRP12//NGFRAP1//IFI27//CRH//CYCS//DAB2IP//MTDH//GH1//CTNNB1//GABPB1//HBB//HP//ADNP//ARPP19//

1//UBR5//SMURF1//UBE2D3//ISG15//HERC6//SMURF2//SRSF10//SMN2//SNRPD1//SNRPD2//LSM3//BCAS2//HN

3A//RAB7L1//CHMP4C//RAB33A//GOSR1//BIRC5//TXN//UBR5//MDM2//RRAGB//RRAGC//EGF//DAB2IP//IL1B//CY
RP2//RHOB//SDAD1//EIF5A2//RAB22A//RAB3A//RAB5A//KIF18A//RAB7L1//CHMP4C//RAB33A//GOSR1//ABCD3//

/PQBP1//CAMKK2//INHBA//SMAD5//MAX//MYBL1//MYC//NPM1//PSMD9//ZFX//MED30//FOXD4L1//FOXD4//FOXD

2S//IFNG//STK3
2//MTDH//C9ORF89//EEF1E1//MDM2//WNT9A//SDCBP//TPD52L1//PTPN1//SIRT4//RAB5A//EZH2//ADNP//CRH//

W11//STAG2//MAD2L1//INHBA//SOCS1//LAG3//SOD1//IL1B//HNRNPAB//SRSF10//NPPC//UBE2C//ANAPC11//PSM
RP12//FBXW11//STAG2//MAD2L1//SOCS1//INHBA//LAG3//HNRNPAB//SRSF10//NPPC//UBE2C//ANAPC11//PSMA

RP2//RHOB//SDAD1//EIF5A2//RAB22A//RAB3A//RAB5A//KIF18A//RAB7L1//CHMP4C//RAB33A//GOSR1
ANAPC11//PCNP//SKP1//UBE2D2//KLHL21//CYLD//EIF1AX//RPL7L1//GTF2H3//ASMT//MRPS7//MRPS23//BRIX1//

HMP4C//RAB33A//GOSR1//TXN//UBR5//EGF//DAB2IP//IL1B//CYLD//FBXW11//HSP90AA1//SEC61G//DPH3//PAN

SEC61G//RHOT1//RAB31//TGFBR1

//INHBA//SOCS1//LAG3//HNRNPAB//SRSF10//NPPC//UBE2C//MAD2L1//ANAPC11//PSMA3//PSMA6//PSMA7//PS[illegible]

[illegible]C//NPM1//PSMD9//ZFX//MED30//MTDH//FOXD4L1//FOXD4//FOXD2//ZFPM2//FOXD4L3//TFAP2E//AKIRIN2//NC[illegible]

TC1//HP//MYC//SLC30A8//CTNNB1//CD55//IL1B//BDKRB1//TAC1//HSPA8//RAB3A//CDO1//NME1//SYT9//SLC2A1/[illegible]

[illegible]3//BCAS2//HNRNPM//SYNCRIP//HNRNPA1//HNRNPH3//HNRNPK//U2AF1//SRSF9//GTF2H3//RAD51AP1//H2AF[illegible]

//MAB21L1//AKIRIN2//EIF5A2//TGFBR1//NLRP12//NGFRAP1//IFI27//CRH//CYCS//NPPC//NMU//DAB2IP//MTDH//[illegible]

PM1K//FBXW11//PPM1A//PPP3CA//PTPN1//PPM1D//CDC14A//IMPA1//ACYP1//DAB2IP//GH1//TOP1//BAZ1B//EYA
PM1K//FBXW11//PPM1A//PPP3CA//PTPN1//PPM1D//CDC14A//DAB2IP//GH1//TOP1//BAZ1B//EYA2//ABI1//NLRP1

23//SMURF1//STRADA//SDCBP//RTP4//TOMM5//ABCC5//SLC36A4//ENSA//HBB//NDUFA8//NDUFB2//NDUFB4//N

MM5//ABCC5//SLC36A4//ENSA//HBB//NDUFA8//NDUFB2//NDUFB4//NDUFB9//NDUFS6//TMED7//DYNC1LI1//ATP

.//STRADA//CENPF//CTNNB1//IFNG//MYC//NCF2//NME1//GAL//ABCD3//TOP1//YWHAZ//SOCS1//CCNE1//AQP10/

2A//RAB3A//RAB5A//RRAGC//RAB7L1//ARHGAP11B//RAB33A//ARHGAP25//GRB2//IGF1//SDCBP//NLRP12//NDC

YNCRIP//HNRNPA1//HNRNPH3//HNRNPK//U2AF1//SRSF9//GTF2H3//RAD51AP1//H2AFX//RAD51//EIF5A2//TPI1//

1//HNRNPH3//HNRNPK//U2AF1//SRSF9//GTF2H3//RAD51AP1//H2AFX//RAD51//PTTG2//RNASEH1//HMGB2//CCI

URF1//UBE2D3//ISG15//HERC6//SMURF2//SRSF10//SMN2//SNRPD1//SNRPD2//LSM3//BCAS2//HNRNPM//SYNC

A//HMGXB4//WWOX//SMURF1//FOXD4L1//FOXD4//FOXD2//FOXD4L3//TWSG1//TOB1//CD9//VEGFA//ADNP//CCI

;AL//PANX1//PCNA//SDS//STC2//CA2//NPPC//HMGB2//NME1//TGFBR1//CMPK2//RRAGB//RRAGC//DAB2IP//INHE

MAP3K3//EGF//TGFBR1//IL1B//SOD1//SPRED2//POLE2//RRAGC//CDK11A//MORF4L2//CAMLG//B2M//CCDC88A
30//MTDH//FOXD4L1//FOXD4//FOXD2//ZFPM2//FOXD4L3//TFAP2E//AKIRIN2//PRTFDC1//NCBP2//GTF2H3//RAD

P16//UBE2C//OIP5//CDCA2//DYNC1LI1//ANAPC11//TXNL4B//PBK//PAPD5//AURKA//DYNLT3//CDK11A//FAM83D//
KNTC1//DLGAP5//TRIP13//TOP2A//TRIAP1//SESN3//IFNG//MYC//PPM1A//RPRM//STRADA//CKS2//KIF11//AURKA
//BUB1//KIF18A//KNTC1//DLGAP5//TRIP13//AURKA//PTTG1//TOP2A//BEX2//TRIAP1//SESN3//IFNG//MYC//PPM1
UBE2C//CKS2//KIF11//AURKA//STMN1//USP16//OIP5//CDCA2//DYNC1LI1//ANAPC11//TXNL4B//PBK//PAPD5//DY

/DLGAP5//BTG3//DYNC1LI1//CTNNB1//TOP2A//CASC5//FEM1B//BEX2//CCNE1

5//YWHAZ//RAB3A//CTNNB1//STMN1//BNIP1//NPLOC4//ABCD3//RAB22A//UBE2C//CKS2//KIF11//AURKA//RAD51

AC//H2AFV//SDCBP//CAP2//SGCD//TPM1//MAN2A1//RNASEH1//CYCS//SOD1//FOXL2//TPD52L1//EZH2//BPTF//S
NAP1L4//NPM1//H2AFJ//HIST2H2AC//H2AFV//SDCBP//CAP2//SGCD//TPM1//MAN2A1//RNASEH1//CYCS//SOD1//

HMGB2//NAP1L4//NPM1//H2AFJ//HIST2H2AC//H2AFV//SDCBP//SGCD//TPM1//MAN2A1//RNASEH1//CYCS//SOD

IP5//H2BFM//H2AFX//H2AFZ//HMGB2//NAP1L4//NPM1//H2AFJ//HIST2H2AC//H2AFV//SDCBP//SGCD//TPM1//MA

/NUDT7//NUDT3//PRKACB//CDO1//NPLOC4//UBE2S//MAD2L1//ANAPC11//PSMD11//PSMD12//PSME1//AURKA//

FI27//NLRP12//TOP1//PRTFDC1//CRH//HBB//HP//NME1//BEX2//NAE1//TOP2A//IFNG//DAB2IP//GAL//ING3//STK3/
PM1A//RPRM//STRADA//ZWINT//BUB1//KNTC1//CENPF//ABI1//TOB1//BTG3//NPPC//NOX4//GAL//EIF2AK2//RAR
12//ZFYVE27//HSPB8//SMN2//TOP1//PRTFDC1//CRH//HBB//HP//NME1//BEX2//NAE1//TOP2A//IFNG//DAB2IP//GA

T//INHBA//IFI27//NLRP12//CRH//NME1//BEX2//NAE1//TOP2A//IFNG//DAB2IP//GAL//ING3//STK3//EEF1E1//MAD2L
23//BRIX1//MRPL47//RPL27A//MRPL12//RPS7//RPS14//RPS15//RPS24//MRPS25//EIF4H//SECISBP2//RPL23//MR

R1//SYCP2//TRIM69//EYA2//ALDOC//KIF1B//JMJD6//MAGI3//CKAP2//MAGEH1//UBQLN1//FAM3B//RHOT1//RRA

INHBA//SKP1//CCNE1//CEP152//HSP90AA1//HAUS2//TPD52L1//PPM1D//KIF20A//CENPA//STAG2//CENPI//BUB1

PM1A//RPRM//STRADA//ZWINT//BUB1//KNTC1//CENPF//ABI1//TOB1//BTG3//NPPC//NOX4//GAL//EIF2AK2//RAR

NGLY1//MCCC2//SDS//HSD17B6//MTDH//NUDT7//NUDT3//PLA2G16//PLD6//PLA2G4C//PRKACB//CDO1//NPLOC
SC5//INHBA//NMU//SLC30A8//GAL//FAM3B//PSMD9//TXN//UBR5//MDM2//RRAGB//RRAGC//DAB2IP//NUSAP1//C

OB//NLRP12//INHBA//NMU//SLC30A8//GAL//FAM3B//PSMD9//TXN//UBR5//DAB2IP//NUSAP1//CYLD//FBXW11//AT

P90AA1//SDAD1//BBIP1//STOM//POMP//PSMG1//TPM1//BAIAP2//HAUS2//RRM2//USP16//ALDOC//SOD2//PDSS

CIP//RFC5//TOP2A//UBE2D3//PTTG1//MORF4L2//HMGB2//BAZ1B//TRIP13//CCL26//CDO1//CRH//NFX1//NOX4//G

//SOD2//PDSS2//HBB//CENPF//PSMD9

NP//PSMD9//MTDH//HSPA1B//KIF11

AIAP2//HAUS2//RRM2//USP16//ALDOC//SOD2//PDSS2//HBB//CENPF//ADNP//PSMD9//MTDH//HSPA1B//KIF11

D9//PSMD11//PSMD12//PSME1//PSMA8//SKP1//FEM1B//UBE2S//STK3

PPM1A//PPP3CA//PTPN1//PPM1D//CDC14A//SIRT4//TPST1//ST6GALNAC2//DPM1//MAN2A1//POMT1//UBE2W//D

PSMC5//PSMD9//PSMD11//PSMD12//PSME1//PSMA8//SKP1//CCNB1//FEM1B//UBE2S//IFNG//STK3

M//NCF2//AKT1S1//TGFBR1//LAG3

XD4//FOXD2//ZFPM2//FOXD4L3//TFAP2E//AKIRIN2//PRTFDC1//NCBP2//GTF2H3//VEGFA//RAD51//UBE2C//PSM
//RHEBL1//PSMA6//MTDH//DPH3//HP//UBE2C//MAD2L1//ANAPC11//PSMA3//PSMA7//PSMB5//PSMC5//PSMD9//P

CAS2//HNRNPM//SYNCRIP//HNRNPA1//HNRNPH3//HNRNPK//U2AF1//SRSF9//GTF2H3//RAD51AP1//H2AFX//RA

BEX2//NAE1

LM//NCF2//AKT1S1//TGFBR1//LAG3

K//PPM1A//PPP3CA//PTPN1//PPM1D//CDC14A//SIRT4//TPST1//ST6GALNAC2//DPM1//MAN2A1//POMT1//UBE2W

11//PSMD12//PSME1//PSMA8//SKP1//FEM1B//UBE2S//STK3

SBDS//EIF4A3//NMU//PPM1A//FOXD4L1//FOXD4//FOXD2//FOXD4L3//TWSG1//NPPC//GRB2//SMURF1//CCNB1//

RNPM//SYNCRIP//HNRNPA1//HNRNPH3//HNRNPK//U2AF1//SRSF9//GTF2H3//RAD51AP1//H2AFX//RAD51//CAM

LD//FBXW11//HSP90AA1//SEC61G//DPH3//PANX1//CCNB1//TGFBR1//CENPA//SUN1
IL1B//BIRC5//TXN//UBR5//MDM2//RRAGB//RRAGC//EGF//DAB2IP//CYLD//FBXW11//INHBA//HSP90AA1//SEC61G

)2//ZFPM2//FOXD4L3//TFAP2E//AKIRIN2//PRTFDC1//NCBP2//GTF2H3//VEGFA//RAD51//UBE2C//PSMA8//ANAPC

CTHRC1//CYLD//STK3//UBR5

MA3//PSMA6//PSMA7//PSMB5//PSMD9//PSMD11//PSMD12//PSME1//HP
3//PSMA6//PSMA7//PSMB5//PSMD9//PSMD11//PSMD12//PSME1

MRPL47//RPL27A//MRPL12//RPS7//RPS14//RPS15//RPS24//MRPS25//EIF4H//SECISBP2//RPL23//MRPL33//PUM

K1//TGFBR1//MDM2

MB5//PSMD9//PSMD11//PSMD12//PSME1//HP

3P2//GTF2H3//ADNP//VEGFA//RAD51//UBE2C//PSMA8//ANAPC11//PSMA3//PSMA6//PSMA7//PSMB5//PSMD11//P

//UTS2//GCH1//NPPC//CFDP1//CDC42SE2//NDC80//SGOL1//CASC5//EIF5A2//INHBA//NMU//EIF2AK2//RHOT1//G

X//RAD51//TPI1//NME1//PRTFDC1//NPPC//RAB31//GCH1//NKIRAS1//TRIM23//RHOB//RAB22A//RAB3A//RAB5A//

GH1//GABPB1//HBB//HP//ADNP//ARPP19//SBDS//EIF4A3//PPM1A//FOXD4L1//FOXD4//FOXD2//FOXD4L3//TWSG

\2//ABI1//NLRP12//ADNP//STRADA//IFNG//DUSP14//INHBA//SDCBP//IGF1//TPD52L1//PPP6R3//MAP3K3//NEK2//
2//ADNP//STRADA//IFNG//DUSP14//INHBA//SDCBP//IGF1//TPD52L1//PPP6R3//MAP3K3//NEK2//IMPA1//VEGFA//

IDUFB9//NDUFS6//TMED7//DYNC1LI1//ATP5G1//CYCS//ENY2//DYNLT3//TXN//UQCRB//EIF4A3//ATP5S//KCNF1//

'5G1//CYCS//ENY2//DYNLT3//TXN//UQCRB//EIF4A3//ATP5S//KCNF1//KCNK1//ZDHHC13//TMEM38B//SLC4A8//PA

/CD9//ADNP//SLC30A8//CA2//IMPA1//CDO1//LUM//IFI6//HLA-B//IFI27//IFI35//OAS1//OAS2//PTPN1//ISG15//PCNA

80//UBE2C//ZWINT//CKS2//HMGB2//PCNA//AURKA//TOP2A//AKT1S1//CCDC88A//RRAGB//IFNG//CTNNB1//ZDH

/SOD1//ERH//OAS1//OAS2//NME1//PRTFDC1//NPPC//RAB31//GCH1//NKIRAS1//TRIM23//RHOB//RAB22A//RAB3

NE2//ESCO2//CCDC88A//STRA13//RDM1//EYA2//UBE2T//NPM1//BCCIP//RAD51C//UBE2D3//PTTG1//MORF4L2//

RIP//HNRNPA1//HNRNPH3//HNRNPK//U2AF1//SRSF9//GTF2H3//RAD51AP1//H2AFX//RAD51//CAMLG//CCDC8

NE1//AKT1S1//RRAGB//RRAGC//PSMD9//EPN1//SOCS1//TBK1//MAP3K3//ZDHHC13//PELI2//MTDH//C9ORF89//

3A//PRKACB//SSTR2//CCNB1

//SOCS1//BDKRB1//PELI2//CCL26//HMGB2//VEGFA//TNNI3//IFNG//MYC//TBK1//YWHAZ//NME1//TPM1//SOD2//BI
51

/PTTG1//KLHL21//MDM2//CYLD//RPRM//UBE2S//NAE1//WNT9A//SBDS//EGF//IGF1//IL1B//BTG3//TOP2A
A//STMN1//RAD51C//USP16//OIP5//CDCA2//DYNC1LI1//TXNL4B//PBK//PAPD5//DYNLT3//CDK11A//FAM83D//PTT
A//RPRM//STRADA//KIF11//STMN1//RAD51C//USP16//OIP5//CDCA2//DYNC1LI1//TXNL4B//PBK//PAPD5//DYNLT3
NLT3//CDK11A//FAM83D//PTTG1//KLHL21//MDM2//H2AFX//RAD51//MND1//SYCP2//UBR2//UBE2S//WNT9A//SBD

C//USP16//CDCA2//DYNC1LI1//ANAPC11//TXNL4B//HAUS2//PBK//PAPD5//DYNLT3//CDK11A//FAM83D//CCNB2//

SRSF9//MRPL12//RPL27A//RPS7//RPS24//RPL23//TOMM5//YWHAZ//CTNNB1//MST4//STK24//RAB3A//STMN1//BN
/FOXL2//TPD52L1//EZH2//BPTF//SBDS//RPS7//RPS24//UTP23//EIF4A3//SRSF9//MRPL12//RPL27A//RPL23//TOM

)1//FOXL2//TPD52L1//EZH2//BPTF//SRSF9//MRPL12//RPL27A//RPS7//RPS24//RPL23//GCH1//MDM2//PARD6B//W

N2A1//RNASEH1//CYCS//SOD1//FOXL2//TPD52L1//EZH2//BPTF//SBDS//RPS7//RPS24//UTP23//EIF4A3//SRSF9//

CCNB1//PTTG1//FBXW11//ACAP2//TBC1D2B//TNNI3//TPM1//CCL26//DAB2IP//HBB//PCNP//GAPDHS//IGF1//PLA

/EEF1E1//MAD2L1//MDM2//VEGFA//MTDH//ADNP//GCLM//NCF2//AKT1S1//TGFBR1//ZFYVE27//HSPB8//SMN2
RES3//SOD2//SSTR2//WNT9A//PPM1D//CD9//EEF1E1//NLRP12//DAB2IP//FOXD4L1//FOXD4//FOXD2//FOXD4L3
AL//ING3//STK3//EEF1E1//MAD2L1//MDM2//VEGFA//MTDH//ADNP//GCLM//NCF2//AKT1S1//TGFBR1

.1//MDM2//VEGFA//MTDH//ADNP//GCLM//NCF2//AKT1S1//TGFBR1//TOP1
PL33//PUM2//NGDN//TACO1//EEF1E1//NCBP2//GGCX//UBE2S//CAMKK2//TP53RK//TBK1//MST4//SNRK//PRKAC

GC//BUB1//CDK11A//UBE2D3//PDCL3//SLTM

//KIF18A//KNTC1//DLGAP5//CDO1//CTNNB1//BPTF//ZFPM2//SUZ12//FOSB//HSBP1//NFX1//TXN//MTDH//TCEAL

RES3//SOD2//SSTR2//WNT9A//PPM1D//CD9//EEF1E1//NLRP12//DAB2IP//FOXD4L1//FOXD4//FOXD2//FOXD4L3

C4//UBE2S//MAD2L1//ANAPC11//PSMD11//PSMD12//PSME1//AURKA//CCNB1//PTTG1//FBXW11//ACAP2//TBC1D
CYLD//FBXW11//ATP5G1//SEC61G//SIRT4//RHOT1//RAB31//DPH3//PANX1//BIRC5//CENPF//CRH//ZWINT//TGFBI

TP5G1//SEC61G//SIRT4//RHOT1//RAB31//DPH3//PANX1//BIRC5//CENPF//CRH//TGFBR1//ZWINT

2//HBB//CENPF//ADNP//PSMD9//MTDH//HSPA1B//KIF11

GAL//BDKRB1//CCL7//TNFAIP6//TPST1//PLA2G4C//NCF2//UBR5//PSMA8//MDM2//TRIAP1//PSMA3//PSMA6//PSM

)AB2IP//GH1//ADNP//EZH2//SUZ12//BAZ1B//EYA2//USP16//ENY2//DPH3//GGCX//ABI1//FKBP14//MAGT1//B4GAL

IA8//ANAPC11//PSMA3//PSMA6//PSMA7//PSMB5//PSMD11//PSMD12//PSME1//SKP1//UBE2S
PSMD11//PSMD12//PSME1//PSMA8//SKP1//CCNB1//FEM1B//UBE2S//IFNG//STK3

AD51//CAMLG//CCDC88A//SOCS1//BDKRB1//CCNB1//PELI2//CCT7//DNAJC21//HSPA8//NKTR//FKBP14//DNAJA4

N//DAB2IP//GH1//ADNP//EZH2//SUZ12//BAZ1B//EYA2//USP16//ENY2//DPH3//ABI1//FKBP14//MAGT1//B4GALT4//

/UBE2C//BIRC5//RRAGB//RRAGC//SLC30A8//CA2//UBR5//SDCBP//GAL//ING3//SOD1//STK3//TOP2A//EEF1E1//T

LG//CCDC88A//SOCS1//BDKRB1//CCNB1//PELI2//CCT7//DNAJC21//HSPA8//NKTR//FKBP14//DNAJA4//HSPA1B/

G//DPH3//PANX1//ENY2//EIF4A3//CCNB1//TGFBR1//CENPA//SUN1

C11//PSMA3//PSMA6//PSMA7//PSMB5//PSMD11//PSMD12//PSME1//SKP1//UBE2S

2//NGDN//TACO1//EEF1E1//NCBP2//GGCX//UBE2S//CAMKK2//TP53RK//TBK1//MST4//SNRK//PRKACB//PBK//EI

'SMD12//PSME1//SKP1//UBE2S

AL//FAM3B//PLSCR1//ASMT//RPS14//SFXN1//TGFBR1//CCDC85B//HSPA1B//CCL26//AURKA//FBXW11//MDM2//[illegible]

RRAGC//RAB7L1//RAB33A//KIF1B//PSMC5//ERH//CMPK2//PTTG2//RNASEH1//HMGB2//CCNE2//ESCO2//CCDC[illegible]

1//GRB2//SMURF1//CCNB1//UBE2C//BIRC5//RRAGB//RRAGC//SLC30A8//CA2//UBR5//SDCBP//FBXW11//GAL//IN[illegible]

VEGFA
'ACYP1

'KCNK1//ZDHHC13//TMEM38B//SLC4A8//PANX1//ATP13A3//KCTD20//SLN//COX17//SFXN1//SLC30A7//SLC30A8

ANX1//ATP13A3//KCTD20//SLN//COX17//SFXN1//SLC30A7//SLC30A8//PRKACB//AQP10//HSP90AA1//NPM1//PCN

//ITGAM//SBDS//MTDH//GH1//AURKA//DCN//GCH1//AKIRIN2//BDKRB1//CLDN4//GGCX//PANX1//SDS//TPM1//HB

IHC13//PPM1A//PELI2//BST2//MTDH//C9ORF89//EEF1E1//TPD52L1//MAGI3//WSB1//STMN1//PLA2G4C//SH3BP5

A//RAB5A//RRAGC//RAB7L1//RAB33A//KIF1B//PSMC5//CMPK2//PTTG2//RNASEH1//HMGB2//CCNE2//ESCO2//C

SOD1//BAZ1B//TRIP13//CYCS//FOXL2//TPD52L1//MND1//SIRT4//TRAPPC2//RRAGC//FOXD4//FOXD2//GABPB1//

3A//SOCS1//BDKRB1//CCNB1//PELI2//CCT7//DNAJC21//HSPA8//NKTR//FKBP14//DNAJA4//HSPA1B//HSP90AA1//
EEF1E1//TPD52L1//PTPN1//SIRT4//RAB5A//EZH2//CTHRC1//CYLD//STK3//UBR5//OIP5

EX2//GGCX//GRB2//HBB//IGF1//ITGA2B//ITGAM//KIF11//RHOB//LAMP2//PPBP//PRKACB//PTPN1//RAB5A//RAD5

G1//KLHL21//NPM1//RAD51//MND1//SYCP2//UBR2//UBE2S//CTNNB1//NAE1//WNT9A//SBDS//EGF//IGF1//IL1B
3//CDK11A//FAM83D//KLHL21//NPM1//RAD51//MND1//SYCP2//UBR2//CYLD//UBE2S//CTNNB1//NAE1//WNT9A//SI
)S//EGF//IGF1//IL1B

/KLHL21//DLGAP5//SYCP2//ABI1//WASL//EYA2//KDM2A//JMJD6//SUZ12//ING3//ENY2//HIRA//MORF4L2//CCL26//I

NIP1//NPLOC4//ABCD3//RAB22A//PARD6B//UBE2C//CKS2//KIF11//AURKA//RAD51C//USP16//CDCA2//DYNC1LI1
M5//YWHAZ//CTNNB1//MST4//STK24//RAB3A//STMN1//BNIP1//NPLOC4//ABCD3//RAB22A//PARD6B//UBE2C//CI

/ASL//REPS2//TOMM5//YWHAZ//RABGEF1//EPN1//NME1//RAB22A//RAB5A//CDC42SE2//MST4//STK24//NPLOC4

/MRPL12//RPL27A//RPL23//GCH1//MDM2//PARD6B//WASL//REPS2//TOMM5//YWHAZ//RABGEF1//EPN1//NME1//

2G4C//IFNG//IL1B//PAPD5//HP

//FOXL2//CCDC85B//EZH2//HMGB2//AKIRIN2//SMURF2//HIRA//EIF4A3//HMGXB4//WWOX//TPM1//SMURF1//TWS

B//PBK//EIF2AK2//TEX14//STK3//AURKA//AURKC//BUB1//CDK11A//STK24//CCNE1//AKT1S1//PPM1K//PPM1A//P

1//CAMKK2//MAP3K3//DCP2//NCBP2//UPF3B//EIF4A3//TGFBR1//IL1B//SOD1//SPRED2//FBXW11//UBR5//SMURF

//FOXL2//ADNP//TBK1//CCDC85B//EZH2//HMGB2//AKIRIN2//SMURF2//HIRA//EIF4A3//HMGXB4//WWOX//SMURF

2B//TNNI3//TPM1//CCL26//DAB2IP//HBB//DHDH//PCNP//IGF1//IL1B//IFNG//PAPD5//HP
R1//SUN1

A7//PSMB5//PSMC5//PSMD9//PSMD11//PSMD12//PSME1//GCLM//NDUFB4//CRKL//DAB2IP//HSPB8//DNAJA4//H

T4//JMJD6//DCN//NAE1//UBQLN1//NLRP12//ISG15//STRADA//IFNG//ESCO2//DUSP14//UBE2S//UBE2T//IGF1//TF

4//HSPA1B//HSP90AA1//EIF5A2//FEM1B//DCAF7//MARCH6//UBE2C//DCAF4//TRIM23//ANAPC11//PCNP//SKP1//U

JMJD6//DCN//NAE1//UBQLN1//NLRP12//ISG15//STRADA//IFNG//ESCO2//DUSP14//UBE2T//IGF1//TPD52L1//UBE

BK1//MAP3K3//ZDHHC13//BST2//BPTF//FBXW11//HNRNPAB//NCF2//HSP90AA1//CCNE1//JUND//H2AFX//TPM1//

/HSP90AA1//EIF5A2//FEM1B//DCAF7//MARCH6//UBE2C//DCAF4//TRIM23//ANAPC11//PCNP//SKP1//UBE2D2//KL

F2AK2//TEX14//STK3//AURKA//AURKC//BUB1//CDK11A//STK24//CCNE1//AKT1S1//PPM1K//PPM1A//PPP3CA//PT

PSMD9//SLC30A7//CA2//ADNP//RPS24//CRABP2//CCNB2//HMGB2//NCF2//AKT1S1//SIRT4//JMJD6//BPGM//FOXI

38A//STRA13//RDM1//EYA2//UBE2T//NPM1//BCCIP//RAD51C//UBE2D3//PTTG1//MORF4L2//SOD1//BAZ1B//TRIP1

NG3//STK3//TOP2A//EEF1E1//MAP3K3//ZDHHC13//BST2//BPTF//HNRNPAB//NCF2//HSP90AA1//CCNE1//JUND//H

//PRKACB//AQP10//HSP90AA1//PCNA//RIMS2//HSPA8//RABGEF1//EPN1//NME1//RAB22A//RAB5A//CDC42SE2//

NA//RIMS2//HSPA8//RABGEF1//EPN1//NME1//RAB22A//RAB5A//CDC42SE2//DLGAP5//KIF18A//CCNB1//TOB1//R

B//HP//HMGB2//FOXD4L1//FOXD4//FOXD2//FOXD4L3//S100A16//TGFBR1//CMPK2//RRAGB//RRAGC//WNT9A//[illegible]

CDC88A//STRA13//RDM1//EYA2//UBE2T//NPM1//BCCIP//RAD51C//UBE2D3//PTTG1//MORF4L2//BAZ1B//TRIP13[illegible]

/HNRNPAB//MYC//HIRA//PQBP1//HOXB13//NFAT5//ZNF813//CRABP2//ZNF534//ZNF483//ZFP1//EZH2//ADNP//FC[illegible]

/EIF5A2//FEM1B//DCAF7//MARCH6//UBE2C//DCAF4//TRIM23//ANAPC11//PCNP//SKP1//UBE2D2//KLHL21//PTTG[illegible]

1C//SLC16A1//TFPI//TFPI2//CALU//KIF18A//CD9//KIF23//CYLD//FBXW11//PPM1A//WNT9A//RRAGB//LRRN2//BAI/

BDS//PLA2G16//RHOB//EGF//IGF1//IL1B//BTG3//FEM1B//ESCO2//SENP5//ERH//FBXW11//CKAP2//MKI67//PCNP

MYC//NDUFAF4//LETM1//SBDS//POLE4//HSP90AA1//EGF//IGF1//IL1B//SOD1//CYLD//SUN1

//ANAPC11//TXNL4B//HAUS2//PBK//PAPD5//DYNLT3//CDK11A//FAM83D//CCNB2//KLHL21//DLGAP5//SYCP2//BA
KS2//KIF11//AURKA//RAD51C//USP16//CDCA2//DYNC1LI1//ANAPC11//TXNL4B//HAUS2//PBK//PAPD5//DYNLT3//

4//RAB3A//STMN1//BNIP1//ABCD3//UBE2C//CKS2//KIF11//AURKA//RAD51C//USP16//CDCA2//DYNC1LI1//ANAPC

/RAB22A//RAB5A//CDC42SE2//MST4//STK24//NPLOC4//RAB3A//STMN1//BNIP1//ABCD3//UBE2C//CKS2//KIF11//

SG1//STMN1//NAE1//AKT1S1//FBXW11//STAG2//EGF//GRB2//EPN1//CYLD//SOCS1//VEGFA//C9ORF89//SBDS//A

PP3CA//PTPN1//PPM1D//CDC14A//SIRT4//TPST1//ST6GALNAC2//DPM1//MAN2A1//POMT1//PSMA8//PSMA3//PS

F1//UBE2D3//ISG15//HERC6//SMURF2//CCDC88A//TRIP13//SRSF10//SMN2//SNRPD1//SNRPD2//AURKA//LSM3//

F1//TPM1//TWSG1//NCBP2//STMN1//NAE1//AKT1S1//CYLD//UBE2D3//ATG12//ISG15//FBXW11//STAG2//EGF//GR

OXB13//PLSCR1//EIF2AK2//GCH1//TPM1//DCN//CCNB1//SSTR2//EEF1E1//CD55//AKIRIN2//BST2//LAG3//SDCBI

D52L1//UBE2G2//POLE4//ING3//MAP3K3//NEK2//VEGFA//MAD2L1//PSMA3//PSMA6//PSMA7//PSMB5//PSMC5//

BE2D2//KLHL21//ALDOC//GAPDHS//PRKACB//TPI1//MAN2A1//SLC16A1//COX17//COX6A1//COX6C//SDS//BPG

2G2//POLE4//ING3//MAP3K3//NEK2//VEGFA//MAD2L1//PSMA3//PSMA6//PSMA7//PSMB5//PSMC5//PSMD9//PSM

/GAPDHS//NUSAP1//DLGAP5//WNT9A//USP16//ENY2//MRPL12//PQBP1//CAMKK2//SMAD5//MAX//MYBL1//NPM

LHL21//PTTG2//RNASEH1//HMGB2//ESCO2//STRA13//RDM1//EYA2//UBE2T//NPM1//RAD51C//PTTG1//MORF4L2

[illegible]PN1//PPM1D//CDC14A//SIRT4//TPST1//ST6GALNAC2//DPM1//MAN2A1//POMT1//CPA3//SENP5//ANPEP//HP//F[illegible]

D4L1//FOXD4//FOXD2//FOXD4L3//PDSS2//GCLM//IFI6//CCNB1//SUN1

13//CYCS//FOXL2//TPD52L1//MND1//SIRT4//TRAPPC2//FOXD4//FOXD2//GABPB1//HNRNPAB//MYC//HIRA//PQBI

H2AFX//RHOB//GAPDHS//NUSAP1//DLGAP5//WNT9A//USP16//ENY2//MRPL12//PQBP1//CAMKK2//SMAD5//MAX/

CTNNB1//DLGAP5//KIF18A//CCNB1//TOB1//RAB3A//NFAT5//CDO1//SYT9//IL1B//SLC2A1//KIF1B//HMGXB4//RHO

AB3A//NFAT5//CDO1//SYT9//IL1B//SLC2A1//KIF1B//HMGXB4//RHOB//NLRP12//CENPF//RAB31//SNF8//CHMP4B/

)AB2IP//INHBA//PRKACB//MPP1

//CYCS//FOXL2//TPD52L1//MND1//SIRT4//TRAPPC2//FOXD4//FOXD2//GABPB1//HNRNPAB//MYC//HIRA//PQBP1

)XD4L3//TFAP2E//ZNF860//SMAD5//MYBL1//ZNF331//ZNF287//CDK11A//ZNF382//CHD1//SNF8//ASCL4//JUND//S

32//RNASEH1//HMGB2//ESCO2//STRA13//RDM1//EYA2//UBE2T//NPM1//RAD51C//PTTG1//MORF4L2//BAZ1B//TR

AP2//CAP2//NFAT5//NMU//FGL2//TAGAP//RHEBL1//CRABP2//CRH//GRP//HNRNPK//HSP90AA1//SP110//IMPA1//S

//AURKC//PARD6B//CDC14A

AIAP2//GRB2//HSP90AA1//ITGA2B//KAL1//RHOB//WASL//ABI1//ADNP//UBE2S//EYA2//KDM2A//JMJD6//SUZ12//IN
CDK11A//FAM83D//CCNB2//KLHL21//DLGAP5//SYCP2//BAIAP2//GRB2//HSP90AA1//ITGA2B//KAL1//RHOB//WASI

C11//TXNL4B//HAUS2//PBK//PAPD5//DYNLT3//FAM83D//CCNB2//KLHL21//DLGAP5//SYCP2//CD9//BAIAP2//GRB2/

AURKA//RAD51C//USP16//CDCA2//DYNC1LI1//ANAPC11//TXNL4B//HAUS2//PBK//PAPD5//DYNLT3//FAM83D//CC

ADNP//GCLM//LAG3//PLA2G16//RHOB//TGFBR1//HNRNPAB//PTPN1//SRSF10//DPH3//TOP2A//CASC5//CTHRC1/

SMA6//PSMA7//PSMB5//USP16//UBR2//PSMD9//UBE2G2//USP13//UBE2W//DAB2IP//GH1//ADNP//SBDS//EIF4A3/

BCAS2//HNRNPM//SYNCRIP//HNRNPA1//HNRNPH3//HNRNPK//U2AF1//SRSF9//GTF2H3//RAD51AP1//RAD51//C

RB2//EPN1//CRH//SOCS1//VEGFA//C9ORF89//SBDS//GCLM//LAG3//PLA2G16//RHOB//TGFBR1//HNRNPAB//PTPN

P//TPD52L1//B2M//NLRP12//RSAD2//IFNG//OAS1//OAS2//SOCS1//IFI6//IFI27//IFI35//ISG15//STC1//STC2//FEM1B/

PSMD9//PSMD11//PSMD12//PSME1//PSMA8//CYLD

M//IDH3A//SLC2A1//NDUFA8//NDUFB2//NDUFB4//NDUFB9//NDUFS6//ATP5G1//CYCS//UQCRB//COX7A2L//GCH

ID11//PSMD12//PSME1//PSMA8//CYLD

I//ZFX//MED30//ZFPM2//TFAP2E//LAG3//PRTFDC1//NCBP2//GTF2H3//NME1//PANX1//RAD51//STMN1//ANAPC11

2//BAZ1B//TRIP13//CYCS//FOXL2//TPD52L1//MND1//SIRT4//TRAPPC2//RRAGC//FOXD4//FOXD2//GABPB1//HNRI

\M108B1//NRIP3//PSMA8//PSMA3//PSMA6//PSMA7//PSMB5//USP16//UBR2//PSMD9//UBE2G2//USP13//UBE2W//

P1//HOXB13//NFAT5//ZNF813//CRABP2//ZNF534//ZNF483//ZFP1//EZH2//ADNP//FOXD4L3//TFAP2E//ZNF860//SM

/MYBL1//NPM1//ZFX//MED30//ZFPM2//TFAP2E//LAG3//PRTFDC1//NCBP2//GTF2H3//NME1//PANX1//RAD51//STM

B//TGFBR1//NLRP12//SGOL1//CASC5//DAB2IP//CENPF//RAB31//SNF8//CHMP4B//CHCHD4//RAB40AL//RAB37//

/CHCHD4//VPS13B//RAB40AL//RAB37//SERP2//SDAD1//EIF5A2//RAB7L1//CHMP4C//RAB33A//SLC16A1//GH1//V

//HOXB13//NFAT5//ZNF813//CRABP2//ZNF534//ZNF483//ZFP1//EZH2//ADNP//FOXD4L3//TFAP2E//ZNF860//SMA

OD2//MED10//SBDS//RPS7//RPS15//RPS24//UTP23//CCRN4L//MAX//PSMC5//PIR//MED30//MRPL12//ZFC3H1//W

.IP13//CYCS//FOXL2//TPD52L1//MND1//SIRT4//TRAPPC2//RRAGC//FOXD4//FOXD2//GABPB1//HNRNPAB//MYC//

STMN1//SMAD5//MPP1//NPM1//ZDHHC13//CCL7//STK3//BST2//TNFAIP6//STK24//NAE1//ARHGAP11B//ANGPTL1

G3//ENY2//HIRA//MORF4L2//POSTN//LUM//TGFBR1//VMO1//CCL26//CD9//MYC//NDUFAF4//PVRL3//LIMS1//BBIF
_//ABI1//ADNP//UBE2S//EYA2//KDM2A//JMJD6//SUZ12//ING3//ENY2//HIRA//MORF4L2//POSTN//LUM//TGFBR1//V

//HSP90AA1//ITGA2B//KAL1//RHOB//ABI1//PLSCR1//DAB2IP//ADNP//UBE2S//HSPA8//EYA2//KDM2A//JMJD6//SU

:NB2//KLHL21//DLGAP5//SYCP2//CD9//BAIAP2//GRB2//HSP90AA1//ITGA2B//KAL1//RHOB//ABI1//PLSCR1//DAB2

'/STK3//DYNC1LI1//HP

/EZH2//SUZ12//BAZ1B//EYA2//ENY2//DPH3//ABI1//MAGT1//B4GALT4//JMJD6//DCN//NAE1//NPLOC4//MAD2L1//F

CAP2//NOX4//AURKC//KLHL21//RRAGC//CDK11A//MORF4L2//TNNI3//VEGFA//WWOX//FOXD4L1//FOXD4//FOXD

N1//SRSF10//DPH3//TOP2A//CASC5//CTHRC1//STK3//DYNC1LI1//HP

/NMU//CA2

1//ERH//OAS1//OAS2//NME1//PRTFDC1//NPPC//RAB31//NKIRAS1//RHOB//RAB22A//RAB3A//RAB5A//RRAGC//R

//SKP1//UBE2S//PPBP

NPAB//MYC//HIRA//PQBP1//HOXB13//NFAT5//ZNF813//CRABP2//ZNF534//ZNF483//ZFP1//EZH2//ADNP//FOXD4L

'CD55//DAB2IP//TFPI//GH1//ADNP//SBDS//EIF4A3//EZH2//SUZ12//BAZ1B//EYA2//ENY2//DPH3//ABI1//MAGT1//B4

IAD5//MYBL1//ZNF331//ZNF287//CDK11A//ZNF382//CHD1//SNF8//ASCL4//JUND//SOD2//MED10//SBDS//RPS7//R

MN1//ANAPC11//SKP1//UBE2S//PPBP//MAD2L1//DYNC1LI1

'SERP2//SDAD1//EIF5A2//RAB7L1//CHMP4C//RAB33A//SLC16A1//GH1//VDAC3//ABCD3//ARPP19//CTHRC

'DAC3//ABCD3//ARPP19//INHBA//GAL//FAM3B//GRB2//PSMD9//UBR5//DAB2IP//NUSAP1//CYLD//FBXW11//JMJD

D5//MYBL1//ZNF331//ZNF287//CDK11A//ZNF382//CHD1//SNF8//ASCL4//JUND//SOD2//MED10//SBDS//RPS7//RP[illegible]

/BP4//SNRNP48//JMJD6//SREK1IP1//TXNL4B//HSPA1B//EEF1E1//INHBA//DAB2IP//IL1B//FOXD4L1//IFNG//WNT9

/HIRA//PQBP1//HOXB13//NFAT5//ZNF813//CRABP2//ZNF534//ZNF483//ZFP1//EZH2//ADNP//FOXD4L3//TFAP2E//[illegible]

//NLRP12//SH3BP5//GDF15//ARHGAP25//ABI1//CRKL//MAGI3//WSB1//SDCBP//PLA2G4C//ESCO2//SIRT4//BAZ1

P1//LETM1//SBDS//POMP//PSMG1//POLE
/MO1//CCL26//CD9//MYC//NDUFAF4//PVRL3//LIMS1//SDAD1/

Z12//ING3/

IP//ADNP//UBE2S//HSPA8//EYA2

ᴾSMC5//PSMD11//PSMD12//

2//FOXD4L

AB7L1//RAB33A//KIF1B//

.3//TFAP2E//ZNF860//SMAD5//MYB

GALT4//JMJD

RPS15//RPS24//UTP23//CCRN4L//MAX//PIR//MED30//MRPL12//ZFC3H1//W

)6//SEC61G//

S15//RPS24//UTP23//CCRN4L//MAX//PI

A//PSMA8//HSPA8//PSMA3//

ZNF860//SMAD5//MYBL

B//PQ
