# Supplementary material for: LncRNAs as new biomarkers to differentiate triple negative breast cancer from non-triple negative breast cancer
Source: Oncotarget. 2016 Feb 19;7(11):13047–59. doi: 10.18632/oncotarget.7509 (PMC4914340; doi:10.18632/oncotarget.7509)
Supplement: Supplementary file 2 [file oncotarget-07-13047-s002.pdf]

#Column "GO.ID" stands for the ID of gene ontology term
#Column "Term" stands for the name of gene ontology term
#Column "Ontology" stands for the ontology of GOID belongs to
#Column "Count" stands for the number of DE genes associated with the listed GOID
#Column "Pop.Hits" stands for the number of background population genes associated with the
#Column "List.Total" stands for the total number of DE genes
#Column "Pop.Total" stands for the total number of background population genes
#Column "Fold.Enrichment" stands for the Fold Enrichment value of the GOID, it equals (Cour
#Column "Pvalue" stands for the significance testing value of the GOID
#Column "FDR" stands for the false discovery rate of the GOID, using Benjamini & Hochberg
#Column "Enrichment.Score" stands for the Enrichment Score value of the GOID, it equals (-lo
#Column "GENES" stands for the DE genes associated with the GOID

GO.ID	Term	Ontology	Count	Pop.Hits
GO:0044459	plasma membrane part	Cellular component	80	2120
GO:0030054	cell junction	Cellular component	29	590
GO:0005856	cytoskeleton	Cellular component	58	1500
GO:0005795	Golgi stack	Cellular component	9	107
GO:0031252	cell leading edge	Cellular component	12	181
GO:0005938	cell cortex	Cellular component	10	136
GO:0014069	postsynaptic density	Cellular component	7	83
GO:0031256	leading edge membrane	Cellular component	6	63
GO:0030027	lamellipodium	Cellular component	7	84
GO:0031253	cell projection membrane	Cellular component	9	129
GO:0016514	SWI/SNF complex	Cellular component	3	15
GO:0005911	cell-cell junction	Cellular component	12	209
GO:0015629	actin cytoskeleton	Cellular component	15	297
GO:0071944	cell periphery	Cellular component	125	4101
GO:0070603	SWI/SNF-type complex	Cellular component	3	21
GO:0031985	Golgi cisterna	Cellular component	6	81
GO:0005891	voltage-gated calcium channel complex	Cellular component	3	22
GO:0030864	cortical actin cytoskeleton	Cellular component	3	22
GO:0031984	organelle subcompartment	Cellular component	6	83
GO:0005622	intracellular	Cellular component	317	11619
GO:0042581	specific granule	Cellular component	2	10
GO:0017053	transcriptional repressor complex	Cellular component	4	45
GO:0034703	cation channel complex	Cellular component	8	140
GO:0031012	extracellular matrix	Cellular component	16	371
GO:0071564	npBAF complex	Cellular component	2	11
GO:0044463	cell projection part	Cellular component	17	404
GO:0032580	Golgi cisterna membrane	Cellular component	5	69
GO:0009897	external side of plasma membrane	Cellular component	8	144
GO:0009986	cell surface	Cellular component	16	375
GO:0070161	anchoring junction	Cellular component	9	172
GO:0005886	plasma membrane	Cellular component	120	4043
GO:0071565	nBAF complex	Cellular component	2	12
GO:0043197	dendritic spine	Cellular component	4	50
GO:0044424	intracellular part	Cellular component	307	11322

GO:0034704	calcium channel complex	Cellular component	3	30
GO:0044462	external encapsulating structure part	Cellular component	2	13
GO:0031227	intrinsic to endoplasmic reticulum membr	Cellular component	5	75
GO:0042995	cell projection	Cellular component	30	844
GO:0005912	adherens junction	Cellular component	8	154
GO:0044309	neuron spine	Cellular component	4	53
GO:0000228	nuclear chromosome	Cellular component	10	212
GO:0005777	peroxisome	Cellular component	6	102
GO:0005925	focal adhesion	Cellular component	6	102
GO:0042579	microbody	Cellular component	6	102
GO:0030312	external encapsulating structure	Cellular component	2	14

listed GOID

nt/Pop.Hits)/(List.Total/Pop.Total)

(1995) method
g10(Pvalue))

List.Total	Pop.Total	Fold.Enrichment	Pvalue	FDR	Enrichment.Score
425	16589	1.472941176	0.000212452	0.11026244	3.672739759
425	16589	1.918568295	0.000641602	0.166495729	3.192734266
425	16589	1.509273725	0.00099647	0.17238937	3.001535622
425	16589	3.283144585	0.001711756	0.222100397	2.766558033
425	16589	2.587819305	0.002484639	0.229079028	2.604736789
425	16589	2.870069204	0.002648312	0.229079028	2.577030777
425	16589	3.291934798	0.005378225	0.284968562	2.269361064
425	16589	3.717422969	0.005412828	0.284968562	2.266575754
425	16589	3.252745098	0.005741343	0.284968562	2.240986503
425	16589	2.723228454	0.005995696	0.284968562	2.222160398
425	16589	7.806588235	0.006039796	0.284968562	2.218977722
425	16589	2.241125809	0.007782532	0.336594517	2.108879074
425	16589	1.971360665	0.009890217	0.394847906	2.004794167
425	16589	1.189738514	0.014564109	0.516415333	1.836716088
425	16589	5.576134454	0.015761777	0.516415333	1.802394823
425	16589	2.891328976	0.017652464	0.516415333	1.753194657
425	16589	5.322673797	0.017910358	0.516415333	1.746895724
425	16589	5.322673797	0.017910358	0.516415333	1.746895724
425	16589	2.821658398	0.019682838	0.532709744	1.705912291
425	16589	1.06493178	0.020528314	0.532709744	1.687646721
425	16589	7.806588235	0.02571729	0.553759957	1.589774804
425	16589	3.469594771	0.027703703	0.553759957	1.557462182
425	16589	2.230453782	0.027805654	0.553759957	1.555866892
425	16589	1.683361345	0.030143376	0.553759957	1.520808113
425	16589	7.096898396	0.030907021	0.553759957	1.509942857
425	16589	1.642475248	0.031777195	0.553759957	1.497884443
425	16589	2.828473998	0.031804464	0.553759957	1.497511916
425	16589	2.168496732	0.032131864	0.553759957	1.493064074
425	16589	1.66540549	0.032785651	0.553759957	1.484316188
425	16589	2.042421341	0.033316521	0.553759957	1.477340354
425	16589	1.158533995	0.035725762	0.553759957	1.447018496
425	16589	6.505490196	0.036470063	0.553759957	1.438063486
425	16589	3.122635294	0.038801885	0.553759957	1.411147181
425	16589	1.058391887	0.040180331	0.553759957	1.395986487

425	16589	3.903294118	0.040659839	0.553759957	1.390834342
425	16589	6.005067873	0.042383978	0.553759957	1.372798286
425	16589	2.602196078	0.043221557	0.553759957	1.364299593
425	16589	1.387426819	0.044036105	0.553759957	1.3561911
425	16589	2.027685256	0.044899716	0.553759957	1.347756407
425	16589	2.945882353	0.046514762	0.553759957	1.3324092
425	16589	1.841176471	0.046894291	0.553759957	1.328880027
425	16589	2.296055363	0.047174179	0.553759957	1.32629565
425	16589	2.296055363	0.047174179	0.553759957	1.32629565
425	16589	2.296055363	0.047174179	0.553759957	1.32629565
425	16589	5.576134454	0.048627226	0.553759957	1.313120501

GENES
SCN5A//HLA-G//EFNA5//ADAM8//GPR64//CHRM1//CLCNKA//ABCC2//EFNA4//EPHB4//MLANA//FOLH1//L
ADD3//CTNND1//STEAP1//AQP3//PTPRK//TRPV4//ADAM15//CLDN24//SYNPO//AMOT//CLDN8//SORBS3//Z
DDX11//LOR//KRT5//KRT7//KRT8//AMOT//ADAM8//HOOK2//CASP8//USP2//SMEK2//SCYL1//MTUS1//FAM
PLD1//GOLGA2//NAGPA//GAL3ST4//ASAP2//B4GALT5//SULF1//RASIP1//MARCH9
AMOT//S100A11//CTNND1//PLD1//SCYL3//TRPV4//STXBP2//GABBR1//PTPRK//SYNE2//AIF1//WWC1
EXOC1//SPTBN4//TRPV4//SCNN1A//ADD3//FBLIM1//MAP2K2//AGTRAP//AXIN1//SCIN
CHRM1//SYNPO//SIPA1L1//CACNA1C//AXIN1//TANC1//P2RX6
GABBR1//SYNE2//AIF1//WWC1//TRPV4//PTPRK
SYNE2//CTNND1//AMOT//PLD1//SCYL3//TRPV4//STXBP2
GABBR1//SYNE2//SLC26A3//ATP6V0A4//ADCY6//MSN//AIF1//WWC1//TRPV4
RB1//SMARCB1//SMARCC2
CLDN24//SYNPO//AMOT//CLDN8//OBSL1//DSG4//ABCC2//ADD3//CTNND1//STEAP1//AQP3//PTPRK
AMOT//ADAM8//TNNT1//AIF1//SPTBN4//CAPZA1//TRPV4//SCNN1A//MYBPC1//APBB3//SYNPO//VILL//CO
CLDN24//ADAM8//GPR64//NOD1//RASL10A//ADCY6//CHRM1//SYNPO//CSMD2//NOSTRIN//ADD3//COL13
RB1//SMARCB1//SMARCC2
GOLGA2//NAGPA//GAL3ST4//ASAP2//B4GALT5//PLD1
CACNA1C//CACNA1D//CACNB4
SPTBN4//TRPV4//SCNN1A
PLD1//GOLGA2//NAGPA//GAL3ST4//ASAP2//B4GALT5
MGAT4B//EGFR//PLD1//SPPL2B//AGTRAP//RAP1GAP//RHBDF1//WLS//MARCH9//B4GALT5//HS3ST4//EXO
ADAM8//STXBP2
RLIM//PHF12//SMARCC2//TFAP4
SCN5A//CACNA1C//CACNA1D//CACNB4//KCTD13//KCNIP2//KCNK2//SCNN1A
COL4A5//ADAMTS8//CHI3L1//EFEMP1//ADAMTSL5//SERPINA1//WNT5A//LTBP4//COL13A1//LAMB2//SM
SMARCB1//SMARCC2
SPAG6//IFT122//CC2D2A//PPP1R9A//TRPV4//GABBR1//SYNE2//SLC26A3//ATP6V0A4//ADCY6//MSN//AIF1/
GOLGA2//NAGPA//GAL3ST4//ASAP2//B4GALT5
AMOT//ACE//ITGA2//BCAM//NRCAM//CD244//SCNN1A//THBS1
AMOT//ACE//ITGA2//BCAM//NRCAM//CD244//SCNN1A//THBS1//ADAM8//ELANE//EPHB4//SULF1//ALPP//
PTPRK//TRPV4//ADAM15//SORBS3//ZNF384//ARHGAP26//SYNE2//FBLIM1//DSG4
SCN5A//HLA-G//EFNA5//ADAM8//GPR64//CHRM1//CLCNKA//ABCC2//EFNA4//EPHB4//MLANA//FOLH1//L
SMARCB1//SMARCC2
SYNPO//GABBR1//SIPA1L1//P2RX6
MGAT4B//EGFR//PLD1//SPPL2B//AGTRAP//RAP1GAP//RHBDF1//WLS//MARCH9//B4GALT5//HS3ST4//EXO

CACNA1C//CACNA1D//CACNB4
GSTK1//AXIN1
ASPH//ELOVL1//DERL3//FMO4//FMO5
AMOT//S100A11//INHA//SPAG6//MSN//C6ORF170//IFT122//CC2D2A//TRPV4//ADAM15//CTNND1//PLD1//S[illegible]
SORBS3//ZNF384//ARHGAP26//SYNE2//FBLIM1//TRPV4//PTPRK//ADAM15
SYNPO//GABBR1//SIPA1L1//P2RX6
ADD3//REC8//NR1H3//DDX11//KDM4C//PCGF2//REPIN1//MCM7//PBRM1//SMARCB1
ACSL3//CROT//SCP2//SERHL2//GSTK1//KIAA0430
SORBS3//ZNF384//ARHGAP26//SYNE2//FBLIM1//TRPV4
ACSL3//SERHL2//GSTK1//CROT//SCP2//KIAA0430
GSTK1//AXIN1

PAR3//OPN3//GABBR1//STEAP1//TMPRSS11E//BCAM//NRCAM//KLRF1//ENPP3//PCDHA10//PTPRK//RG								
NF384//ARHGAP26//SYNE2//FBLIM1//OBSL1//DSG4//ABCC2//CHRM1//GABBR1//SIPA1L1//PCLO//NFIA								
125A//RB1//TNNT1//KIF25//SPAG6//TRPV4//AXIN1//AIF1//IFT122//CC2D2A//SPTBN4//CAPZA1//CHRM1/								
RO1B//MLPH//SORBS2								
A1//DSG4//CTNND1//DAB2IP//ACE//SLC26A3//DRP2//EFNA4//EFNA5//EGFR//AIF1//SYNE2//WWC1//FOL								
C1//PBRM1//SMARCB1//ADD3//TPR//REC8//MCM7//RB1//SMARCC2//NR1H3//DDX11//KDM4C//LOR//A								
C2//AXIN1//CPXM2//SLPI//TGFB2//THBS1								
/WWC1//SYNPO//SIPA1L1//P2RX6//CHRM1								
PTPRK//WNT5A//LPAR2								
PAR3//OPN3//GABBR1//STEAP1//TMPRSS11E//BCAM//NRCAM//KLRF1//ENPP3//PCDHA10//PTPRK//RG								
C1//PBRM1//SMARCB1//ADD3//TPR//REC8//MCM7//RB1//SMARCC2//NR1H3//DDX11//KDM4C//LOR//A								

CYL3//STXBP2//NRCAM//TGFB2//PIAS3//CHRM1//PPP1R9A//GABBR1//SYNE2//SLC26A3//ATP6V0A4//A								

R//CEACAM1//TNFSF11//MARCO//CD1C//P2RX6//LPAR2//ITGA2//ITGA3//ATP1A1//CACNA1C//CACNA1

//PPP1R9A//RAPSN//SNTB1//TANC1//P2RX6

/SYNPO//SIPA1L1//CACNA1C//TANC1//P2RX6//APBB3//VILL//CORO1B//MLPH//SORBS2//SCNN1A//MY

H1//LPAR3//ALPP//GABBR1//SIPA1L1//GPR110//STEAP1//GPR150//TMPRSS11E//GPR132//KCNIP2//HLA-

DAM15//KRT5//KRT7//KRT8//AMOT//PCGF2//ADAM8//SPAG6//IDUA//PON2//TSPAN8//USP4//DHRS2//A

R//CEACAM1//TNFSF11//MARCO//CD1C//P2RX6//LPAR2//ITGA2//ITGA3//ATP1A1//CACNA1C//CACNA1

DAM15//KRT5//KRT7//KRT8//AMOT//PCGF2//ADAM8//SPAG6//IDUA//PON2//TSPAN8//USP4//DHRS2//A

DCY6//AIF1//WWC1//MAPK8IP1//SYNPO//SIPA1L1//P2RX6								

D//CACNB4//EGFR//ADD3//CTNND1//AQP3//TRPV4//ADAM15//CLDN24//SYNPO//AMOT//CLDN8//SOR

BPC1//KRTAP10-1//KRTAP9-9//NDOR1//IP6K2//SORBS3//NOSTRIN//ADD3//DRP2//ARHGAP26//SYNE2//

G//OR2T8//AQP3//ITGA2//ITGA3//OR52N5//OR56A3//OR9G1//OR2T33//SPRED3//LRP5//BCAM//MSN//AT

OT7//ACSM1//CPT1B//ACSL3//SERHL2//CHRDL2//ACAD8//HMGCS2//ACADM//NMNAT3//ACADVL//N

D//CACNB4//EGFR//ADD3//CTNND1//AQP3//TRPV4//ADAM15//CLDN24//SYNPO//AMOT//CLDN8//SOR

OT7//ACSM1//CPT1B//ACSL3//SERHL2//CHRDL2//ACAD8//HMGCS2//ACADM//NMNAT3//ACADVL//N

BS3//ZNF384//ARHGAP26//SYNE2//FBLIM1//KCTD13//KCNIP2//KCNK2//ACE//CD244//SCNN1A//THBS1//
PCLO//TRIM67//MSN//FBLIM1//PPP1R9A//MICAL3//RAPSN//SNTB1//FUZ//SCIN
P1A1//ATP1B2//NRCAM//ATP6V0A4//VPS28//CD244//PIK3CD//SLCO1C1//PON2//PCDHA10//PMEPA1//PL
DUFS7//MCL1//MRPL2//SLC25A39//ATP5G2//ECHDC2//ENOSF1//OGDHL//MTUS1//ACSM3//SCP2//TAT//U
BS3//ZNF384//ARHGAP26//SYNE2//FBLIM1//KCTD13//KCNIP2//KCNK2//ACE//CD244//SCNN1A//THBS1//
DUFS7//MCL1//MRPL2//SLC25A39//ATP5G2//ECHDC2//ENOSF1//OGDHL//MTUS1//ACSM3//SCP2//TAT//U

LOR//OBSL1//SNTB1//NOD1//SLC26A3//MSN//ATP6V0A4//STXBP2//SLC9A3R2//AXIN1//DSG4//SIPA1L1								
SCR3//AGTRAP//MTUS1//PTPRK//RAPSN//TRPV4//SCNN1A//CEACAM1//STRA6//SLC26A6//STXBP2//VI								
JCP2//UCP3//ACSF2//CASP8//C20ORF72//SORBS3//NOD1//TAB1//CTNND1//AKR1C1//DUSP6//EEF1G//FA								
LOR//OBSL1//SNTB1//NOD1//SLC26A3//MSN//ATP6V0A4//STXBP2//SLC9A3R2//AXIN1//DSG4//SIPA1L1								
JCP2//UCP3//ACSF2//CASP8//C20ORF72//SORBS3//NOD1//TAB1//CTNND1//AKR1C1//DUSP6//EEF1G//FA								

//PCLO//NFIA//PPP1R9A//RAPSN//TANC1//CASP8//ADCY6//AIF1//WWC1//INHA								
LDLR//WNT5A//CACNA1C//CACNA1D//CACNB4//OR2A4//C6ORF25//OR51E2//AXIN1//CALN1//TAGLN2								
BP6//ARHGAP26//HAAO//SH2B1//AMPD1//CIDEB//NDOR1//HPGD//IMPDH1//MYBPC1//VPS28//FZR1//PI								
//PCLO//NFIA//PPP1R9A//RAPSN//TANC1//CASP8//ADCY6//AIF1//WWC1//INHA//RASL10A//CSMD2//NO								
BP6//ARHGAP26//HAAO//SH2B1//AMPD1//CIDEB//NDOR1//HPGD//IMPDH1//MYBPC1//VPS28//FZR1//PI								

//QRFPR//TANC1//TNFSF11//PPAP2C//ASAP2//CLDN8//CD1C//LPAR2//SLC9A3R2//SYTL4//MAGED1//EX								
PARG//FBLIM1//PGM2//PRKAA1//MAP2K2//SPTBN4//BCR//RPS6KA1//STXBP2//TNNT1//CACNB4//SPG1								
STRIN//COL13A1//DAB2IP//DRP2//ALPP//GPR110//GPR150//GPR132//OR2T8//OR52N5//OR56A3//OR9G1//								
PARG//FBLIM1//PGM2//PRKAA1//MAP2K2//SPTBN4//BCR//RPS6KA1//STXBP2//TNNT1//CACNB4//SPG1								

OC1//SCN5A//CLCNKA//ABCC2//EPHB4//MLANA//OPN3//KLRF1//ENPP3//RGR//MARCO//P2RX6//ADAM
//CAPZA1//AXIN1//PPP1R1B//TNK1//CCBL1//SELENBP1//MAPK8IP1//PAN2//CYP21A2//EPHX1//ALDH3
OR2T33//SPRED3//LRP5//ATP1B2//VPS28//PIK3CD//SLCO1C1//PON2//PMEPA1//PLSCR3//AGTRAP//MTU
//CAPZA1//AXIN1//PPP1R1B//TNK1//CCBL1//SELENBP1//MAPK8IP1//PAN2//CYP21A2//EPHX1//ALDH3

M15//AMOT//SORBS3//ZNF384//ARHGAP26//FBLIM1//MAP2K2//SPTBN4//SCIN//KCTD13//KCNK2//THBS								
A2//MLANA//SULF1//FMO4//FMO5//LRP5//DUOXA2//ASPH//MARCH2//TRAPPC1//ELOVL1//ALG13//C6C								
JS1//STRA6//SLC26A6//VLDLR//WNT5A//OR2A4//C6ORF25//OR51E2//CALN1//TAGLN2//QRFPR//PPAP2C								
A2//MLANA//SULF1//FMO4//FMO5//LRP5//DUOXA2//ASPH//MARCH2//TRAPPC1//ELOVL1//ALG13//C6C								

1//LOR//OBSL1//SNTB1//PCLO//NFIA//PPP1R9A//GSTK1//CASP8//INHA

RF25//CLDN8//DERL3//GSTK1//CROT//KIAA0430//RPS6KL1//ZNF737//SSX3//PIAS3//DDX17//ZNF266//M

C//ASAP2//SYTL4//MAGED1

RF25//CLDN8//DERL3//GSTK1//CROT//KIAA0430//RPS6KL1//ZNF737//SSX3//PIAS3//DDX17//ZNF266//M

YSM1//SERPINA3//IRX2//DAZL//ZNF579//AES//OIT3//ZNF384//AIF1//ZNF385C//ZBTB9//SCMH

YSM1//SERPINA3//IRX2//DAZL//ZNF579//AES//OIT3//ZNF384//AIF1//ZNF385C//ZBTB9//SCMH
