# Supplementary material for: LncRNAs as new biomarkers to differentiate triple negative breast cancer from non-triple negative breast cancer
Source: Oncotarget. 2016 Feb 19;7(11):13047–59. doi: 10.18632/oncotarget.7509 (PMC4914340; doi:10.18632/oncotarget.7509)
Supplement: Supplementary file 3 [file oncotarget-07-13047-s003.pdf]

#Column "GO.ID" stands for the ID of gene ontology term
#Column "Term" stands for the name of gene ontology term
#Column "Ontology" stands for the ontology of GOID belongs to
#Column "Count" stands for the number of DE genes associated with the listed GOID
#Column "Pop.Hits" stands for the number of background population genes associated with the
#Column "List.Total" stands for the total number of DE genes
#Column "Pop.Total" stands for the total number of background population genes
#Column "Fold.Enrichment" stands for the Fold Enrichment value of the GOID, it equals (Cou
#Column "Pvalue" stands for the significance testing value of the GOID
#Column "FDR" stands for the false discovery rate of the GOID, using Benjamini & Hochberg
#Column "Enrichment.Score" stands for the Enrichment Score value of the GOID, it equals (-lo
#Column "GENES" stands for the DE genes associated with the GOID

GO.ID	Term	Ontology	Count	Pop.Hits
GO:0008270	zinc ion binding	Molecular function	83	2016
GO:0043169	cation binding	Molecular function	138	3884
GO:0043167	ion binding	Molecular function	138	3895
GO:0046914	transition metal ion binding	Molecular function	89	2296
GO:0046872	metal ion binding	Molecular function	134	3842
GO:0005200	structural constituent of cytoskeleton	Molecular function	8	73
GO:0004180	carboxypeptidase activity	Molecular function	6	43
GO:0008092	cytoskeletal protein binding	Molecular function	27	531
GO:0008060	ARF GTPase activator activity	Molecular function	5	32
GO:0005096	GTPase activator activity	Molecular function	16	259
GO:0050660	flavin adenine dinucleotide binding	Molecular function	7	71
GO:0004181	metallocarboxypeptidase activity	Molecular function	4	25
GO:0003779	actin binding	Molecular function	17	325
GO:0003995	acyl-CoA dehydrogenase activity	Molecular function	3	16
GO:0070011	peptidase activity, acting on L-amino acid	Molecular function	25	557
GO:0048037	cofactor binding	Molecular function	14	257
GO:0008237	metallopeptidase activity	Molecular function	11	182
GO:0016798	hydrolase activity, acting on glycosyl bon	Molecular function	8	113
GO:0030695	GTPase regulator activity	Molecular function	21	456
GO:0008233	peptidase activity	Molecular function	25	576
GO:0051015	actin filament binding	Molecular function	5	54
GO:0004190	aspartic-type endopeptidase activity	Molecular function	3	20
GO:0070001	aspartic-type peptidase activity	Molecular function	3	20
GO:0008289	lipid binding	Molecular function	20	439
GO:0060589	nucleoside-triphosphatase regulator activi	Molecular function	21	468
GO:0005488	binding	Molecular function	343	11951
GO:0033293	monocarboxylic acid binding	Molecular function	5	58
GO:0008093	cytoskeletal adaptor activity	Molecular function	3	22
GO:0005504	fatty acid binding	Molecular function	4	39
GO:0008235	metalloexopeptidase activity	Molecular function	4	39
GO:0008047	enzyme activator activity	Molecular function	18	392
GO:0019902	phosphatase binding	Molecular function	5	60
GO:0060090	binding, bridging	Molecular function	8	130
GO:0050662	coenzyme binding	Molecular function	10	181

GO:0001786	phosphatidylserine binding	Molecular function	2	10
GO:0004953	icosanoid receptor activity	Molecular function	2	10
GO:0004954	prostanoid receptor activity	Molecular function	2	10
GO:0005391	sodium:potassium-exchanging ATPase ac	Molecular function	2	10
GO:0008271	secondary active sulfate transmembrane tr	Molecular function	2	10
GO:0050431	transforming growth factor beta binding	Molecular function	2	10
GO:0005083	small GTPase regulator activity	Molecular function	14	292
GO:0017124	SH3 domain binding	Molecular function	7	112
GO:0004553	hydrolase activity, hydrolyzing O-glycosy	Molecular function	6	89
GO:0016291	acyl-CoA thioesterase activity	Molecular function	2	11
GO:0046875	ephrin receptor binding	Molecular function	2	11
GO:0005245	voltage-gated calcium channel activity	Molecular function	3	27
GO:0008238	exopeptidase activity	Molecular function	6	92
GO:0016877	ligase activity, forming carbon-sulfur bon	Molecular function	3	28
GO:0001619	lysosphingolipid and lysophosphatidic aci	Molecular function	2	12
GO:0008556	potassium-transporting ATPase activity	Molecular function	2	12
GO:0015645	fatty acid ligase activity	Molecular function	2	12
GO:0016846	carbon-sulfur lyase activity	Molecular function	2	12
GO:0045125	bioactive lipid receptor activity	Molecular function	2	12
GO:0051721	protein phosphatase 2A binding	Molecular function	2	12
GO:0017016	Ras GTPase binding	Molecular function	6	94
GO:0015116	sulfate transmembrane transporter activity	Molecular function	2	13
GO:0003707	steroid hormone receptor activity	Molecular function	4	51

listed GOID

nt/Pop.Hits)/(List.Total/Pop.Total)

(1995) method
g10(Pvalue))

List.Total	Pop.Total	Fold.Enrichment	Pvalue	FDR	Enrichment.Score
416	15273	1.511536315	6.23223E-05	0.055155192	4.205356873
416	15273	1.30446036	0.000209347	0.060654004	3.679133382
416	15273	1.30077639	0.000239043	0.060654004	3.621523809
416	15273	1.423144976	0.000274142	0.060654004	3.562023803
416	15273	1.280496686	0.000622632	0.110205913	3.20576837
416	15273	4.023445732	0.000809962	0.119469366	3.09153546
416	15273	5.122875671	0.001022636	0.129290443	2.990278807
416	15273	1.866810626	0.00143685	0.157948178	2.842588658
416	15273	5.736553486	0.001606253	0.157948178	2.79418614
416	15273	2.268042768	0.002021705	0.178920912	2.694282168
416	15273	3.619684453	0.003138936	0.252541656	2.503217562
416	15273	5.874230769	0.004359418	0.321507078	2.360571486
416	15273	1.920421598	0.007974154	0.542855851	2.098315395
416	15273	6.883864183	0.008627565	0.545385351	2.064111766
416	15273	1.647843012	0.010210986	0.58679125	1.990932331
416	15273	1.999981293	0.011001652	0.58679125	1.95854211
416	15273	2.218974535	0.011271696	0.58679125	1.94801072
416	15273	2.599217155	0.012086013	0.594228966	1.917716948
416	15273	1.690773659	0.013641328	0.620927403	1.865143349
416	15273	1.593487079	0.015008324	0.620927403	1.823667796
416	15273	3.399439103	0.015506413	0.620927403	1.809488665
416	15273	5.507091346	0.016207665	0.620927403	1.790279541
416	15273	5.507091346	0.016207665	0.620927403	1.790279541
416	15273	1.672616962	0.017593308	0.620927403	1.754652497
416	15273	1.647420488	0.01772402	0.620927403	1.751437769
416	15273	1.053709498	0.01855454	0.620927403	1.731549799
416	15273	3.164995027	0.020582963	0.620927403	1.686492114
416	15273	5.006446678	0.021036387	0.620927403	1.677028843
416	15273	3.765532544	0.021048387	0.620927403	1.676781189
416	15273	3.765532544	0.021048387	0.620927403	1.676781189
416	15273	1.68584429	0.021955632	0.62679788	1.658454058
416	15273	3.059495192	0.02348456	0.63678451	1.629217577
416	15273	2.259319527	0.025885151	0.63678451	1.586949304
416	15273	2.028394603	0.026664048	0.63678451	1.574073919

416	15273	7.342788462	0.028818644	0.63678451	1.540326451
416	15273	7.342788462	0.028818644	0.63678451	1.540326451
416	15273	7.342788462	0.028818644	0.63678451	1.540326451
416	15273	7.342788462	0.028818644	0.63678451	1.540326451
416	15273	7.342788462	0.028818644	0.63678451	1.540326451
416	15273	7.342788462	0.028818644	0.63678451	1.540326451
416	15273	1.760257508	0.029500751	0.63678451	1.530166923
416	15273	2.294621394	0.033427638	0.668379156	1.475894305
416	15273	2.475097234	0.034510622	0.668379156	1.462047218
416	15273	6.675262238	0.034597814	0.668379156	1.46095134
416	15273	6.675262238	0.034597814	0.668379156	1.46095134
416	15273	4.079326923	0.036173634	0.668379156	1.441607863
416	15273	2.394387542	0.039616653	0.668379156	1.402122219
416	15273	3.933636676	0.039720345	0.668379156	1.400986986
416	15273	6.118990385	0.040782457	0.668379156	1.389526613
416	15273	6.118990385	0.040782457	0.668379156	1.389526613
416	15273	6.118990385	0.040782457	0.668379156	1.389526613
416	15273	6.118990385	0.040782457	0.668379156	1.389526613
416	15273	6.118990385	0.040782457	0.668379156	1.389526613
416	15273	6.118990385	0.040782457	0.668379156	1.389526613
416	15273	2.343443126	0.043273346	0.696307471	1.363779526
416	15273	5.648298817	0.047346311	0.748240806	1.324713854
416	15273	2.879524887	0.049725447	0.772053	1.3034213

GENES
ZNF737//NR1H3//ADAM8//PIAS3//ZNF266//ADAMTS8//PHF21B//ADAT3//AGAP1//CPXM2//CPO//CPB2//CPN
PGM2//RPS6KA1//ADAM8//SCGN//DSG4//OIT3//AIF1//EFEMP1//SULF1//PCLO//KCNIP2//ASPH//PCDHA10//
PGM2//RPS6KA1//ADAM8//SCGN//DSG4//OIT3//AIF1//EFEMP1//SULF1//PCLO//KCNIP2//ASPH//PCDHA10//
STEAP1//NDOR1//CP//HAAO//ZNF737//NR1H3//ADAM8//PIAS3//ZNF266//ADAMTS8//PHF21B//ADAT3//AG
PGM2//RPS6KA1//ADAM8//SCGN//DSG4//OIT3//AIF1//EFEMP1//SULF1//PCLO//KCNIP2//ASPH//PCDHA10//
SORBS3//ADD3//KRT5//LOR//MSN//VILL//SPTBN4//SORBS2
CPXM2//CPO//CPB2//CPN1//ACE//FOLH1
SYNPO//ADD3//ACE//SYNE2//MYBPC1//VILL//PPP1R9A//SPTBN4//TRPV4//SCNN1A//SNTB1//MLPH//CAPZ
AGAP1//AGAP9//AGAP8//AGAP5//ASAP2
C6ORF170//TBC1D30//DAB2IP//ARHGAP26//AGAP1//AGAP9//AGAP8//AGAP5//ASAP2//PCP2//SH3BP1//SIP
FMO4//FMO5//STEAP1//ACAD8//NDOR1//ACADM//ACADVL
CPXM2//CPO//CPB2//CPN1
EGFR//AIF1//CORO1B//TRPV4//SCIN//SYNPO//ADD3//ACE//SYNE2//MYBPC1//VILL//PPP1R9A//SPTBN4//S
ACADVL//ACADM//ACAD8
CASP8//USP2//ELANE//TMPRSS11E//KLK15//RHBDF1//LONRF2//ACE//FOLH1//CPXM2//CPO//CPB2//CPN1/
ACOT7//ACADVL//NDOR1//PDXDC1//TAT//CCBL1//OGDHL//NDUFS7//FMO4//FMO5//STEAP1//ACAD8//A
CPXM2//CPO//CPB2//CPN1//ADAM8//ADAMTS8//ADAMTSL5//ADAM15//MYSM1//ACE//FOLH1
LYZL4//IDUA//NAGPA//GBA//GLB1L//CHI3L1//SMPDL3B//ATHL1
SH2D3C//PCP2//BCR//MON2//ARHGAP26//SH3BP1//SIPA1L1//RAP1GAP//AXIN1//SYNGAP1//C6ORF170//TE
CASP8//USP2//ELANE//TMPRSS11E//KLK15//RHBDF1//LONRF2//ACE//FOLH1//CPXM2//CPO//CPB2//CPN1/
EGFR//AIF1//CORO1B//TRPV4//SCIN
SPPL2B//REN//NAPSA
SPPL2B//REN//NAPSA
ACOT7//ACADVL//THBS1//SCIN//CYP21A2//FABP6//LPAR3//PAFAH2//SYTL4//PCLO//NR1H3//AKR1C1//SC
SH2D3C//PCP2//BCR//MON2//ARHGAP26//SH3BP1//SIPA1L1//RAP1GAP//AXIN1//SYNGAP1//C6ORF170//TE
ACOT7//ACADVL//NOD1//STK25//DDX17//RASL10A//ADCY6//ACSM1//AGAP1//ABCC2//DAZL//DDX11//E
ACOT7//ACADVL//FABP6//AKR1C1//PPARG
ARHGAP26//OBSL1//SORBS2
ACOT7//ACADVL//PPARG//FABP6
CPXM2//CPO//CPB2//CPN1
PCP2//ARHGAP26//SH3BP1//SIPA1L1//RAP1GAP//BCR//AXIN1//SYNGAP1//C6ORF170//TBC1D30//DAB2IP/
CTNND1//EGFR//PPME1//DAB2IP//SLC9A3R2
LOR//SH2D3C//RUSC1//MAPK8IP1//ARHGAP26//OBSL1//SORBS2//DAB2IP
ACOT7//ACADVL//NDOR1//OGDHL//FMO4//FMO5//STEAP1//ACAD8//ACADM//HPGD

THBS1//SCIN							
PPARG//HPGD							
PPARG//HPGD							
ATP1A1//ATP1B2							
SLC26A3//SLC26A6							
THBS1//LTBP4							
MON2//BCR//C6ORF170//TBC1D30//DAB2IP//ARHGAP26//AGAP1//AGAP9//AGAP8//AGAP5//ASAP2//MLPH							
ZNF384//ARHGAP26//SH3BP1//PLSCR3//ADAM15//SYNGAP1//FAM125A							
LYZL4//IDUA//NAGPA//GBA//CHI3L1//GLB1L							
ACOT7//ACOT11							
EFNA4//EFNA5							
CACNB4//CACNA1C//CACNA1D							
ACE//FOLH1//CPXM2//CPO//CPB2//CPN1							
ACSM1//ACSL3//ACSM3							
LPAR3//LPAR2							
ATP1A1//ATP1B2							
ACSL3//ACSM1							
TAT//CCBL1							
LPAR3//LPAR2							
DAB2IP//PPME1							
RANBP3//KCTD13//MLPH//SYTL4//RPH3AL//RAP1GAP							
SLC26A3//SLC26A6							
NR1I3//NR1H3//PPARG//NR2F1							

N1//ZNF579//ACE//LONRF2//ZNF384//DRP2//ZNF385C//ZBTB9//KDM4C//PATZ1//PCLO//ZNF707//REPIN1/								
PLSCR3//S100A11//SMOC2//THBS1//VLDLR//CAPN3//CALN1//LTBP4//SCIN//CAPN13//ZNF737//NR1H3//								
PLSCR3//S100A11//SMOC2//THBS1//VLDLR//CAPN3//CALN1//LTBP4//SCIN//CAPN13//ZNF737//NR1H3//								
AP1//CPXM2//CPO//CPB2//CPN1//ZNF579//ACE//LONRF2//ZNF384//DRP2//ZNF385C//ZBTB9//KDM4C//PA								
PLSCR3//S100A11//SMOC2//THBS1//VLDLR//CAPN3//CALN1//LTBP4//SCIN//CAPN13//STEAP1//NDOR1/								
A1//SCIN//EGFR//AIF1//CORO1B//TNNT1//HOOK2//ARHGAP26//OBSL1//SORBS2//SORBS3//MAPK8IP1//								
A1L1//RAP1GAP//BCR//AXIN1//SYNGAP1								
CNN1A//SNTB1//MLPH//CAPZA1								
/SPPL2B//REN//NAPSA//CAPN3//CAPN13//ATG4B//USP4//ADAM8//ADAMTS8//ADAMTSL5//ADAM15//N								
CADM//HPGD								
BC1D30//DAB2IP//AGAP1//AGAP9//AGAP8//AGAP5//ASAP2//MLPH//SYTL4//RPH3AL								
/SPPL2B//REN//NAPSA//CAPN3//CAPN13//ATG4B//USP4//ADAM8//ADAMTS8//ADAMTSL5//ADAM15//N								
CP2//PLD1//PPARG//OSBPL9//FABP7//EPN2//ATP5G2//LPAR2								
BC1D30//DAB2IP//AGAP1//AGAP9//AGAP8//AGAP5//ASAP2//MLPH//SYTL4//RPH3AL								
GFR//EPHB4//ACSL3//LACE1//PRKD2//GMPPA//NMNAT3//KIF25//RASL11A//MCM7//ATP1A1//IP6K2//PI								
/AGAP1//AGAP9//AGAP8//AGAP5//ASAP2//NOD1//TAB1								

I//SYTL4//RPH3AL								

/CXXC1//ZBTB48//ADAMTSL5//ZNF517//ZNF710//TRIM73//TRIM67//TRIM17//RLIM//MARCH2//ZNF571/

PIAS3//STK25//ZNF266//ADAMTS8//ADCY6//MGAT4B//PHF21B//ADAT3//MYSM1//ACSM1//AGAP1//ADI

PIAS3//STK25//ZNF266//ADAMTS8//ADCY6//MGAT4B//PHF21B//ADAT3//MYSM1//ACSM1//AGAP1//ADI

ATZ1//PCLO//ZNF707//REPIN1//CXXC1//ZBTB48//ADAMTSL5//ZNF517//ZNF710//TRIM73//TRIM67//TRIM

/CP//HAAO//ZNF737//NR1H3//PIAS3//ZNF266//ADAMTS8//PHF21B//ADAT3//AGAP1//CPXM2//CPO//CPB2

/NRCAM//MSN//RPH3AL

MYSM1

MYSM1

K3CD//PRKAA1//MAP2K2//BCR//RPS6KA1//ACSM3//WNK2//TPR//ACSF2//RPS6KL1//CDK10//STK16//TNI

/ANKMY1//PLAGL1//BCL11A//PPARG//FBLIM1//ZNF434//RNF125//ZDHHC7//ZNF512B//ZNF608//MICAL

D3//CPO//CP//CPB2//CPN1//CYP21A2//ZNF579//ACE//LONRF2//ZNF384//DRP2//ZNF385C//ZBTB9//KDM4C

D3//CPO//CP//CPB2//CPN1//CYP21A2//ZNF579//ACE//LONRF2//ZNF384//DRP2//ZNF385C//ZBTB9//KDM4C

I17//RLIM//MARCH2//ZNF571//ANKMY1//PLAGL1//BCL11A//PPARG//FBLIM1//ZNF434//RNF125//ZDHH

2//CPN1//ZNF579//ACE//LONRF2//ZNF384//DRP2//ZNF385C//ZBTB9//KDM4C//PATZ1//ZNF707//REPIN1//

K1//KIAA0430//NR1H3//ADAM8//SORBS3//PIAS3//TAB1//HHLA3//CHRM1//SYNPO//CSMD2//NOSTRIN//

3//PHF12//RAPSN//BCL6//PRDM1//AGAP9//C11ORF95//NR2F1//AGAP8//AGAP5//ZNF3//ZNF26//ZNF33A//

C//FOLH1//HAAO//PATZ1//ALPP//PHYHD1//PRKD2//STEAP1//AMPD1//ZNF707//REPIN1//HBE1//CXXC1//

C//FOLH1//HAAO//PATZ1//ALPP//PHYHD1//PRKD2//STEAP1//AMPD1//ZNF707//REPIN1//HBE1//CXXC1//

C7//ZNF512B//ZNF608//MICAL3//PHF12//RAPSN//BCL6//PRDM1//AGAP9//C11ORF95//NR2F1//AGAP8//AG

CXXC1//ZBTB48//ADAMTSL5//ZNF517//ZNF710//TRIM73//TRIM67//TRIM17//RLIM//MARCH2//ZNF571//A

AP3S1//SERPINA3//COL4A5//COL13A1//CP//CTNND1//AMOT//ACE//AES//RASSF6//EEF1G//EFNA5//ELA

/ZNF75D//ZNF79//ZNF132//PCGF2//MLPH//ZNF426//ZNF613//ZNF696//ZNF442//ZMYND12//ZNF414//SOR

ZBTB48//ZNF517//IMPDH1//NDUFS7//ZNF710//TRIM73//TRIM67//ATP1A1//TRIM17//RLIM//MARCH2//ZN

ZBTB48//ZNF517//IMPDH1//NDUFS7//ZNF710//TRIM73//TRIM67//ATP1A1//TRIM17//RLIM//MARCH2//ZN

GAP5//ZNF3//ZNF26//ZNF33A//ZNF75D//ZNF79//ZNF132//PCGF2//MLPH//ZNF426//ZNF613//ZNF696//ZNF

ANKMY1//PLAGL1//BCL11A//PPARG//FBLIM1//ZNF434//RNF125//ZDHHC7//ZNF512B//ZNF608//MICAL3

NE//ENSA//EFEMP1//PDXDC1//ARHGAP26//MLANA//ATG4B//KIAA0182//SYNE2//WWC1//SH3BP1//GAB

BS2//ZNF577//ADAM15//ASAP2//MTMR3//ZMYM3//ZNF764//ZBTB22//MARCH9//SYTL4//RPH3AL//ZBTB

NF571//ANKMY1//ENPP3//PLAGL1//BCL11A//PON2//PPARG//FBLIM1//ZNF434//RNF125//ENOSF1//PRKA

NF571//ANKMY1//ENPP3//PLAGL1//BCL11A//PON2//PPARG//FBLIM1//ZNF434//RNF125//ENOSF1//PRKA

442//ZMYND12//ZNF414//SORBS2//ZNF577//ADAM15//ASAP2//MTMR3//ZMYM3//ZNF764//ZBTB22//MA

//PHF12//RAPSN//BCL6//PRDM1//AGAP9//C11ORF95//NR2F1//AGAP8//AGAP5//ZNF3//ZNF26//ZNF33A//Z

BR1//GBA//NDOR1//GOLGA2//ZNF707//HOOK2//TNPO2//KCNIP2//CXXC1//HOXA9//INHA//ITGA2//ITGA

40//NR1I3

A1//ZDHHC7//THAP10//ZNF512B//ZNF608//MICAL3//PHF12//RAPSN//BCL6//ACSM3//PRDM1//AGAP9//N

A1//ZDHHC7//THAP10//ZNF512B//ZNF608//MICAL3//PHF12//RAPSN//BCL6//ACSM3//PRDM1//AGAP9//N

RCH9//SYTL4//RPH3AL//ZBTB40//NR1I3//CYP21A2//HBE1

NF75D//ZNF79//ZNF132//PCGF2//MLPH//ZNF426//ZNF613//ZNF696//ZNF442//ZMYND12//ZNF414//SORB

3//GSTK1//NDUFS7//KRT5//KRT7//KRT8//LOR//LRP5//MAGEA6//MCL1//MKLN1//MSN//ATP1B2//ATP6V

R2F1//AGAP8//AGAP5//ZNF3//ZNF26//ZNF33A//ZNF75D//ZNF79//ZNF132//PCGF2//MLPH//ZNF426//ZNF6

R2F1//AGAP8//AGAP5//ZNF3//ZNF26//ZNF33A//ZNF75D//ZNF79//ZNF132//PCGF2//MLPH//ZNF426//ZNF6

S2//ZNF577//ADAM15//ASAP2//MTMR3//ZMYM3//ZNF764//ZBTB22//MARCH9//SYTL4//RPH3AL//ZBTB4

0A4//TRIM17//VPS28//FZR1//CD244//SERPINA1//PPARG//PBRM1//ZDHHC7//FAM63A//PCDHA10//PLSCI

13//ZNF696//ZNF442//POLR1B//ZMYND12//FLYWCH1//ZNF414//ZNF577//ADAM15//ASAP2//MTMR3//US

13//ZNF696//ZNF442//POLR1B//ZMYND12//FLYWCH1//ZNF414//ZNF577//ADAM15//ASAP2//MTMR3//US

0//NR1I3//CYP21A2//HBE1//STK25//ADCY6//MGAT4B//MYSM1//ACSM1//ADD3//FOLH1//ALPP//PHYHD

R3//AGTRAP//SCYL3//CORO1B//SMEK2//PHF12//SPTBN4//PTPRK//RAP1GAP//RB1//TRPV4//RGR//BCL6//

P2//ZMYM3//ZNF764//ZBTB22//MARCH9//B4GALT5//SYTL4//RPH3AL//ZBTB40//NR1I3//NDOR1//CPXM								
P2//ZMYM3//ZNF764//ZBTB22//MARCH9//B4GALT5//SYTL4//RPH3AL//ZBTB40//NR1I3//NDOR1//CPXM								
//PRKD2//AMPD1//IMPDH1//NDUFS7//ATP1A1//ENPP3//PON2//ENOSF1//PRKAA1//THAP10//ACSM3//PC								
S100A11//SCN5A//SCNN1A//SCP2//ELOVL1//SMARCB1//SMARCC2//SNTB1//TAF6//TFAP4//TFE3//TGFB								

2//ADAMTSL5//C11ORF95//SORBS2//CHI3L1//GBA//IDUA//GLB1L							
2//ADAMTSL5//C11ORF95//SORBS2//CHI3L1//GBA//IDUA//GLB1L							
LR1B//FLYWCH1//USP2//B4GALT5							
2//THBS1//NKX2-1//USP4//VLDLR//ZNF3//CACNA1C//CSDE1//CACNB4//C19ORF57//NHEJ1							
