# Supplementary material for: LncRNAs as new biomarkers to differentiate triple negative breast cancer from non-triple negative breast cancer
Source: Oncotarget. 2016 Feb 19;7(11):13047–59. doi: 10.18632/oncotarget.7509 (PMC4914340; doi:10.18632/oncotarget.7509)
Supplement: Supplementary file 4 [file oncotarget-07-13047-s004.docx]

**Supplemental Material S2. Primers for qRT-PCR of lncRNAs**

| Seqname | Gene symbol | Chromosome location | Primers  (5’-3’, Forward: F，Reverse: R) |
| --- | --- | --- | --- |
| ENST00000420269 | Z82214.3 | Chr22 +:43608679 ~ 43609667 | F: AGAGAGTATTCTGGCATTGAGTGAGA R: TGTTTGGGTAGAAGCTGAGAATCC |
| ENST00000561529 | RP11-432I5.1 | Chr16 +:71599691 ~ 71612090 | F: CTAATCTCATCCTTTGTATATTCGTCTTG  R: GGAGCTCCACTGATGTCTCTCA |
| ENST00000451190 | RP11-414K1.3 | Chr10 +: 17450025 ~ 17455502 | F: AGAGTAACCCCCATCCATCATG  R: TTTGTCGGGCTGGATCCA |
| NR_046371 | CP | Chr3 -: 148880196 ~ 148939832 | F: GCGACAGCTGGGCTGAATAT  R: CATGGCGCTAATGCTAATGGT |
| uc001tfa.1 | RMST | Chr12 +: 97887668 ~ 97958793 | F: TGAAGACCATTTTATGCATCACTCA  R: GTGGTGGTTTCAGCCAGTCTTAT |
| ENST00000568797 | CHST9-AS1 | Chr18 +: 24445271 ~ 24515910 | F: CTCCCAAGAGCTGGTGTAGAATG  R: CTTGAAGAAGACGCTGAAGAGAAA |
| ENST00000541282 | RMST | Chr12 +: 97858798 ~ 97927544 | F: CACCTTTGAGAAAGAAAAGACTGCTT  R: AACTATTGATCCAACCTAGGCATTTAG |
| ENST00000515227 | RP11-434D9.1 | Chr5 +: 67089047 ~ 67101066 | F: AAACTCCAGGATGCTCCATCA  R: GTCCTCATAGCCTTTCCTACTTTACTTC |
| NR_038899 | DSCAM-AS1 | Chr21 +: 41755010 ~ 41757285 | F: GCTCTGCTCAGTGTCGCTACA  R: CAAGGATCATGATGAGACCAGAAC |
| uc021vkv.1 | IGKV | Chr2 +: 89197030 ~ 89197301 | F: ACTCCAGGAGACAAAGTCAACATCT  R: TGGTTTCTGTTGGTACCAGTTCA |
| ENST00000560153 | LINC00052 | Chr15 +: 88120159 ~ 88122917 | F: CCCAAGACCAGCTACCTAACCA  R: TGGGAACCAGGCCTTCCT |
| NR_038378 | LOC441242 | Chr7 -: 65111923 ~ 65235797 | F: CAAGGGATCCAGGACAGGATAA  R: GCCTTGTGTTTGTCTTAAAAGTTCAT |
| ENST00000505050 | RP11-503D12.1 | Chr5 -: 44698532 ~ 44700910 | F: TTATGTCAGAAATGGGTAAAAGATGTG  R: GTAACTTCTCAGAGTCCAAGGTCAGT |
| ENST00000453568 | RP4-781K5.4 | Chr1 +: 234809841 ~ 234818922 | F: ACAAAAGAAGGAATACCAGCAAGAC  R: CGGCCAAATTCTTCAGGAAA |
| ENST00000533504 | RP11-839D17.3 | Chr11 -: 113150104 ~ 113185159 | F: TGAAGGCCAAAGTCGAGGTT  R: GTAGAGATGCATAGTAGCATGTCATTA |
| uc002suw.1 | BC016831 | Chr2 +: 96331831 ~ 96334463 | F: TCAGACCTGCCCCTGGAA  R: AGGAGCATTGTCCAGCTGTTC |
| ENST00000509201 | RP11-93K22.6 | Chr3 -: 129672947 ~ 129688137 | F: GGATTACAGAACAGAGGGAATTTCC  R: CAGCATCCACTGTGCTCTATGC |
| ENST00000565519 | RP11-384L8.1 | Chr3 -: 32278179 ~ 32280070 | F: GCTGTGGAAATGGCAGGAAT  R: GCGGCCTCTCATCGATCTC |
| NR_038925 | LOC100288181 | Chr8 -: 143783669 ~ 143808391 | F: CCGGAGATGAAATGTGGCTTA  R: CCACGCCCACCTCTAGGTAGT |
| ENST00000566734 | RP11-1021N1.2 | Chr16 +: 15506576 ~ 15507125 | F: TGTGTTAGCCAGGATGGTCTCA  R: CACACCTGTAATCCCAGCACTCT |
| ENST00000451607 | GAS5 | Chr1 -: 173834661 ~ 173836867 | F: GCCAACCGCTCTGATGGT  R: GGGTCACGGCCCTTAACAAT |
| ENST00000432536 | GAS5 | Chr1 -: 173834996 ~ 173836827 | F: TGGCGAGACTGGTCTTGAACT  R: CGGCTTGTAATCCCAGTATGTTG |
| ENST00000531708 | AP001258.4 | Chr11 -: 58902762 ~ 58910488 | F: TGGCATGATCTTGGCTTACTGA  R: CAAGTGGCTGAGGCATGAGA |
| ENST00000309874 | RP11-23P13.6 | Chr15 +: 42184990 ~ 42188133 | F: CGCACTGGAAGACGTTATTGATC  R: GCCACATTCGCAAGCTACAG |
| ENST00000499554 | CTD-2547L16.1 | Chr8 +: 17942376 ~ 17953903 | F: GGCCAAGCTGGTCTTGAACTC  R: ATGGTGGCTCATGCCTGTAAT |
| ENST00000503709 | NOP14-AS1 | Chr4 +: 2938992 ~ 2963465 | F: CACTGGCTTCCTTGCTTTGC  R: GTTTCCACGCTCTTGGTAGGAT |
| ENST00000569782 | RP11-452L6.7 | Chr16 +: 31519791 ~ 31520569 | F: AGATTGCAATGATGTCACAAAACA  R: GGATGCTTTTTTGGCAAATTTC |
| ENST00000547326 | RP11-585P4.5 | Chr12 -: 75877903 ~ 75883600 | F: TTCCATTACCCAAATCCTATAAAACAG  R: TACAGGCAGGCTGAGTTCAAAA |
| ENST00000545440 | SNHG1 | Chr11 -: 62619723 ~ 62622960 | F: GCACGTTGGAACCGAAGAGA  R: TTGTCCCATTCCTTCACCAGTA |
| ENST00000541578 | SNHG1 | Chr11 -: 62619768 ~ 62622960 | F: GCACGTTGGAACCGAAGAGA  R: TTGTCCCATTCCTTCACCAGTA |
| uc002zis.1 | MCM3AP-AS1 | Chr21 +: 47656137 ~ 47671743 | F: GCGCCGCGCTTTGAG  R: GGATCTGATGCGAGGAGCTT |
| ENST00000557691 | RP6-65G23.3 | Chr14 +: 71277046 ~ 71282120 | F: GAGATAGGAGGCCCATAATGTTTC  R: CTGCAACCAGGTGGAAGTCA |
| ENST00000539975 | SNHG1 | Chr11 -: 62619491 ~ 62622708 | F: GGGATTGCAGGTGTGATTCAT  R: ACAGTGCCTGAGTTTGGGTTCT |
| ENST00000563632 | RP11-757F18.5 | Chr3 +: 111852269 ~ 111854206 | F: CCAACAGGGCAGGGTGTAA  R: TGCCATCCGTGGACAGCTA |
| uc003ysl.3 | PVT1 | Chr8 +: 128902834 ~ 129113499 | F: CAGAATCCGTGTCTGGGAGAA  R: CAGGCCTCCAAGATGTAACTCAT |
| ENST00000457996 | RP11-439A17.9 | Chr1 +: 120925633 ~ 120933000 | F:AGCAGACATTACTAATTTCCTACTCAACA  R: TGGACCCGAGCCCTAATCA |
| NR_045637 | BOLA3-AS1 | Chr2 +: 74375107 ~ 74376181 | F: CCTCTCCTCTCTCTAGCTGTTATTTGT  R: TGGCAAAAAGCTGGTAAATCCT |
| ENST00000428289 | RP11-196G18.3 | Chr1 -: 149757214 ~ 149764576 | F:AGCAGACATTACTAATTTCCTACTCAACA  R: TGGACCCGAGCCCTAATCA |
| NR_027783 | SAT1 | ChrX +: 23801274 ~ 23804327 | F: GCAAAGGGAAGAAAAGCAAAAG  R: TGATCAGCCGCAGTATGTCACT |
| ENST00000540904 | SNHG1 | Chr11 -: 62622151 ~ 62622960 | F: GCCGGCGGAACAATGTT  R: CGATCTCCCTCCCCAGAAAA |
| NR_027170 | C17orf76-AS1 | Chr17 +: 16342300 ~ 16345340 | F: GGCGGAAAGTTCCCATGA  R: CGGAATATCCCAATCCTCACA |
| ENST00000537068 | SNHG1 | Chr11 -: 62619459 ~ 62623353 | F: GCACGTTGGAACCGAAGAGA  R: TTGTCCCATTCCTTCACCAGTA |
| ENST00000464612 | SNHG12 | Chr1 -: 28905049 ~ 28907522 | F: TTTTTCTGGTGATCGAGGACTTC  R: GCTCACCTCCTCAGTATCACACACT |
| ENST00000483140 | C17orf76-AS1 | Chr17 +: 16342640 ~ 16345337 | F: AGGCTCCTGGGCTCAGATG  R: GCAAGGGACAAAGCTACAACTTG |
| NR_024127 | SNHG12 | Chr1 -: 28905049 ~ 28908366 | F: GCCAGGATGGTCTCGATCTC  R: CCTGTAATCCCAGCACTTTGG |
| ENST00000520885 | RP11-809O17.1 | Chr8 -: 142136142 ~ 142138220 | F: CCCAGCCAGCAGGAACAT  R: CCAATCCGAGCTTTGCTTTC |
| ENST00000514146 | CTC-338M12.3 | Chr5 +: 180688212 ~ 180691262 | F: ATTCCCAAGCCCCTTCTCA  R: CCTTCAACAGCATTTTACTGATCAG |
| NR_034166 | NDUFA11 | Chr19 -: 5894680 ~ 5904024 | F: GGCTAAGGTTGGACAATACACGTT  R: GGGCTTCTCGCGGACAT |
| NR_027168 | C17orf76-AS1 | Chr17 +: 16342300 ~ 16345340 | F: AGGCTCCTGGGCTCAGATG  R: GCAAGGGACAAAGCTACAACTTG |
| ENST00000576912 | RP13-638C3.4 | Chr17 -: 80560864 ~ 119818033 | F: ATCGCGTGTGCCGAGTGT  R: TGTTCCCATTTTAGCGTCTGAGA |
| ENST00000584252 | RP11-15F12.1 | Chr18 +: 46550072 ~ 46574844 | F: CCTGAAGGCATCAGTAATCTGGTT  R: GCTCTGTACCTCACACCCAGAAG |
| ENST00000510619 | RP1-68D18.2 | Chr11 +: 35234096 ~ 35235554 | F: TGTTGTGCAACCCAATGAATTT  R: GGACGGAGCCTCGTTCTGT |
| ENST00000428008 | ZNFX1-AS1 | Chr20 +: 47895178 ~ 47905797 | F: GCAGTCAGGCTTCATACGCTATT  R: CCGCTGGCTGCTCTAACC |
| NR_024334 | FUT8-AS1 | Chr14 -: 65877310 ~ 65879335 | F: AAAAATTAGCCGGGCGTAGTG  R: CCTGGGTTCACGCCATTCT |
| ENST00000561761 | RP11-629O1.2 | Chr8 +: 134584413 ~ 134586104 | F: GAGTTCTGCTTACATCGGCTACTG  R: TCTGGTGCATGTTAGAAGCTCTTC |
| ENST00000429167 | FAM83C-AS1 | Chr20 +: 33873053 ~ 33873559 | F: TTCTCTCAAAATGTCACAGTGGTAACT  R: CCTGGAGGAGGGTGAATGC |
